# Exploring the impact path of school connectedness on student development through cultivating students’ social-emotional skills

**DOI:** 10.1371/journal.pone.0353972

**Published:** 2026-07-22

**Authors:** Yukai Wei, Xinyi Wang

**Affiliations:** 1 Faculty of education, Beijing Normal University, Beijing, China; 2 Beijing No.18 High School, Beijing, China; University of Genoa School of Social Sciences: Universita degli Studi di Genova Scuola di Scienze Sociali, ITALY

## Abstract

Based on OECD’s Survey on Social and Emotional Skills (SSES) Asian data (n = 23560), this study examines the pathways through which school connectedness relates to students’ positive development using structural equation modeling. The findings reveal that school connectedness predicts student positive development through serial effect of interpersonal skills and emotion regulation skills. School connectedness is associated with social-emotional skills (student engaging with others). These two social-emotional skills both predict students’ positive development. Student-classmate relationship and perceived relationship with teachers contribute to school connectedness. Energy, stress resistance, and optimism are important social-emotional skills. The outcomes are primarily reflected in positive emotions at school and students’ school engagement. The study elucidates the internal mechanism through which the school environment predicts students’ development via a sequence of social-emotional skills, offering meaningful implications for optimizing school connectedness and cultivating student’s social-emotional skills.

## Introduction

In recent decades, education systems worldwide have increasingly recognized the importance of fostering student social and emotional skills (SES) alongside academic achievement. The OECD’s Survey on Social and Emotional Skills (SSES) represents a significant international effort to assess and understand the development of these competencies among youth. Within Asian contexts participating in SSES, unique cultural and educational dynamics shape the manifestation and development of SES. These regions often emphasize academic excellence, yet there is growing awareness of the need to support holistic student development, particularly through enhancing school connectedness. School connectedness, defined as students’ belief that adults and peers in their school care about their learning and well-being, serves as a critical contextual factor. In Asian educational settings, where collective values and hierarchical relationships relate to student-teacher interactions, understanding how school connectedness predicts SES and subsequent positive development is crucial. This paper explores the interplay between school connectedness, key social-emotional skills, and positive student outcomes within the Asian SSES context, addressing a vital area for educational improvement and student well-being [1].

Existing research has largely examined the pairwise relationships and constituent elements between school connectedness, students’ social-emotional skills, and student development. However, studies integrating the tripartite relationships and holistic pathways remain relatively scarce.

First, research on the impact of school connectedness on student development indicates that school connectedness predicts students’ social-emotional skills, such as social competence and emotional regulation, thereby promoting positive developmental outcomes like academic engagement and well-being. These findings align with theoretical frameworks such as Social Cognitive Theory [2]. Teacher-student relationships and peer relationships are core components of school connectedness, playing complex mediating roles between emotional regulation and academic engagement. School connectedness and positive peer relationships have been identified as crucial environmental foundations for the development of students’ social-emotional skills (e.g., interpersonal skills, emotional regulation), which in turn foster positive student development [3].

Second, in studies concerning the school environment and students’ social-emotional skills, particularly emotional regulation and interpersonal skills, teacher-student and peer relationships serve as key contextual factors influencing student development, including positive emotions, academic engagement, and learning motivation [4]. Teacher-student relationships, especially closeness and teacher support, are central to enhancing students’ academic engagement, well-being, and prosocial behavior [5–7]. Teachers’ own social-emotional competencies are considered critical mechanisms that directly affect the quality of teacher-student interactions and the development of students’ skills [8]. Emotional regulation skills act as a core mediating mechanism linking social support to positive developmental outcomes, such as academic engagement, emotional resilience, and learning motivation [9]. Additionally, self-regulated learning, reflective practice, personalized feedback, and learner agency play significant roles in optimizing learning experiences and emotional outcomes [10]. However, empirical validation of the complete pathway using objective data remains an area requiring further innovative exploration.

The research question of this study are: Is school connectedness positively associated with student development through the mediating roles of social-emotional skills (including student engaging with others and student emotion regulation)? What is the nature of this pathway?

Empirical model in this study is designed to test a specific, sequential pathway of influence, namely, to explore relationship between school connnectedness and interpersonal skills, which in turn relates to emotional regulation skills, ultimately contributing to positive student development. This represents a causal chain model proposed for examination from a particular theoretical perspective (Social Cognitive Theory). While it does not deny the existence of bidirectional relationships, it focuses on testing whether this specific directional pathway is significant and quantifying its effect size.

This study addresses a significant gap in the existing literature by investigating the sequential mechanism through which school connectedness relates to student positive development via multidimensional social-emotional skills. Prior research has predominantly focused on the direct association between school connectedness and student development outcomes, or has examined mediating effects within a single dimension, such as emotion regulation. Consequently, there remains insufficient exploration of the synergistic effects among various skills and their developmental trajectories. To fill this gap, the present study employs structural equation modeling to empirically validate a sequential mediation pathway: school connectedness → engaging with others social-emotional skills → emotion regulation social-emotional skills → positive development.

## Literature review

The role of student school connectedness in students’ social-emotional skills.

Student school connectedness provides a secure and supportive social environment, which serves as crucial soil for the development of students’ social-emotional skills.

In terms of students’ Engaging with Others skills, positive teacher-student relationships and peer relationships are the core influencing factors. High-quality teacher-student relationships, characterized by teacher care, support, and respect, directly provide students with role models for social interaction and a sense of security, thereby encouraging students to engage in positive social attempts and enhancing their abilities in cooperation, empathy, and conflict resolution [11]. When students feel accepted and supported by their teachers, they are more willing to participate in classroom interactions and establish positive peer relationships [12]. Concurrently, a harmonious classroom climate and positive peer connectedness function as a micro-level social practice field where students learn how to build and maintain friendships and navigate interpersonal relationships, thereby exercising and enhancing their social confidence and interaction skills [13]. Students who perceive a strong sense of school connectedness demonstrate stronger prosocial behaviors and collaborative abilities.

Regarding students’ Emotion Regulation skills, school connectedness serves as an “emotional scaffold”. Teacher feedback, particularly when it is positive and constructive, helps students better understand and manage their emotions. When students perceive teacher feedback as fair and supportive, they are more likely to seek help in the face of challenges rather than succumb to negative emotions [14]. Supportive teacher-student relationships and the classroom environment themselves constitute a significant emotional resource. When students are in a classroom where they feel psychological safety, they are more capable of expressing emotions openly and learning to use adaptive strategies, such as cognitive reappraisal, to regulate negative emotions like anxiety and anger [7]. Through co-regulation strategies, such as validating students’ emotional experiences and guiding problem-solving, teachers can effectively support students, particularly those in lower grades, in developing their emotion regulation abilities [15]. Conversely, students with weak school connectedness may be more prone to experiencing difficulties in emotion regulation [16].

### The role of student school connectedness in student development

Student school connectedness is a strong predictor of students’ subjective well-being. It shows a significant positive correlation with students’ mental health levels and effectively reduces the risk of internalizing problems such as depression and anxiety. The underlying mechanism is that the fulfillment of the need for connectedness is a fundamental human psychological requirement. When students feel cared for, accepted, and valued within the school environment, their sense of well-being is naturally enhanced. This protective effect of school connectedness is particularly pronounced for marginalized groups, such as sexual minority students and students of color, making it a key factor in promoting mental health equity [5].

Moreover, student school connectedness directly nurtures students’ positive emotions at school. Supportive teacher-student interactions and a positive classroom environment serve as significant sources of positive emotions, such as enjoyment, interest, and pride [17]. When students establish positive relationships with teachers and receive encouraging feedback from them, they experience greater learning enjoyment and a stronger sense of accomplishment [18]. Teachers’ conscious use of extrinsic emotion regulation strategies, such as humor and praise, can elicit students’ experiences of positive emotions, thereby fostering a more positive learning climate [14].

In addition, student school connectedness serves as a core mechanism for sustaining and enhancing student school engagement. Behavioral, emotional, and cognitive engagement are all closely associated with school connectedness. Students’ perceived teacher support and care significantly predict higher levels of classroom participation and learning concentration, while reducing behavioral disaffection. Emotionally, student school connectedness and a sense of peer relatedness make students more willing to engage in school activities [19]. During the COVID-19 pandemic, the reason why face-to-face learning modalities were more effective than remote or hybrid models in promoting student academic engagement was precisely because they were most conducive to maintaining students’ sense of connectedness with teachers and peers. Furthermore, this sense of connectedness fully mediated the effect of learning modality on engagement [20].

### Student interpersonal skills and student emotion regulation skills reciprocally predict one another

Theoretical frameworks and empirical evidence indicate that social-emotional skills (Student engaging with others and Student emotion regulation) do not exist in isolation but are bidirectionally related.

On the one hand, effective emotion regulation serves as a foundation for successful social interactions. When students can effectively manage their emotions—such as anger, anxiety, and frustration—they are more likely to exhibit calm, rational, and prosocial behaviors in interpersonal contexts, thereby establishing and maintaining positive peer relationships [21]. For instance, students who can regulate feelings of frustration are more likely to remain patient and avoid conflicts during collaborative learning, which directly predicts positive peer interactions. Conversely, deficits in emotion regulation may result in impulsive or withdrawn behaviors, hindering the formation of healthy relationships. Improvements in social-emotional skills, including emotion regulation, are significantly associated with increased peer connectedness, indicating that improved emotional management facilitates students’ acceptance and sense of connection within peer groups [22].

On the other hand, positive social interaction experiences provide a crucial learning environment and practical opportunities for the development of emotion regulation skills. Through positive interactions with peers, students learn to recognize and understand the emotional states of others—a process integral to perspective-taking, which is a key skill in engaging with others. This, in turn, helps them better reflect upon and regulate their own emotions [23]. The enhancement of social-emotional skills, including relational skills, is associated with increased peer connectedness, whereas the loss of friends or a decline in relationship quality—representing negative social interactions—can significantly negatively impact well-being. This underscores the feedback effect of social interaction outcomes on emotional states. Consequently, a supportive peer environment rich in positive interactions functions as a “practice field”, allowing students to exercise and internalize emotion regulation strategies through lived experience.

### Social emotional skills (student engaging with others) predict student development

In the academic domain, positive peer relationships are regarded as a significant supportive resource. Classmate connectedness is positively associated with students’ academic engagement by fulfilling their needs for autonomy, relatedness, and competence [24]. When students feel accepted and supported by their peers, they are more inclined to engage in learning activities and demonstrate higher levels of learning motivation and participation.

In the context of psychological well-being and social adjustment, the role of positive peer interaction skills becomes even more pronounced. Close relationships with peers and teachers are significantly associated with higher life satisfaction, self-esteem, positive affect, and lower levels of internalizing behavior problems such as depression and anxiety [25]. Students who are capable of establishing and maintaining high-quality friendships generally exhibit greater school well-being and better mental health [26]. For students with high baseline levels of peer connectedness, a decline in such connectedness significantly predicts a decrease in well-being, strongly underscoring the protective role of sustained positive social interactions for psychological health.

### Social emotional skills (student emotion regulation) predict student development

Student emotion regulation skills are key predictors of positive development. Effective emotion regulation enables students to better cope with academic pressure and social challenges, thereby fostering positive outcomes in both cognitive and affective domains.

In the academic context, emotion regulation skills are associated with students’ learning processes and outcomes. Perceived emotion regulation ability, that is, confidence in one’s own capacity to regulate emotions, is significantly associated with more positive homework practices, stronger expressive skills, and enhanced perspective-taking skills. Students who can manage academic anxiety and frustration are able to engage in learning tasks with greater concentration and employ more effective learning strategies, ultimately enhancing their academic achievement [27]. The generation of positive academic emotions relies on effective emotion regulation, and these positive academic emotions play a mediating role in the process through which teacher support relates to academic engagement.

At the level of mental health and personal development, emotion regulation skills play a crucial role. They represent a core competency for students to cope with stress and maintain psychological balance. Emotion regulation ability is closely associated with students’ adaptive behaviors [21]. The development of emotion regulation ability is a central objective of social emotional learning (SEL) programs [26]. Social emotional learning (SEL) programs can reduce students’ internalizing and externalizing behavior problems [28] and enhance their overall well-being. The inability to regulate emotions effectively is often associated with internalizing problems such as anxiety and depression, as well as externalizing problems such as impulsivity and aggression [29]. In contrast, successful emotion management is linked to higher life satisfaction and more frequent positive emotional experiences [25].

## Materials and methods

### Theoretical framework

Grounded in Social Cognitive Theory, this study examines how school connectedness is associated with positive student development through the mediating role of social-emotional skills. Social Cognitive Theory emphasizes the triadic reciprocal determinism among individual, behavior, and environmental factors, providing a solid theoretical foundation for the causal pathway model constructed in this research. From this theoretical perspective, school connectedness is regarded as a key environmental factor, while students’ social-emotional skills represent crucial individual cognitive and behavioral regulatory capacities. The interaction between these elements contributes to students’ developmental outcomes within the school context [30].

Specifically, this research framework first posits that school connectedness (environment) significantly is associated with students’ social-emotional skills (individual/behavior). This pathway aligns with the emphasis in Social Cognitive Theory on how the environment shapes individual cognition and behavior. When students perceive a sense of belonging, respect, and support from their school, they are more likely to develop the confidence and motivation to cultivate positive social interaction skills [31]. Subsequently, students’ interpersonal skills are hypothesized to be positively associated with positive development and emotion regulationl skills. This reflects the interaction between the individual and behavioral dimensions within Social Cognitive Theory, as proficient social interactions provide essential social contexts and practical opportunities for individuals to learn to identify, understand, and manage their own emotions [32]. Ultimately, these two core social-emotional skills, as key agents of individual agency, are hypothesized to jointly contribute to students’ positive development—including well-being, positive school emotions, and school engagement. This pathway clearly illustrates the relationship between individual behavior and developmental outcomes, as emphasized in Social Cognitive Theory [33,34].

In summary, the research framework, grounded in Social Cognitive Theory, systematically elucidates the underlying mechanism through which environmental support from the school facilitates the acquisition of individual social-emotional skills, ultimately leading to comprehensive positive development. This provides a clear conceptual guide and analytical basis for subsequent variable measurement, path analysis, and interpretation of results.

### Variable and data

This study is a secondary analysis of de-identified, publicly available data from the OECD Survey on Social and Emotional Skills (SSES). The original data collection followed relevant ethical guidelines and obtained informed consent from participants (or their legal guardians). The present analysis was conducted in accordance with the OECD data use terms and does not require additional ethical approval.

Social-emotional skills (student engaging with others), which is student interpersonal skills, include energy, assertiveness and sociability. Energy refers to an individual’s tendency to exhibit high levels of vitality and active engagement in daily life, such as voluntarily participating in physical activities or classroom discussions. Assertiveness reflects the capacity to articulate one’s own viewpoints and defend personal rights in social or academic contexts—for instance, taking the lead in task allocation during group work or voicing disagreement explicitly. Sociability describes the ability to proactively establish and maintain interpersonal relationships, including being inclined to meet new people and engage effectively in team collaboration [1].

Social-emotional skills (student emotion regulation) include stress resistance, optimism and emotional control. Stress resistance refers to the capacity to maintain psychological stability and avoid excessive anxiety when confronted with academic pressure or setbacks, such as remaining calm in examination situations. Optimism reflects a tendency to hold positive expectations about future outcomes, such as believing that effort will lead to success. Emotional control involves the ability to manage negative emotions effectively, preventing emotionally driven behaviors from impairing decision-making [1].

Student school connectedness is a multidimensional construct, the core of which encompasses student classroom relationships, student-teacher relationships, and teacher feedback. The quality of students’ relationships with their peers is a key component of school connectedness, with positive peer interactions significantly enhancing students’ sense of belonging and emotional engagement [22]. The perceived relationship with teachers is particularly crucial; warm teacher-student interactions make students feel respected and supported, thereby strengthening their identification with the school. Teacher feedback, as a direct manifestation of teacher-student interaction, plays a significant predictive role in students’ sense of belonging: positive feedback helps students perceive teachers’ care, which is associated with their school connectedness [35]. These three dimensions are interrelated and collectively constitute students’ experience of belonging in school. For instance, positive peer and teacher-student relationships provide emotional support, while teacher feedback further reinforces this connectedness by fulfilling students’ needs for autonomy and competence [21]. Therefore, school connectedness relies not only on the quality of social relationships but also on sustained emotional and cognitive support within the educational environment.

Student positive development is a multidimensional construct, centered on three key domains: student well-being, positive emotions at school, and student school engagement. Student well-being involves not only affective and cognitive evaluations of school life but is also closely associated with positive teacher-student interactions, peer acceptance, and school connectedness. It serves as a vital indicator of an individual’s overall psychological health and life satisfaction within the school context [26]. Positive emotions at school, such as joy, pleasure, and pride, are related with students’ learning motivation, creative thinking, and ability to concentrate. Emotional support from teachers has been shown to effectively contribute to students’ positive academic emotions, thereby enriching their affective experiences during learning [23]. Student school engagement encompasses behavioral, cognitive, and affective dimensions, with higher levels of engagement being strongly associated with improved academic achievement and psychosocial adjustment. School connectedness, particularly through supportive teacher-student relationships and peer support, has been identified as a key protective factor in predicting student engagement levels. In summary, these three domains are interrelated and mutually reinforcing, collectively forming the core of student positive development and providing clear direction for educational practice and intervention [36].

This study conducted data analysis using SPSS and MPlus software. The dataset (SSES R2 ST PUF INTERNATIONAL) contained 65,613 observations. After focusing on items and regional samples relevant to this study, 23,560 observations were retained. Unrelated data were removed using the listwise deletion method. Among the retained observations and variables included in this study, there were no missing data. The core analytical variables in this study were computed as continuous scale scores derived from original ordered categorical items. These scores were generated based on item response theory (IRT) and were therefore treated as continuous variables in the analyses. This study conducted a normality test to examine whether the quantitative data followed a normal distribution. Given the large sample size, the Kolmogorov-Smirnov (K-S) test was used. The results showed significant values for all variables, indicating that the data did not conform to a normal distribution. However, normality assumptions are often stringent and difficult to meet in practice. In this study, the absolute values of most kurtosis were less than 10. Only the kurtosis value for ASS_WLE_ADJ was 11.240, which, nonetheless, remained well below the liberal threshold (|kurtosis| < 20). The kurtosis values for all other variables fell within reasonable ranges. Accordingly, the data can be considered to exhibit acceptable univariate normality, supporting the use of maximum likelihood estimation. Absolute values of skewness were less than 3, suggesting that although the data were not perfectly normal, they were generally acceptable as approximately normally distributed (See Table 1).

**Table 1:** Results of the Normality Test (Kolmogorov–Smirnov Test).

	Mean	SD	Skewness	Kurtosis	K-S D statistic	*p*
**STR_WLE_ADJ**	529.806	95.540	0.735	4.943	0.072	0.000**
**OPT_WLE_ADJ**	567.422	96.560	0.581	1.435	0.060	0.000**
**EMO_WLE_ADJ**	538.946	107.487	0.687	3.605	0.083	0.000**
**SOC_WLE_ADJ**	557.435	96.428	0.697	3.333	0.065	0.000**
**ASS_WLE_ADJ**	539.760	98.355	1.102	11.240	0.097	0.000**
**ENE_WLE_ADJ**	537.860	102.446	0.934	5.148	0.069	0.000**
**ST_RELTEACH**	51.774	10.551	0.626	0.308	0.186	0.000**
**ST_FEEDBACK**	53.739	10.239	−0.003	−0.163	0.137	0.000**
**ST_STUCLASS**	52.845	11.166	0.767	1.014	0.193	0.000**
**ST_SCHENGAM**	51.993	10.986	0.069	0.443	0.102	0.000**
**ST_POSEMOT**	51.452	11.195	0.182	0.727	0.104	0.000**
**ST_WELLBEING**	51.885	10.744	0.137	0.810	0.106	0.000**

To examine the robustness of the mediating mechanism, this study conducted 5,000 bootstrap resampling tests after controlling for city and gender. The model converged normally, and all 5,000 bootstrap iterations were successfully completed. The results showed that the indirect effect of school connectedness on student development through the ability of engaging with others was significant, Estimate = 0.391, SE = 0.022, 95% BC bootstrap CI [0.350, 0.438]. The serial mediating effect of school connectedness on student development through both the ability of engaging with others and emotion regulation was also significant, Estimate = 0.109, SE = 0.017, 95% BC bootstrap CI [0.074, 0.141]. The total indirect effect was also significant, Estimate = 0.500, SE = 0.011, 95% BC bootstrap CI [0.479, 0.520]. Given that none of the above confidence intervals contained zero, the mediating mechanism remained valid and robust after controlling for site and gender.

The analyses were conducted at the scale-score level, specifically using the MS variables (Student Weight and Scores) derived from the official item-level data. As such, this study represents a more in-depth exploration and synthesis beyond the officially reported results.

The total sample size was 23,560, comprising participants from five Asian cities: Delhi (India), Dubai (UAE), Gunma (Japan), Jinan (China), and Kudus (Indonesia). Among these cities, Kudus contributed the largest number of participants, with a sample size of 6,400. Within the overall sample, 11,772 students were male, accounting for 49.97% of the total (see Table 2).

**Table 2:** Results of frequency analysis.

Item	Option	Frequency	Ratio (%)
**Site**	DEL	2193	9.31
	DUB	5282	22.42
	GUN	3320	14.09
	JIN	6365	27.02
	KUN	6400	27.16
**Gender**	1.0	11772	49.97
	2.0	11788	50.03
**Account**		23560	100.0

## Result

As shown in Table 3, the Cronbach’s alpha coefficient is 0.795, which comprehensively indicates that the data reliability is high. KMO value is 0.861, which is higher than 0.8, indicating that the research data suit for extracting information. Therefore, the processed data have high reliability and validity. The reliability indicators for each dimension also meet the standards. The standardized Cronbach’s alpha coefficients are 0.773, 0.763, 0.706, and 0.847.

**Table 3:** Reliability analysis.

Item	CITC	Deleted items α	Cronbach αof each construct	Cronbach α
**STR_WLE_ADJ**	0.637	0.656	0.770	0.795
**OPT_WLE_ADJ**	0.598	0.698		
**EMO_WLE_ADJ**	0.583	0.721		
**SOC_WLE_ADJ**	0.611	0.662	0.762	
**ASS_WLE_ADJ**	0.566	0.712		
**ENE_WLE_ADJ**	0.605	0.669		
**ST_RELTEACH**	0.639	0.470	0.707	
**ST_FEEDBACK**	0.389	0.771		
**ST_STUCLASS**	0.563	0.568		
**ST_SCHENGAM**	0.812	0.694	0.848	
**ST_POSEMOT**	0.842	0.661		
**ST_WELLBEING**	0.524	0.958		

The constructs are defined based on the SSES framework; therefore the confirmatory factor analysis (CFA) is employed. This analysis focuses on four factors and twelve analytical items. As known in Table 4, the effective sample size for this analysis is 23,560, which is more than ten times the number of analytical items, indicating a moderate sample size. The factor loading coefficient values reflect the correlation between factors (latent variables) and analytical items (observed variables/measurement items). The standardized loading coefficient values represent the correlation between factors and analytical items (measurement items). All items exhibit significance, and the standardized loading coefficient values are greater than 0.4, indicating a strong correlation.

**Table 4:** Factor loading coefficient table.

Factor	Observed variable	Coef.	Std. Error	*z* (CR)	*p*	Std. Estimate	SMC
**School connectedness**	**ST_RELTEACH**	1.000	–	–	–	0.783	0.614
	**ST_FEEDBACK**	0.579	0.009	64.130	0.00	0.467	0.218
	**ST_STUCLASS**	1.067	0.011	96.084	0.00	0.789	0.623
**Engaging with others**	**SOC_WLE_ADJ**	1.000	–	–	–	0.691	0.477
	**ASS_WLE_ADJ**	0.974	0.011	85.889	0.00	0.679	0.461
	**ENE_WLE_ADJ**	1.175	0.013	90.627	0.00	0.734	0.539
**Emotion regulation**	**STR_WLE_ADJ**	1.000	–	–	–	0.716	0.512
	**OPT_WLE_ADJ**	1.054	0.011	96.224	0.00	0.737	0.544
	**EMO_WLE_ADJ**	1.023	0.011	91.011	0.00	0.687	0.472
**Student development**	**ST_SCHENGAM**	1.000	–	–	–	0.927	0.860
	**ST_POSEMOT**	1.088	0.004	250.981	0.00	0.990	0.981
	**ST_WELLBEING**	0.570	0.006	94.684	0.00	0.541	0.293

“-” indicates that the item is a reference item.

The model fit indices of the confirmatory factor analysis are in Table 5. When testing the measurement model, the results of the confirmatory factor analysis indicate that most model fits are acceptable.

**Table 5:** Measurement model fitting index.

Common indicators	GFI	CFI	NFI	NNFI	TLI	IFI
**Value**	0.917	0.930	0.929	0.903	0.903	0.930
**Common indicators**	**PGFI**	**PNFI**	**PCFI**	**SRMR**	**RMSEA**	**χ2/df**
**Value**	0.564	0.676	0.676	0.081	0.108	275

As shown in Table 6, although the Average Variance Extracted (AVE) values for some constructs fell slightly below the recommended threshold of 0.5 (0.485 and 0.492, respectively) in the assessment of convergent validity, the Composite Reliability (CR) for all constructs exceeded the suggested criterion of 0.7. According to Fornell and Larcker [37], convergent validity can still be considered adequate when CR values meet the standard, even if AVE is marginally below 0.5. Furthermore, all item factor loadings were statistically significant at the p < 0.001 level, providing additional support for the convergent validity of the scales.

**Table 6:** Results of Model AVE and CR Indicators.

Factor	AVE	CR
**School connectedness**	0.485	0.729
**Engaging with others**	0.492	0.744
**Emotion regulation**	0.509	0.757
**Student development**	0.711	0.875

Confirmatory factor analysis (CFA) can be used to assess discriminant validity. The diagonal values in Table 7 represent the square roots of AVE, while the remaining values represent the correlation coefficients. The square roots of AVE reflect the degree of convergence within each factor, whereas the correlation coefficients represent the relationships among factors. If the square root of a factor’s AVE is greater than the absolute value of its correlation coefficient with other factors, discriminant validity is supported. As shown in Table 7, this criterion was met for all factors. Therefore, all factors demonstrated good discriminant validity.

**Table 7:** Discriminant validity: Pearson correlations and square roots of AVE.

	School connectedness	Engaging with others	Emotion regulation	Student development
**School connectedness**	0.696			
**Engaging with others**	0.430	0.702		
**Emotion regulation**	0.473	0.588	0.714	
**Student development**	0.505	0.521	0.549	0.843

In Table 8, school connectedness relates to student positive development (including student well-being, positive emotions at school and student school engagement) through student social-emotional skills (engaging with others & emotion regulation). School connectedness predicts social-emotional skills (student engaging with others). Social-emotional skills (student engaging with others) predicts social-emotional skills (student emotion regulation). Social-emotional skills (student engaging with others) predicts student positive development. Student emotion regulation skills is associated with student positive development.

**Table 8:** Summary table of regression coefficients.

X	→	Y	B	*SE*	*z* (CR)	*p*	β
**School connectedness**	**→**	**Engaging with others**	4.866	0.072	67.615	0.000**	0.782
**Engaging with others**	**→**	**Emotion regulation**	1.198	0.018	65.993	0.000**	0.709
**Engaging with others**	**→**	**Student development**	0.096	0.002	38.842	0.000**	0.468
**Emotion regulation**	**→**	**Student development**	0.023	0.001	17.171	0.000**	0.187
**School connectedness**	**→**	**ST_STUCLASS**	1.110	0.012	95.433	0.000**	0.787
**School connectedness**	**→**	**ST_FEEDBACK**	0.602	0.010	63.202	0.000**	0.466
**School connectedness**	**→**	**ST_RELTEACH**	1.000	–	–	–	0.750
**Engaging with others**	**→**	**ENE_WLE_ADJ**	1.505	0.020	76.764	0.000**	0.718
**Engaging with others**	**→**	**ASS_WLE_ADJ**	1.223	0.017	71.813	0.000**	0.640
**Engaging with others**	**→**	**SOC_WLE_ADJ**	1.000	–	–	–	0.578
**Emotion regulation**	**→**	**EMO_WLE_ADJ**	0.900	0.009	95.387	0.000**	0.695
**Emotion regulation**	**→**	**OPT_WLE_ADJ**	0.908	0.009	100.843	0.000**	0.749
**Emotion regulation**	**→**	**STR_WLE_ADJ**	1.000	–	–	–	0.773
**Student development**	**→**	**ST_WELLBEING**	0.570	0.006	93.710	0.000**	0.537
**Student development**	**→**	**ST_POSEMOT**	1.090	0.004	244.375	0.000**	0.991
**Student development**	**→**	**ST_SCHENGAM**	1.000	–	–	–	0.926

“→” indicates a regression or measurement relationship

“-” indicates that the item is a reference category

**Fig 1:**
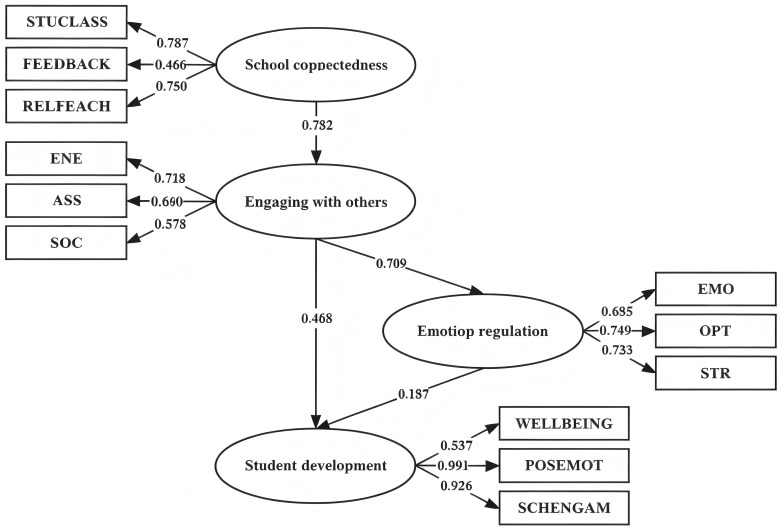
SEM result.

Here is the SEM pathway (Fig 1).

School connectedness is positively associated with Social-emotional skills (student engaging with others), β = 0.782. Social-emotional skills (student engaging with others) is positively associated with Social-emotional skills (student emotion regulation), β = 0.709. Social-emotional skills (student engaging with others) is positively associated with student positive development, β = 0.468.

Social emotional skills (student emotion regulation) is positively associated with student positive development, β = 0.187.

In School connectedness, Student-Classmate relationship (β = 0.787) and Perceived relationship with teachers (β = 0.750) are more effective. In student engaging with others, Energy is most effective, β = 0.718. In Social-emotional skills (student emotion regulation), Stress Resistance (β = 0.773) and Optimism (β = 0.749) are more effective. In Student positive development, Positive emotions at school is most effective, β = 0.991. Student school engagement is also important, β = 0.926.

The model fit is acceptable. The model fitting indicators are as follows. In this study, CFI = 0.904 > 0.9, NFI = 0.904 > 0.9, IFI = 0.904 > 0.9, PGFI = 0.570 > 0.5, PNFI = 0.685 > 0.5, PCFI = 0.685 > 0.5. Goodness-of-fit indices should typically exceed 0.9 [38]. Indices exceeding 0.50 serve as the minimum acceptable threshold for models, representing the most fundamental baseline. When indices meet or exceed 0.50, the model demonstrates basic explanatory power. These findings indicate that this study satisfies the standards for acceptable fit [39–41]. The incremental fit indices (CFI, NFI, IFI) met the minimum criteria, indicating a significant improvement of the proposed model over the baseline model. The parsimony fit indices (PGFI, PNFI, PCFI) were all above 0.50, which is generally considered acceptable. These indices penalize model complexity and suggest that the model achieves a reasonable balance between complexity and goodness-of-fit.

In this study, χ²/df = 358. Under conditions of a very large sample size, the chi-square test is almost always significant, and the chi-square-to-degrees-of-freedom ratio becomes excessively inflated; consequently, the model fit based on the chi-square test is typically rejected. Fit indices such as the RMSEA and TLI are substantially influenced by model complexity, sample size, and the distributional characteristics of the observed variables. Given the extremely large sample size and the relatively large number of observed indicators in the model, the RMSEA in this study was 0.123. Although slightly above conventional thresholds, this value is considered acceptable, and its 90% confidence interval [0.110, 0.129] indicates a relatively stable estimate. The TLI was 0.874, approaching the common benchmark of 0.90, and is deemed acceptable given the complexity of the model. The SRMR was 0.109, slightly above the recommended threshold of 0.10. The SRMR reflects the average discrepancy between the model-implied covariance matrix and the sample covariance matrix. In models involving a large number of observed variables (12 in this study) and complex path relationships, a slight elevation in the SRMR is a common phenomenon. An SRMR value below 0.10 is generally considered acceptable, while values between 0.10 and 0.12 can be regarded as marginally acceptable. Given that several fit indices of the structural equation model reached only acceptable levels, the model demonstrated marginal to moderate fit, falling short of an optimal level and merely meeting the threshold for acceptability. Therefore, while the analysis of the significance and directional consistency of the path coefficients can be considered highly reliable, the interpretation of effect sizes for each path should be approached with caution and appropriate flexibility, and should be positioned as exploratory findings. Comparisons regarding the magnitude of effects across different paths warrant further refinement through model modification or validation using independent samples.

Furthermore, in this study, all hypothesized paths were statistically significant (p < 0.001), with standardized path coefficients ranging from 0.187 to 0.782, consistent with theoretical expectations. The R²values for the endogenous latent variables were as follows: Engaging with others (0.611), Emotion regulation (0.503), and School performance (0.379), indicating that the model exhibited moderate to strong explanatory power for the core variables.

Model evaluation adhered to the principle of holistic judgment. Although some fit indices in this study did not reach the optimal levels suggested by conventional benchmarks, the overall model demonstrated acceptable fit given the practical constraints of an extremely large sample size and a complex measurement structure. The combination of indices reported in this study constitutes a mutually reinforcing and logically consistent evidence chain, collectively indicating that the model fit is statistically acceptable. The basic adequacy of the model is thereby confirmed, supporting the subsequent path analysis (see Table 9).

**Table 9:** Structural model fitting index.

Common indicators	CFI	NFI	IFI	PGFI	PNFI	PCFI
**Value**	0.904	0.904	0.904	0.570	0.685	0.685

Based on this, this study included students’ city of residence and gender as control variables in the structural equation model. After scaling the observed variables to reduce differences in variable scales, the structural equation model with site and gender as control variables converged normally. The results showed that all core structural paths remained significant. To improve numerical stability, the observed indicators were rescaled before SEM estimation. WLE-based indicators were divided by 100, and student scale indicators were divided by 10. Standardized coefficients were used for interpretation.

The data for this study were collected from five cities: Delhi (India), Dubai (UAE), Gunma (Japan), Jinan (China), and Kudus (Indonesia), with Delhi (India, DEL) serving as the reference category. When the control variable is a five-level categorical variable, including four dummy variables allows for complete group comparisons, with the omitted category automatically serving as the reference group. Its effect is absorbed into the model intercept and therefore does not appear in the path coefficient estimates. Accordingly, the model included four categorical control variables for the cities (DUB, GUN, JIN, KUD) and one demographic control variable for gender (gender_Std). The MLR estimator was used to examine the effects on paths among latent variables. The variance inflation factor (VIF) for all control variables was below 3, indicating no serious multicollinearity concerns.

Regarding the effects of control variables on Engaging with others, the standardized effect for DUB was 0.044, indicating that respondents in this group exhibited relatively higher levels of Engaging with others. The effect for GUN was −0.221 (p < 0.001), representing the strongest negative influence among the control variables, suggesting that students from Gunma had significantly lower levels of Engaging with others. The effect for JIN was not statistically significant (β = 0.005, p = 0.589). The effect for KUD was 0.019 (p = 0.033), indicating a weak positive influence. The standardized effect for gender was 0.092 (p < 0.001), indicating that male students exhibited higher levels of Engaging with others compared to female students.

In terms of effects on Emotion regulation, the effect for DUB was not significant (β = −0.008, p = 0.371). GUN showed a significant positive effect (β = 0.148), indicating that students from Gunma had higher levels of Emotion regulation. The effects for JIN (β = 0.072) and KUD (β = 0.115) both positively predicted Emotion regulation. Gender also demonstrated a positive effect (β = 0.093, p < 0.001), with male students showing relatively higher levels of Emotion regulation.

Regarding effects on Student development, the effect for DUB was 0.038 (p < 0.001), indicating a weak positive influence. The effect for GUN was 0.027 (p = 0.006), representing a relatively weak positive influence. The effect for JIN was not statistically significant (β = 0.003, p = 0.797). The positive effect for KUD was the most pronounced (β = 0.174, p < 0.001), making it the strongest control variable influencing Student development, suggesting that students from Kudus demonstrated better developmental outcomes. The effect for gender was 0.013 (p = 0.026), indicating only a weak predictive role.

In summary, the results of the control variables indicate regional differences. Using Delhi as the reference group, students from Gunma showed significantly lower levels of Engaging with others, whereas students from Kudus demonstrated the most prominent performance in Student development. Students from Jinan exhibited no significant differences from the reference group across any dimension. Students from Dubai showed slightly higher levels only in the Engaging with others dimension compared to the reference group. At the demographic level, male students exhibited a weak but statistically significant advantage across all dimensions. Overall, the differential impact of geographical and cultural backgrounds on student development was more pronounced than that of gender.

## Discussion

This study found that school connectedness predicts the development of students’ social-emotional skills, specifically engaging with others. Within this construct, student-classmate relationships and perceived relationships with teachers emerged as particularly critical influencing factors [42]. This finding aligns closely with the existing theoretical framework, indicating that school connectedness, as a positive psychological resource, fulfills students’ need for belonging and provides a secure and supportive environment for the practice and enhancement of their social-emotional skills [43].

Specifically, positive peer relationships provide students with a direct context to practice social skills, such as cooperation, empathy, and conflict resolution, and high-quality peer interactions effectively predict development of SES [27]. Perceived positive teacher-student relationships, on the other hand, through teachers’ role modeling, emotional support, and timely feedback, not only directly strengthen students’ school connectedness but also offer key scaffolding at cognitive and emotional levels for students to acquire and apply social-emotional skills [14,35,44]. Together, these two relational dimensions constitute the core of school connectedness. Their underlying mechanism may lie in the fact that they collectively reduce students’ anxiety in social situations and improve their willingness and confidence to engage in social interactions and emotional expression [20,43].

Therefore, educational practice should move beyond treating school connectedness as a unitary construct and instead prioritize the cultivation of high-quality student-student and teacher-student interactions within the school environment. By establishing supportive relational networks, schools can more effectively translate a sense of belonging into concrete drivers that predict students’ social-emotional development [12,45].

In the domain of social-emotional skills, students’ interpersonal skills (engaging with others) positively relates to their emotion regulation abilities, and these two skill sets collectively contribute to positive student development—including well-being, positive school emotions, and academic engagement. Social-emotional skills constitute an interconnected system, in which forming positive connections with others—such as peer and teacher-student relationships—provides students with a crucial social support network [24]. This supportive environment facilitates students’ ability to better recognize, understand, and manage their own emotions. When students feel accepted and respected by peers and teachers, their need for belonging is fulfilled, which establishes a psychological foundation for effective emotion regulation [21]. Furthermore, positive interpersonal interactions serve as practical contexts for exercising emotion regulation; through these interactions, students learn to negotiate, empathize, and resolve conflicts—processes that directly train their emotion management capabilities [22]. Therefore, enhancing students’ interpersonal skills represents a key pathway to fostering their emotion regulation capacity.

This study, framed within Social Cognitive Theory, aims to elucidate how school connectedness relates to positive student development through the mediating pathway of social-emotional skills. The findings are highly consistent with the theoretical hypotheses and, on this basis, concretize and deepen the application of the theory in this specific context.

The results provide robust support for the core tenet of Social Cognitive Theory regarding the dynamic and reciprocal interaction among personal, behavioral, and environmental factors [23]. Specifically, this study validated the chain mediation pathway of “school connectedness (environment)→interpersonal skills (person/behavior)→emotional regulation skills (person/behavior)→positive development (outcome)”. This clearly illustrates the “triadic reciprocal determinism” emphasized by Social Cognitive Theory: a positive school environment (positive teacher-student and peer relationships), as a key environmental factor, can significantly foster the development of individuals’ internal cognitive and behavioral capabilities (social initiative, energy) [4]. These enhanced personal capabilities (interpersonal skills) can, in turn, shape subsequent behavioral regulation capacities (emotion management) [46]. Ultimately, these two core sets of social-emotional skills, as manifestations of personal agency, jointly contribute to positive developmental outcomes, such as academic engagement and positive affect. This complete pathway model empirically demonstrates the dynamic process whereby environmental support relates to positive behavioral outcomes by stimulating and shaping a sequence of internal personal skills, serving as a compelling illustration of Social Cognitive Theory within the school context [24].

Furthermore, the specific findings of this study enrich and refine the application of Social Cognitive Theory in school settings. While the theory posits that personal, behavioral, and environmental factors relate to each other, this research, through empirical analysis, reveals that this relationship, within the school domain, specifically follows a sequential and hierarchical development of skills. The study particularly highlights that, among interpersonal skills, “energy” plays a particularly critical role; and among emotional regulation skills, “stress resistance” and “optimism” have more pronounced effects [47]. This provides a more nuanced perspective for understanding the specific constituents of the “personal” dimension and their differential roles within the causal chain. Additionally, the study identifies “teacher-student relationships” and “peer relationships” as the most potent environmental elements constituting school connectedness that trigger the subsequent skill chain, thereby making the operational definition of the “environmental” factor more explicit [8]. Consequently, this research not only validates the core mechanisms of Social Cognitive Theory with empirical data but also, by revealing the specific sequence and key factors within skill development, provides richer and more operational details for the theory when explaining student developmental pathways. This deepens our understanding of how the school environment can foster students’ holistic development through the systematic cultivation of social-emotional skills.

At the level of specific skill dimensions, this study identifies differential effects. “Energy” plays a pivotal role among interpersonal skills. As a state characterized by vitality and enthusiasm, energy serves as the foundation for students to proactively initiate and sustain positive social interactions [48]. Students with high energy are more readily accepted by their peers, thereby establishing broader and higher-quality friendships, which in turn provides sustained momentum for their social-emotional development [22]. Within emotion regulation skills, “Stress Resistance” and “Optimism” demonstrate stronger effects. Stress resistance enables students to effectively cope with academic and social setbacks, preventing them from falling into cycles of negative emotion [21]. Optimism, as a positive explanatory style, helps students perceive challenges as temporary and surmountable, which not only contributes to emotional stability but is also directly linked to higher levels of school well-being and engagement. The perception of emotional competencies—including stress resistance and optimism—emerges as the most consistent predictor of positive academic practices and social-emotional skills [47].

Among the outcome variables of positive development, positive emotions at school and school engagement are particularly prominent. Positive emotions expand students’ thought-action repertoires and build enduring personal resources, such as increased interest in learning and exploratory behaviors. Students who experience more positive emotions at school demonstrate greater cognitive flexibility, creativity, and resilience, which in turn facilitates deeper engagement in learning [6]. School engagement, as a behavioral manifestation of positive development, directly reflects students’ sustained involvement and effort in the academic process, serving as a key indicator of academic success and personal fulfillment [24]. The positive realtionship between teacher SES and student engagement, although with a small effect size, confirms its significance [36].

From an Asian perspective, education systems across the region generally emphasize collectivism and relational harmony. Against this backdrop, peer relationships and teacher-student relationships not only serve as core components of school belonging but also function as the “relational soil” for the development of students’ social-emotional skills. “Student-peer relationships” and “perceived teacher-student relationships” contribute most significantly to school belonging, which aligns closely with Asian cultural traditions that value interpersonal bonds and respect for authority (e.g., respect for teachers and moral education) [49]. Asian students’ sense of school belonging relies more on high-quality interpersonal interactions than on mere institutional affiliation [50].

Asian students generally face high levels of academic pressure [51]. Among emotion regulation skills, “stress tolerance” and “optimism” are particularly prominent in predicting positive development. This finding may reflect the “adaptive function” of emotion regulation skills within the Asian cultural context. Students require not only general emotional control abilities but also specific skills to cope with pressure and maintain positive expectations in highly competitive and evaluative educational environments [52].

Because Asian cultures encourage self-regulation and interpersonal restraint within the collective, students first obtain group identification and emotional support through positive interpersonal interactions (e.g., cooperation, empathy), which then become internalized as individual-level emotion management abilities [53]. This contrasts with the Western individualistic approach, where emotion regulation is often cultivated as an independent individual skill. Therefore, the model proposed in this study is not only statistically valid but also culturally interpretable [54].

The facilitating effect of school belonging on social-emotional skills is not a universal linear process; rather, it presents a culturally specific chain-mediated pathway within the Asian educational ecology characterized by collectivism, high academic pressure, and interpersonal orientation.

Urban differences can be cross-interpreted through dimensions such as cultural value variations, educational system orientations, and social support structures. The significantly lower performance of students in Gunma, Japan, on “Engaging with others” may be related to the “high-context restraint” characteristic of East Asian collectivist culture. Social norms in Japanese society emphasize individual integration into the collective rather than proactively expanding interpersonal connections, leading students to prefer maintaining distance and avoiding excessive self-disclosure in interactions. Such cultural traits tend to suppress measured scores on “actively interacting with others and participating in group activities” [55]. In contrast, cities such as Dubai in the United Arab Emirates, serving as multicultural immigration hubs, exhibit greater tolerance for open and extroverted interactions, which naturally enhances students’ willingness to engage interpersonally [56].

The outstanding performance of students in Kudus, Indonesia, on “Student development” can be attributed to the strong buffering effect of its community support network. Indonesia is a typical society characterized by high collectivism and strong religious ties. Non-major cities such as Kudus retain close-knit clan and community mutual aid traditions, where students’ academic pressure and psychological distress are quickly buffered by families and communities. Additionally, as local education is in an ascending phase, policies tend to strongly support the development of disadvantaged groups, enabling students to exhibit “catch-up advantages” in comprehensive development indicators such as academic achievement and mental health [57]. In contrast, Jinan, China, as a typical East Asian educational hub, experiences high competitive pressure among students that offsets some of the resource dividends, resulting in no significant difference from the reference group.

Regarding gender differences, the weak but consistent advantage of male students aligns with the bias characteristics of developmental stages and measurement tools in adolescence. The measurement of “Emotion regulation” in this study is largely based on “problem-solving-oriented emotional coping”. Although adolescent males exhibit higher impulsivity than females, they tend to report higher self-efficacy in the use of instrumental emotion management strategies [58].

In summary, this study underscores a chain-reaction model: by fostering students’ interpersonal skills—particularly energy—their emotion regulation capacities, notably stress resistance and optimism, can be enhanced. This fulfills their basic psychological needs, leading to a significant increase in positive emotions at school and school engagement, and ultimately promoting their holistic positive development.

## Conclusion

This study, using structural equation modeling, validates the internal mechanism through which school connectedness predicts student positive development via the chain-mediating pathway of “engaging with others → emotion regulation” in social-emotional skills. School connectedness, particularly student-classmate relationship and perceived relationship with teachers, is positively associated students’ interpersonal skills (such as energy). These skills, in turn, predicts the development of emotion regulation skills (such as stress resistance and optimism). Together, these competencies contribute to students’ positive emotions at school and school engagement, ultimately facilitating positive development. The research reveals the hierarchical and sequential nature of social-emotional skill development, providing a precise practical pathway for schools to promote holistic student development by optimizing relational environments and implementing sequential cultivation of social-emotional skills.

In conclusion, based on Asian country samples, this study validates the sequential mediating role of interpersonal skills (studnet engaging with others) and emotional regulation skills in the process by which school connectedness positively relates to student development. The findings explicitly indicate that within the Asian educational and cultural context, enhancing campus connectedness—centered on peer relationships and teacher-student relationships—can effectively cultivate a sequential progression of students’ social-emotional skills. Specifically, this pathway stimulates vitality in interpersonal interactions, which in turn fosters resilience and optimism in coping with stress, ultimately leading to a significant enhancement of students’ positive emotions at school and academic engagement. This conclusion deepens the understanding of the “school environment—personal skills—developmental outcomes” mechanism within the Asian context, providing a targeted foundation for implementing educational practices based on the sequential development of social-emotional skills in this cultural region.

### Implication and Limitation

This study elucidates the specific mediating mechanisms through which school connectedness is associated with students’ positive development. While existing research has established the positive role of school connectedness in student development, the underlying pathways remain insufficiently explored. The present paper demonstrates through empirical analysis that school connectedness does not directly lead to student positive development—such as well-being, positive emotions at school, and learning engagement—but rather operates by enhancing two key categories of SES: interpersonal skills (e.g., proactivity, social competence, and energy) and emotion regulation skills (e.g., stress resistance, emotional control, and optimism).

This study further clarifies the intrinsic relationship and sequential effect between interpersonal skills and emotion regulation skills. The findings indicate that interpersonal skills is positively related with the development of emotion regulation skills. This suggests that within the school context, students’ engagement in positive interactions with others, such as effective communication and cooperation, can enhance their capacity for emotion management. An OECD report similarly notes that students with stronger social competencies tend to exhibit greater stress resistance and a more optimistic outlook. Through structured path analysis, this paper establishes interpersonal skills as an antecedent to emotion regulation skills, thereby deepening the understanding of the dynamic interrelationships within SES.

This study provides precise targets for educational intervention. The findings suggest that enhancing school connectedness can serve as an effective entry point for promoting SES, with priority attention to fostering students’ interpersonal skills, which in turn facilitates the holistic development of their emotion regulation abilities. Furthermore, the results align with the OECD report’s conclusion that “emotion regulation skills significantly predict well-being and academic achievement”, thereby offering a theoretical foundation for schools to design tiered and sequential strategies for social-emotional education. By revealing the mechanism through which school connectedness realtes to student development via the dual-pathway of social-emotional skills, this study addresses a gap in process-oriented research on the “school environment→skill development→positive outcomes” sequence and provides actionable theoretical support for educational practice.

The implications of this study suggest that when striving to enhance school belongingness and cultivate students’ social-emotional skills, educational practices in Asian regions should take full account of the local cultural context. For instance, leveraging the Asian cultural emphasis on collectivism and relationships, activities that promote teamwork and deep teacher-student interaction can be designed to strengthen school belongingness as the foundational starting point. Regarding the sequence of skill cultivation, priority may be given to developing interpersonal skills (e.g., vitality, cooperation), which aligns with the value Asian societies place on harmonious relationships and can lay the social foundation for subsequent emotional regulation skill development. Meanwhile, the cultivation of stress management and optimism should be designed with targeted consideration of the common academic challenges faced by Asian students.

This study has several limitations. Common method bias represents a prevalent potential issue in cross-sectional self-report studies, which may arise from common rater effects, measurement context, item context, or scale format characteristics. At the theoretical level, this bias could potentially homogenously inflate the correlations among all variables, thereby possibly amplifying the magnitude of the path coefficients observed in this study. Consequently, caution is warranted when interpreting the absolute strength of these path coefficients, suggesting that they should be regarded as “theoretical upper bounds” of the true effect sizes. However, common method bias does not undermine the core theoretical model and hypotheses of this study. The primary contribution of this research lies in testing the existence and relative strength of the theoretical mechanism whereby school connectedness relates to positive development through a multiple mediation chain of social-emotional skills. Even in the presence of common method variance, its impact is likely to be uniformly distributed across different paths within the model. Under this assumption, comparisons of the relative importance among variables and the overall goodness of fit of the model may be less affected by such bias compared to the absolute magnitude of individual path coefficients. The main conclusion of this study, that school connectedness is associated with student development through cultivating specific social-emotional skills, relies precisely on this structured pattern of path relationships, rather than merely on high correlations. To enhance the persuasiveness of the findings, preventative statistical controls were implemented during the data analysis phase, including ensuring satisfactory discriminant validity of the scales through confirmatory factor analysis and presenting detailed model fit indices in the results reporting.

The cross-sectional data preclude definitive conclusions about causality, allowing only for the examination of associations. The reported fit indices collectively indicate statistically acceptable model fit for subsequent SEM analysis, though not perfect fit. This represents a limitation of the current study, and future research should provide more comprehensive fit evidence.

Some observed variables exhibited excessively high factor loadings on their corresponding latent constructs, suggesting potential construct redundancy or conceptual overlap despite adequate overall model fit. The high factor loading of Student positive emotion (close to 1.0) may indicate potential construct redundancy or substantial conceptual overlap with the latent variable of student positive development. Although school engagement and student well-being were both included in the study, student positive emotion almost entirely represents the overall latent construct of student positive development. This phenomenon may be attributed to the strong theoretical interrelatedness of this indicator with the other two dimensions (particularly school engagement), or it may suggest that, given the current measurement tools and sample, the three dimensions are empirically difficult to distinguish, thereby compromising the discriminant validity of the higher-order construct. Therefore, when interpreting the path effects of student positive development in this study, one should avoid equating it simply with a broad, multidimensional developmental state. Instead, it is more appropriate to understand it as a locally composite indicator dominated by student positive emotion, while also incorporating elements of academic engagement behaviors and basic well-being. Future research should validate this higher order construct through improved instruments, discriminant validity testing, or complex model specifications (e.g., allowing residual correlations) to ensure result robustness.

Although the present study included city and gender as control variables, further refinement is needed in accounting for the hierarchical structure inherent in the student sample, in which students are naturally nested within different city and school contexts. Such hierarchical structures may lead to non-independence among observations, thereby affecting the accuracy of parameter estimates and the reliability of standard errors. Future research could address this by conducting multigroup analysis to test model invariance across regions, or employing hierarchical linear models to correct for clustering effects. Cross cultural comparative designs could also clarify the moderating role of cultural values in the proposed pathways.
