# Supplementary material for: Exploring the impact path of school connectedness on student development through cultivating students’ social-emotional skills
Source: PLoS One. 2026 Jul 22;21(7):e0353972. doi: 10.1371/journal.pone.0353972 (PMC13390845; doi:10.1371/journal.pone.0353972)
Supplement: Attachment — Submitted filename: Review of revised manuscript.pdf [file pone.0353972.s003.pdf]

Review of revised manuscript (PONE-D-25-65886R1)

The revised manuscript addresses an important and timely research question using a large international dataset (OECD SSES 2023). The authors have made several improvements in response to the first round of review, particularly regarding data availability, clarification of ethical aspects, and the inclusion of additional measurement indices (e.g., CR, AVE, discriminant validity).

However, despite these revisions, several substantial methodological and reporting issues remain insufficiently addressed, particularly with regard to statistical rigor and the interpretation of SEM results. These issues limit the extent to which the manuscript can currently be considered technically sound.

Below are the main points that still require attention.

Major comments

1. Model fit reporting and interpretation (pp. 34–36)
   Although additional fit indices have been included, some key indicators are still missing, in particular:

- RMSEA (with confidence interval)
- Chi-square statistic and degrees of freedom

The manuscript continues to justify model adequacy based on a selective set of indices, stating that a "holistic judgment" is sufficient. This interpretation remains problematic. Standard SEM practice requires complete and transparent reporting of model fit indices, and conclusions should be based on the overall pattern of indices rather than partial compliance.

In addition, the reported values (e.g., CFI ≈ 0.90; TLI ≈ 0.90; SRMR = 0.081) indicate an acceptable but not strong fit, which should be acknowledged more explicitly rather than presented as fully satisfactory.

2. Treatment of the hierarchical data structure (p. 30; Table 1)
   The dataset includes students nested within different contexts (five cities); however:

- No control variables for site are included
- No multigroup or multilevel analyses have been conducted
- No correction for clustering has been applied

This issue was already raised in the first round of review and is now mentioned only as a limitation. However, given the size and structure of the dataset, it remains a methodologically relevant omission.

At a minimum, the authors should:

- include site (and, if available, other covariates such as gender or socioeconomic status) as control variables
  or
- provide a stronger methodological justification for not modeling the nested structure
  or
- at least indicate, in the future directions section, alternative analytical approaches and the use of statistical techniques that account for the hierarchical nature of the data

3. Extremely high factor loadings (pp. 35–36)
   Some loadings remain extremely high (e.g., student development → positive emotions $\beta \approx 0.99$). The manuscript acknowledges this as a limitation, which is appropriate.

However, the implications are not fully developed. Values of this magnitude suggest:

- possible construct redundancy
- limited discriminant validity at the higher-order level

A more cautious interpretation of the "student development" construct is needed, with a more explicit discussion of the potential overlap among indicators.

4. Use of EFA with theoretically defined constructs (pp. 31–33)
   The manuscript now includes both EFA and CFA. However, the rationale for conducting EFA remains weak, considering that:

- the constructs are defined a priori (OECD framework)
- a confirmatory approach would be expected

The current justification (EFA followed by CFA) does not fully resolve the initial concern. The authors should either:

- provide a clearer methodological rationale for this choice
  or
- emphasize the confirmatory framework more consistently

5. Conceptual framing and consistency (Introduction and Discussion)
   The cultural dimension (Asian context), introduced in the Introduction, remains insufficiently developed in the Discussion.

6. Causal language
   There has been some improvement in reducing causal language (e.g., increased use of "predicts" and "is associated with"). However, some formulations still suggest directional or causal interpretations that are not fully supported by the cross-sectional design.

Further refinement toward strictly associational language is recommended.

Minor comments

- The manuscript is clearer than in the previous version, but language and style still require revision, particularly to reduce repetition and improve fluency.
- Some sections remain overly verbose or redundant, especially in the Results.

Overall evaluation

The manuscript has improved in several respects, particularly in terms of transparency (data availability, ethical clarification) and partial strengthening of the measurement model. However, for the reasons outlined above, further revisions are still required.
