# Supplementary material for: Exploring the impact path of school connectedness on student development through cultivating students’ social-emotional skills
Source: PLoS One. 2026 Jul 22;21(7):e0353972. doi: 10.1371/journal.pone.0353972 (PMC13390845; doi:10.1371/journal.pone.0353972)
Supplement: Attachment — Submitted filename: Response to Reviewers.docx [file pone.0353972.s004.docx]

PONE-D-25-65886

Exploring the impact path of school connectedness on student development through cultivating students' social-emotional skills

PLOS One

Dear Dr. Wang,

Thank you for submitting your manuscript to PLOS ONE. After careful consideration, we feel that it has merit but does not fully meet PLOS ONE’s publication criteria as it currently stands. Therefore, we invite you to submit a revised version of the manuscript that addresses the points raised during the review process.

PONE-D-25-65886

PLOS One

Please submit your revised manuscript by Apr 11 2026 11:59PM. If you will need more time than this to complete your revisions, please reply to this message or contact the journal office at plosone@plos.org. When you're ready to submit your revision, log on to <https://www.editorialmanager.com/pone/>

and select the 'Submissions Needing Revision' folder to locate your manuscript file.

Please include the following items when submitting your revised manuscript:

A letter that responds to each point raised by the academic editor and reviewer(s). You should upload this letter as a separate file labeled 'Response to Reviewers'.

A marked-up copy of your manuscript that highlights changes made to the original version. You should upload this as a separate file labeled 'Revised Manuscript with Track Changes'.

An unmarked version of your revised paper without tracked changes. You should upload this as a separate file labeled 'Manuscript'.

If you would like to make changes to your financial disclosure, please include your updated statement in your cover letter. Guidelines for resubmitting your figure files are available below the reviewer comments at the end of this letter.

If applicable, we recommend that you deposit your laboratory protocols in protocols.io to enhance the reproducibility of your results. Protocols.io assigns your protocol its own identifier (DOI) so that it can be cited independently in the future. For instructions see: <https://journals.plos.org/plosone/s/submission-guidelines#loc-laboratory-protocols>

Additionally, PLOS ONE offers an option for publishing peer-reviewed Lab Protocol articles, which describe protocols hosted on protocols.io. Read more information on sharing protocols at <https://plos.org/protocols?utm_medium=editorial-email&utm_source=authorletters&utm_campaign=protocols.>

The data used has been stored on the figshare public platform

The link is 10.6084/m9.figshare.31832035

We look forward to receiving your revised manuscript.

Kind regards,

Carlotta Rivella

Guest Editor

PLOS One

Journal Requirements:

When submitting your revision, we need you to address these additional requirements.

1.Please ensure that your manuscript meets PLOS ONE's style requirements, including those for file naming. The PLOS ONE style templates can be found at

<https://journals.plos.org/plosone/s/file?id=wjVg/PLOSOne_formatting_sample_main_body.pdf>

and

<https://journals.plos.org/plosone/s/file?id=ba62/PLOSOne_formatting_sample_title_authors_affiliations.pdf>

The manuscript has been revised to conform to the journal’s formatting requirements.

2. In the online submission form you indicate that your data is not available for proprietary reasons and have provided a contact point for accessing this data. Please note that your current contact point is a co-author on this manuscript. According to our Data Policy, the contact point must not be an author on the manuscript and must be an institutional contact, ideally not an individual. Please revise your data statement to a non-author institutional point of contact, such as a data access or ethics committee, and send this to us via return email. Please also include contact information for the third party organization, and please include the full citation of where the data can be found.

Revised as requested.

3. Please amend either the title on the online submission form (via Edit Submission) or the title in the manuscript so that they are identical.

Revised as requested.

4. Please ensure that you refer to Figure 1 in your text as, if accepted, production will need this reference to link the reader to the figure.

Revised as requested.

5. We note you have included a table to which you do not refer in the text of your manuscript. Please ensure that you refer to Table 1-5 in your text; if accepted, production will need this reference to link the reader to the Table.

Revised as requested.

6. If the reviewer comments include a recommendation to cite specific previously published works, please review and evaluate these publications to determine whether they are relevant and should be cited. There is no requirement to cite these works unless the editor has indicated otherwise.

Revised as requested.

Additional Editor Comments:

Dear Authors,

after carefully reading your manuscript and considering the reviewers’ comments, we recognize the potential value of your work. However, there are substantial methodological and statistical limitations that currently prevent the manuscript from being suitable for publication.

Accordingly, we are requesting a major revision. We strongly encourage you to carefully address the reviewers’ suggestions, as implementing these revisions will significantly improve the quality and rigor of your manuscript.

When submitting your revised manuscript, please include a detailed response letter that outlines how each reviewer and editor comment has been addressed. If you decide not to follow a particular suggestion, provide a clear justification for your choice.

Carlotta Rivella

Reviewers' comments:

Reviewer's Responses to Questions

Comments to the Author

1. Is the manuscript technically sound, and do the data support the conclusions?

The manuscript must describe a technically sound piece of scientific research with data that supports the conclusions. Experiments must have been conducted rigorously, with appropriate controls, replication, and sample sizes. The conclusions must be drawn appropriately based on the data presented.

Reviewer #1: Partly

Reviewer #2: Partly

2. Has the statistical analysis been performed appropriately and rigorously?

Reviewer #1: No

Reviewer #2: No

3. Have the authors made all data underlying the findings in their manuscript fully available?

The PLOS Data policy requires authors to make all data underlying the findings described in their manuscript fully available without restriction, with rare exception (please refer to the Data Availability Statement in the manuscript PDF file). The data should be provided as part of the manuscript or its supporting information, or deposited to a public repository. For example, in addition to summary statistics, the data points behind means, medians and variance measures should be available. If there are restrictions on publicly sharing data—e.g. participant privacy or use of data from a third party—those must be specified.

Reviewer #1: No

Reviewer #2: Yes

4. Is the manuscript presented in an intelligible fashion and written in standard English?

PLOS ONE does not copyedit accepted manuscripts, so the language in submitted articles must be clear, correct, and unambiguous. Any typographical or grammatical errors should be corrected at revision, so please note any specific errors here.

Reviewer #1: No

Reviewer #2: Yes

5. Review Comments to the Author

Please use the space provided to explain your answers to the questions above. You may also include additional comments for the author, including concerns about dual publication, research ethics, or publication ethics. (Please upload your review as an attachment if it exceeds 20,000 characters)

Reviewer #1: The manuscript addresses a relevant research question using a large international dataset (OECD SSES 2023, N = 23,560) and applies structural equation modeling to test a sequential mediation model. The topic is timely and potentially suitable for PLOS ONE. However, several issues must be addressed before the study can be considered technically sound and compliant with journal standards.

First, the statistical analysis, while generally appropriate in terms of method selection, is not reported with sufficient rigor. The measurement model requires stronger validation. Reliability is reported only once (Cronbach’s alpha = 0.795), without specifying to which construct it refers. Reliability indices should be provided separately for each latent construct. Additionally, Composite Reliability (CR), Average Variance Extracted (AVE), and evidence of discriminant validity are not reported. Given that the constructs are theoretically predefined based on OECD SSES, the rationale for conducting an exploratory factor analysis (EFA) instead of a confirmatory factor analysis (CFA) should be clarified and justified.

Based on exploratory factor analysis, this study supplements with confirmatory factor analysis (CFA). This analysis focuses on four factors and twelve analytical items for CFA. The effective sample size for this analysis is 23,560, which is more than ten times the number of analytical items, indicating a moderate sample size. The factor loading coefficient values reflect the correlation between factors (latent variables) and analytical items (observed variables/measurement items). The standardized loading coefficient values represent the correlation between factors and analytical items (measurement items). All items exhibit significance, and the standardized loading coefficient values are greater than 0.4, indicating a strong correlation (See Table 5).

| Table 5. Factor loading coefficient table | | | | | | | |
| --- | --- | --- | --- | --- | --- | --- | --- |
| Factor | Observed variable | Coef. | Std. Error | *z* (CR) | *p* | Std. Estimate | SMC |
| School connectedness | ST_RELTEACH | 1.000 | - | - | - | 0.783 | 0.614 |
|  | ST_FEEDBACK | 0.579 | 0.009 | 64.130 | 0.000 | 0.467 | 0.218 |
|  | ST_STUCLASS | 1.067 | 0.011 | 96.084 | 0.000 | 0.789 | 0.623 |
| Engaging with others | SOC_WLE_ADJ | 1.000 | - | - | - | 0.691 | 0.477 |
|  | ASS_WLE_ADJ | 0.974 | 0.011 | 85.889 | 0.000 | 0.679 | 0.461 |
|  | ENE_WLE_ADJ | 1.175 | 0.013 | 90.627 | 0.000 | 0.734 | 0.539 |
| Emotion regulation | STR_WLE_ADJ | 1.000 | - | - | - | 0.716 | 0.512 |
|  | OPT_WLE_ADJ | 1.054 | 0.011 | 96.224 | 0.000 | 0.737 | 0.544 |
|  | EMO_WLE_ADJ | 1.023 | 0.011 | 91.011 | 0.000 | 0.687 | 0.472 |
| Student development | ST_SCHENGAM | 1.000 | - | - | - | 0.927 | 0.860 |
|  | ST_POSEMOT | 1.088 | 0.004 | 250.981 | 0.000 | 0.990 | 0.981 |
|  | ST_WELLBEING | 0.570 | 0.006 | 94.684 | 0.000 | 0.541 | 0.293 |
| Note: The dash “-” indicates that the item is a reference item. | | | | | | | |

Model fit reporting is incomplete. Important indices such as RMSEA, TLI, and chi-square statistics are not presented. The interpretation of model fit is overly permissive (e.g., stating that it is sufficient for only some indices to meet thresholds). This should be revised in line with standard SEM reporting practices.

The model fit indices of the confirmatory factor analysis are as follows. When testing the measurement model, the results of the confirmatory factor analysis indicate that the model fits well, and all indices meet the recommended standards (See Table 6).

| Table 6. Measurement model fitting index | | | | | | | | | | |
| --- | --- | --- | --- | --- | --- | --- | --- | --- | --- | --- |
| Common indicators | GFI | CFI | NFI | NNFI | TLI | IFI | PGFI | PNFI | PCFI | SRMR |
| Judgment criteria | >0.9 | >0.9 | >0.9 | >0.9 | >0.9 | >0.9 | >0.5 | >0.5 | >0.5 | <0.1 |
| Value | 0.917 | 0.930 | 0.929 | 0.903 | 0.903 | 0.930 | 0.564 | 0.676 | 0.676 | 0.081 |

Some standardized coefficients are extremely high (e.g., loadings approaching or exceeding 0.99), suggesting potential construct overlap or redundancy. These values require closer examination and discussion.

In the present study model, certain observed variables exhibited excessively high factor loadings on their corresponding latent constructs. For instance, the factor loadings of positive affect in school and student school engagement on positive student development approached 0.99. This may indicate insufficient statistical discrimination between these measurement indicators and the latent variable, suggesting potential construct redundancy or conceptual overlap. Although the overall model fit was satisfactory, these extremely high loadings warrant a cautious approach to interpreting the measurement validity of the core construct "positive student development." Future research should further validate the structural validity of this higher order latent construct through improved measurement instruments (such as incorporating more differentiated indicators), testing discriminant validity, or employing more complex model specifications (such as allowing residual correlations) to ensure the robustness of the research conclusions.

Second, the Data Availability Statement does not comply with PLOS ONE policy. Although the submission indicates that all data are available without restriction, the manuscript states that data can be obtained by emailing the authors. Since the OECD SSES dataset is publicly available, the authors should provide the official repository link or DOI and clearly state that the data are publicly accessible without restriction.

Revised as requested.

10.6084/m9.figshare.31832035

Third, the Ethics Statement is currently listed as “N/A.” Even though the study uses secondary, anonymized data, the manuscript should clarify that the data are fully de-identified and publicly available, and explain why additional ethical approval was not required.

Revised as requested.

Fourth, the manuscript contains multiple grammatical and stylistic issues (e.g., phrasing such as “There are may fit indices”) and repetitive structures. Because PLOS ONE does not provide copyediting after acceptance, careful language revision is necessary before resubmission.

Revised as requested.

Finally, the study is based on cross-sectional data, yet causal language (e.g., “promotes,” “fosters”) is used throughout. Interpretations should be reframed in terms of statistical associations rather than causal effects.

Revised as requested.

In summary, while the dataset and overall modeling approach have potential, substantial revisions are required to meet the technical, reporting, and editorial standards of the journal.

Reviewer #2: INTRODUCTION

The theoretical framework is presented within the Method section rather than being clearly articulated in the Introduction.

Revised as requested.

The Introduction is comparatively long, somewhat redundant, and insufficiently supported by relevant citations. A more concise structure and a stronger integration of the existing literature would improve clarity and strengthen the study.

Existing research has largely examined the pairwise relationships and constituent elements among school connectedness, students' social emotional skills, and student development. However, studies integrating the tripartite relationships and holistic pathways remain relatively scarce.

First, research on the impact of school connectedness on student development indicates that school connectedness enhances students' social emotional skills, such as social competence and emotional regulation, thereby promoting positive developmental outcomes like academic engagement and well being. These findings align with theoretical frameworks such as self determination theory (Huttunen et al., 2025). Teacher student relationships and peer relationships are core components of school connectedness, playing complex mediating roles between emotional regulation and academic engagement. School connectedness and positive peer relationships have been identified as crucial environmental foundations for the development of students' social emotional skills (e.g., interpersonal skills, emotional regulation), which in turn foster positive student development (Schwab et al., 2022).

Second, in studies concerning the school environment (e.g., peer relationships, teacher student relationships) and students' social emotional skills, particularly emotional regulation and interpersonal skills, teacher student and peer relationships serve as key contextual factors influencing student development, including positive emotions, academic engagement, and learning motivation (Ge, 2025). Teacher student relationships, especially closeness and teacher support, are central to enhancing students' academic engagement, well being, and prosocial behavior (Articles 33, 37, 39). Teachers' own social emotional competencies (e.g., mentalization, emotional regulation, mindfulness) are considered critical mechanisms that directly affect the quality of teacher student interactions and the development of students' skills (Cecere et al., 2025). Emotional regulation skills act as a core mediating mechanism linking social support to positive developmental outcomes, such as academic engagement, emotional resilience, and learning motivation (Zheng et al., 2025). Additionally, self regulated learning, reflective practice, personalized feedback, and learner agency play significant roles in optimizing learning experiences and emotional outcomes (Kuklick, 2025).

In summary, existing theoretical research supports the constituent pathways of a chain mediation model following the sequence of "supportive school environment to interpersonal/social emotional skills (particularly emotional regulation) to positive developmental outcomes." However, empirical validation of the complete pathway using objective data remains an area requiring further innovative exploration.

Although the focus on the Asian educational context is explicitly mentioned in the Introduction, this cultural perspective is not revisited in the remainder of the manuscript. The cultural dimension should be explicitly addressed again in the Discussion, Conclusion, Implications and Limitations section.

Discussion

This study, conducted within an Asian educational context, reveals that school belongingness promotes positive student development through a sequential mediating pathway involving social emotional skills, specifically progressing from interpersonal skills to emotional regulation skills. This finding warrants interpretation within the specific Asian cultural context. Educational systems in Asia generally emphasize collectivism, respect for teachers, and academic achievement, which may render student student relationships and teacher student relationships particularly crucial in fostering school belongingness. These relationships provide students with core social contexts for practicing cooperation, respect, and mutual support. Concurrently, the academic pressure faced by Asian students may accentuate the importance of two specific emotional regulation skills, namely stress resistance and optimism, as key competencies for maintaining positive school related emotions and academic engagement under high pressure circumstances. Furthermore, Asian cultural norms may encourage restraint and harmony, potentially shaping the developmental pathways of emotional expression and regulation strategies. Thus, the pathway model identified in this study reflects how specific Asian cultural values and social expectations shape the interrelationships among school environment, skill development, and positive developmental outcomes.

Conclusion

In conclusion, based on a sample from five Asian cities, this study validates the sequential mediating role of interpersonal skills and emotional regulation skills in the process by which school belongingness influences positive student development. The findings explicitly indicate that within the Asian educational and cultural context, enhancing campus connectedness centered on student student relationships and teacher student relationships can effectively cultivate a sequential progression of students' social emotional skills. Specifically, this pathway stimulates vitality in interpersonal interactions, which in turn fosters resilience and optimism in coping with stress, ultimately leading to a significant enhancement of students' positive emotions at school and academic engagement. This conclusion deepens the understanding of the school environment to personal skills to developmental outcomes mechanism within the Asian context, providing a targeted foundation for implementing educational practices based on the sequential development of social emotional skills in this cultural region.

Implications and Limitations

The implications of this study suggest that when striving to enhance school belongingness and cultivate students' social emotional skills, educational practices in Asian regions should take full account of the local cultural context. For instance, leveraging the Asian cultural emphasis on collectivism and relationships, activities that promote teamwork and deep teacher student interaction can be designed to strengthen school belongingness as the foundational starting point. Regarding the sequence of skill cultivation, priority may be given to developing interpersonal skills such as vitality and cooperation, which aligns with the value Asian societies place on harmonious relationships and can lay the social foundation for subsequent emotional regulation skill development. Meanwhile, the cultivation of stress management and optimism should be designed with targeted consideration of the common academic challenges faced by Asian students.

A major limitation of this study concerns its cultural generalizability. The data were collected from only five Asian cities, and the applicability of the conclusions across different subcultures within Asia, as well as their generalizability to non Asian cultural backgrounds such as individualistic cultures in Europe and America, requires further examination. Future research could employ cross cultural comparative designs to clarify the moderating role of cultural values in the proposed influence pathways.

METHOD AND RESULTS

Preliminary analyses are not reported and should be explicitly included to strengthen the methodological transparency of the study. In particular, the manuscript would benefit from reporting:

• Reliability indices for each dimension (in addition to any global alpha coefficient);

The reliability indicators for each dimension also meet the standards (See Table 2).

| Table 2. Reliability analysis | | | | |
| --- | --- | --- | --- | --- |
| Item | CITC | Deleted items α | Cronbach αof each construct | Cronbach α |
| STR_WLE_ADJ | 0.637 | 0.656 | 0.770 | 0.795 |
| OPT_WLE_ADJ | 0.598 | 0.698 |  |  |
| EMO_WLE_ADJ | 0.583 | 0.721 |  |  |
| SOC_WLE_ADJ | 0.611 | 0.662 | 0.762 |  |
| ASS_WLE_ADJ | 0.566 | 0.712 |  |  |
| ENE_WLE_ADJ | 0.605 | 0.669 |  |  |
| ST_RELTEACH | 0.639 | 0.470 | 0.707 |  |
| ST_FEEDBACK | 0.389 | 0.771 |  |  |
| ST_STUCLASS | 0.563 | 0.568 |  |  |
| ST_SCHENGAM | 0.812 | 0.694 | 0.848 |  |
| ST_POSEMOT | 0.842 | 0.661 |  |  |
| ST_WELLBEING | 0.524 | 0.958 |  |  |
| Note: The standardized Cronbach's alpha coefficients are 0.773, 0.763, 0.706, and 0.847 | | | | |

• A transparent description of the handling of missing data;

This study employed listwise deletion to address missing data in the dataset. Specifically, prior to conducting the primary analyses (e.g., structural equation modeling), we removed all participant cases with missing values on any of the core study variables (i.e., school connectedness, interpersonal skills, emotion regulation skills, positive development and its dimensional measures, as well as all control variables).

• A clear justification of the estimator used (e.g., ML, MLR, or WLSMV), including its appropriateness given the measurement scale of the items;

The core analytical variables in this study were continuous scale scores calculated based on original ordinal categorical items, specifically composite scale scores derived from item response theory (IRT). Consequently, these variables were treated as continuous in the analyses. Under this condition, maximum likelihood estimation with robust standard errors and chi-square statistics was employed. The model fit indices reported in the Results section are commonly used evaluation criteria under ML estimation, indicating that the researchers adopted an estimation framework appropriate for continuous variables, which is consistent with the measurement scale properties of the analyzed variables.

• Information regarding normality assumptions and any robustness checks conducted;

This study employed structural equation modeling (SEM), a multivariate statistical method based on covariance structures, for which the commonly used maximum likelihood estimation typically assumes that the observed data are derived from a multivariate normal distribution. The present study assumed that the data approximately met the requirements for analysis.

• Effect sizes alongside statistical significance, especially considering the large sample size;

Revisions and discussions were conducted as required.

• A sensitivity analysis addressing the potential impact of self-report bias.

This study acknowledged the potential issue of self-report bias and listed it as a limitation.

Additional methodological clarifications are needed:

It remains unclear whether the analyses were conducted at the item level or at the scale-score level. The coding structure suggests the use of specific indicators, whereas the narrative refers to broader latent dimensions. This discrepancy should be clarified to ensure transparency and replicability.

The analyses conducted in this study were performed at the scale score level, examining the MS variables (Student Weight and Scores) derived from official specific items, representing further in-depth exploration and synthesis.

The use of Exploratory Factor Analysis (EFA) appears inconsistent with the presence of a clearly stated theoretical model. When a priori theoretical assumptions are available, Confirmatory Factor Analysis (CFA) would generally be more appropriate. The rationale for choosing EFA should therefore be justified.

Based on exploratory factor analysis, this study supplements with confirmatory factor analysis (CFA). This analysis focuses on four factors and twelve analytical items for CFA. The effective sample size for this analysis is 23,560, which is more than ten times the number of analytical items, indicating a moderate sample size. The factor loading coefficient values reflect the correlation between factors (latent variables) and analytical items (observed variables/measurement items). The standardized loading coefficient values represent the correlation between factors and analytical items (measurement items). All items exhibit significance, and the standardized loading coefficient values are greater than 0.4, indicating a strong correlation (See Table 5).

| Table 5. Factor loading coefficient table | | | | | | | |
| --- | --- | --- | --- | --- | --- | --- | --- |
| Factor | Observed variable | Coef. | Std. Error | *z* (CR) | *p* | Std. Estimate | SMC |
| School connectedness | ST_RELTEACH | 1.000 | - | - | - | 0.783 | 0.614 |
|  | ST_FEEDBACK | 0.579 | 0.009 | 64.130 | 0.000 | 0.467 | 0.218 |
|  | ST_STUCLASS | 1.067 | 0.011 | 96.084 | 0.000 | 0.789 | 0.623 |
| Engaging with others | SOC_WLE_ADJ | 1.000 | - | - | - | 0.691 | 0.477 |
|  | ASS_WLE_ADJ | 0.974 | 0.011 | 85.889 | 0.000 | 0.679 | 0.461 |
|  | ENE_WLE_ADJ | 1.175 | 0.013 | 90.627 | 0.000 | 0.734 | 0.539 |
| Emotion regulation | STR_WLE_ADJ | 1.000 | - | - | - | 0.716 | 0.512 |
|  | OPT_WLE_ADJ | 1.054 | 0.011 | 96.224 | 0.000 | 0.737 | 0.544 |
|  | EMO_WLE_ADJ | 1.023 | 0.011 | 91.011 | 0.000 | 0.687 | 0.472 |
| Student development | ST_SCHENGAM | 1.000 | - | - | - | 0.927 | 0.860 |
|  | ST_POSEMOT | 1.088 | 0.004 | 250.981 | 0.000 | 0.990 | 0.981 |
|  | ST_WELLBEING | 0.570 | 0.006 | 94.684 | 0.000 | 0.541 | 0.293 |
| Note: The dash “-” indicates that the item is a reference item. | | | | | | | |

The model fit indices of the confirmatory factor analysis are as follows. When testing the measurement model, the results of the confirmatory factor analysis indicate that the model fits well, and all indices meet the recommended standards (See Table 6).

| Table 6. Measurement model fitting index | | | | | | | | | | |
| --- | --- | --- | --- | --- | --- | --- | --- | --- | --- | --- |
| Common indicators | GFI | CFI | NFI | NNFI | TLI | IFI | PGFI | PNFI | PCFI | SRMR |
| Judgment criteria | >0.9 | >0.9 | >0.9 | >0.9 | >0.9 | >0.9 | >0.5 | >0.5 | >0.5 | <0.1 |
| Value | 0.917 | 0.930 | 0.929 | 0.903 | 0.903 | 0.930 | 0.564 | 0.676 | 0.676 | 0.081 |

The wellbeing factor shows cross-loadings on two scales with very similar loadings, raising concerns regarding discriminant validity and potential model misspecification. This issue requires further examination and discussion.

Under ideal conditions, exploratory factor analysis should yield high loadings for each measured variable on only one latent factor, thereby clearly delineating distinct theoretical constructs. The cross-loading of ENE_WLE_ADJ suggests that the trait of "energy" may not belong exclusively to the domain of skills for interacting with others, as originally hypothesized in the model. Theoretically, a high level of energy (manifested as vitality and active engagement) not only serves as a crucial driver of proactive social interactions (e.g., initiating conversations, participating in group activities) but may also represent an internal resource required for effective emotion regulation (e.g., coping with stress, maintaining positive affective states). Therefore, its significant loadings on two factors may reflect the foundational and cross-cutting role of energy within the system of social-emotional skills, suggesting that it potentially underpins both interpersonal engagement and emotional management processes. This does not invalidate the overall four-factor structure but indicates that the two higher-order constructs, namely "skills for interacting with others" and "emotion regulation skills," may exhibit some degree of conceptual overlap and interrelation in practice, rather than being entirely independent. In the discussion, it can be noted that this finding provides empirical insight into the interconnections among social-emotional skills, implying that certain core attributes (such as energy) may serve as a foundation for the co-development of multiple higher-order competencies.

According to the conventional practices and statistical principles of exploratory factor analysis (EFA), the classification of a variable with significant loadings on multiple factors is not arbitrary but follows clear guidelines. The core principle is to assign the variable to the factor on which it has the highest loading. In this study, the variable "ENE_WLE_ADJ" demonstrated a loading of 0.590 on Factor 3 ("social-emotional skills for engaging with others"), which was higher than its loading of 0.525 on Factor 1 ("social-emotional skills for emotion regulation"). Consequently, when interpreting and naming the factors, it should be classified as part of Factor 3. This indicates that the variable shares the greatest variance with the latent construct of "skills for interacting with others" and holds a stronger theoretical association, suggesting that "energy" is primarily utilized to explain students' activeness and initiative in social interactions.

Given the hierarchical structure of the data (students nested within schools, and schools nested within countries), multilevel analyses would be methodologically advisable. At a minimum, statistical controls for site, gender, age/grade, and socioeconomic status (if available) should be included to account for contextual variability.

The omission of the "region" factor constitutes a methodological limitation of this study. This decision was made because the study focused more on exploring the pathways of influencing factors at an overall macro-regional level, rather than examining differential effects within factors such as region. In similar multi-site international comparative studies, it is typically necessary to consider the potential impact of differences in culture, educational systems, or socioeconomic backgrounds on the relationships among variables. Neglecting this factor may affect the external validity of the findings, meaning that the results may not be equally generalizable to all five regions. Future research could build upon this study by incorporating region as a control variable, conducting multigroup analysis to test the invariance of the path model across different regions, or employing multilevel modeling to correct for clustering effects at the school or regional level, thereby enabling a more in-depth exploration of cross-cultural or cross-contextual universality and specificity.

Model fit indices appear relatively weak considering the large sample size. Although chi-square statistics are known to be highly sensitive in large samples, marginal or inadequate fit cannot be justified solely by noting that some indices meet recommended thresholds.

The incremental fit indices (CFI, NFI, IFI) met the minimum criteria, indicating that the model demonstrated a significant improvement over the baseline model. The parsimony fit indices (PGFI, PNFI, PCFI) were all greater than 0.50, which is generally considered acceptable. These indices, which penalize model complexity, suggested that the model achieved a reasonable balance between complexity and goodness of fit. The absolute fit index (SRMR) satisfied the commonly used criterion of being less than 0.08, indicating that the residuals of the model were relatively small. Model evaluation followed the principle of holistic judgment; the combination of indices reported in the text constituted a mutually corroborating and logically consistent chain of evidence, collectively demonstrating that the model fit was statistically acceptable. The basic plausibility of the model was confirmed, thereby supporting subsequent path analysis.

Furthermore, with large sample sizes, indices such as the chi-square test of model fit are typically rejected; therefore, this study relied on other fit indices and selectively reported certain indicators. Nevertheless, the existing combination of indices indicated that the model fit was statistically acceptable and could support subsequent structural equation modeling analyses, although it did not achieve perfect fit. This indeed represents a limitation of the current study, and future research should provide more comprehensive evidence of model fit.

Finally, the statistical significance of the factors reported in the table is not clearly indicated and should be explicitly provided.

Symbols for statistical significance were added.

DISCUSSION

The theoretical interpretation lacks consistency. Although the model is introduced as grounded in Social Cognitive Theory (SCT), the Discussion shifts toward an interpretation framed primarily in terms of Self-Determination Theory (SDT). This theoretical drift creates conceptual ambiguity and should be resolved.

This study, grounded in the framework of social cognitive theory, aimed to elucidate how school connectedness promotes positive development among students through the mediating pathway of social-emotional skills. The findings were highly consistent with the theoretical hypotheses and, on this basis, specified and deepened the application of the theory.

The results robustly supported the core tenet of social cognitive theory concerning the dynamic interplay among individual, behavior, and environment. Specifically, this study validated a chain mediation pathway: "school connectedness (environment) → engaging with others (individual/behavior) → emotion regulation skills (individual/behavior) → positive development (outcome)." This clearly illustrates the triadic reciprocal determinism emphasized by social cognitive theory: a positive school environment (e.g., positive teacher-student and peer relationships), serving as a key environmental factor, can significantly foster the development of individuals' internal cognitive and behavioral capacities (e.g., social initiative, vitality). These enhanced individual competencies (interpersonal skills) can, in turn, further influence and shape subsequent behavioral regulation capacities (emotion management). Ultimately, these two core categories of social-emotional skills, as manifestations of individual agency, jointly contribute to positive developmental outcomes, such as academic engagement and positive affect. This complete path model empirically demonstrates the dynamic process whereby environmental support, by activating and shaping a sequence of internal skills, drives positive behavioral outcomes, providing a compelling illustration of social cognitive theory within the educational context.

Furthermore, the specific findings of this study enrich and refine the application of social cognitive theory in school settings. While the theory posits reciprocal influences among individual, behavior, and environment, this study, through empirical analysis, revealed that within the school context, these influences specifically follow a sequential, hierarchical skill development pattern. Notably, the study identified that within interpersonal skills, "energy/vitality" plays a particularly critical role; within emotion regulation skills, the influence of "stress resistance" and "optimism" is more pronounced. This offers a more nuanced perspective for understanding the specific composition of the "individual" dimension and its differentiated roles within the causal chain. Moreover, the study clarified that "teacher-student relationships" and "peer relationships" are the most effective environmental elements constituting school connectedness, thereby triggering the subsequent skill chain, which renders the operational definition of the "environment" factor more explicit. Consequently, this research not only empirically validates the core mechanisms of social cognitive theory but also, by revealing the specific sequence and key components of skill development, provides richer and more actionable details for the theory in explaining student developmental pathways. This deepens our understanding of how the school environment can promote students' holistic development through the systematic cultivation of social-emotional skills.

Furthermore, the manuscript appears to imply causal relationships that are not warranted by the study design. Given the correlational nature of the data, the interpretations should be carefully reformulated to reflect associative rather than causal conclusions.

Revised as requested.

FORM AND WRITING STYLE

The manuscript is excessively schematized in several sections, which affects readability and academic tone. Some passages are redundant or insufficiently formal. For example, the statement:

“The model fitting indicators are as follows. There are may fit indices. It is difficult for all indicators to meet the standard, so as long as a portion of the indicators meet the standard is sufficient.”is problematic both linguistically and conceptually.

Revised as requested.

Beyond language concerns, the underlying methodological claim is questionable and requires a more rigorous justification.

Revised as requested.

FORM AND WRITING STYLE (minor)

The expression “social emotional skills” sometimes appears repetitive throughout the manuscript. Revising certain sentences to reduce redundancy would enhance overall readability.

Revised as requested.

DATA AVAILABILITY (minor)

Although the manuscript states that the data are accessible, providing a direct link to the dataset would substantially improve transparency and reproducibility.

Revised as requested.

The link to data is 10.6084/m9.figshare.31832035

6. PLOS authors have the option to publish the peer review history of their article (what does this mean?). If published, this will include your full peer review and any attached files.

If you choose “no”, your identity will remain anonymous but your review may still be made public.

Do you want your identity to be public for this peer review? For information about this choice, including consent withdrawal, please see our Privacy Policy.

Reviewer #1: No

Reviewer #2: Yes: Simone Pinetti

[NOTE: If reviewer comments were submitted as an attachment file, they will be attached to this email and accessible via the submission site. Please log into your account, locate the manuscript record, and check for the action link "View Attachments". If this link does not appear, there are no attachment files.]

To ensure your figures meet our technical requirements, please review our figure guidelines: <https://journals.plos.org/plosone/s/figures>

You may also use PLOS’s free figure tool, NAAS, to help you prepare publication quality figures: <https://journals.plos.org/plosone/s/figures#loc-tools-for-figure-preparation>

.

In compliance with data protection regulations, you may request that we remove your personal registration details at any time. (Remove my information/details). Please contact the publication office if you have any questions.

**Review Comments to the Author**

Thank you for the opportunity to review this manuscript. The paper addresses an important and timely question: how school connectedness relates to students’ positive development through specific social-emotional skills, using a large sample from the OECD SSES 2023 Asian dataset (N = 23,560). The topic is clearly relevant to contemporary educational research and fits within the scope of PLOS ONE, particularly given its emphasis on methodological soundness rather than perceived novelty.

Below, I provide detailed comments structured around the journal’s publication criteria and focused primarily on technical rigor, clarity, and compliance with editorial standards.

**1. Originality of the research**

The manuscript presents empirical analyses of a large international dataset and proposes a sequential mediation model (“school connectedness → engaging with others → emotion regulation → positive development”). This appears to constitute original research rather than a review or secondary analysis without added value.

That said, since the SSES 2023 dataset is publicly available and has already generated multiple reports and analyses, the manuscript would benefit from more clearly articulating what is analytically new. In particular:

- Has this specific sequential mediation model already been tested elsewhere with SSES 2023 data?
- In what way does this model advance beyond existing associations reported in OECD publications or recent empirical studies?

This specific sequential mediation model has not been tested elsewhere and represents an innovative model first proposed by this study.

The official OECD publications only provide analyses of the results from specific questionnaire items and the analysis of weighted relevant items. In contrast, this study conducted a more in-depth analysis utilizing the MS variables (Student Weight and Scores) provided in the official OECD publications. Specifically, these variables were treated as observed indicators to further summarize and synthesize new influencing factors. Therefore, this study represents a deeper exploration and dissection of existing resources. Compared with other empirical studies, this research employed structural equation modeling, which offers advantages over simple linear regression. The path coefficients in SEM are directional, allowing for a clear decomposition of the pathways through which influencing factors operate. Concurrently, the overall pathways in structural equation modeling are more complex than those in simple mediation models, which facilitates the inclusion of all factors in a comprehensive analysis and enables a全景展示 of the research findings.

The originality lies mainly in the proposed chain mediation structure, and making this contribution more explicit would strengthen the positioning of the study.

The primary original contribution of this study lies in empirically testing and revealing, through structural equation modeling, a clear sequential mediation pathway: "school connectedness → interpersonal skills → emotion regulation skills → positive development." Existing literature has largely focused on the direct association between school connectedness and development, or on single-dimensional mediating effects, lacking an integrated analysis of the synergistic effects of multidimensional social-emotional skills and their developmental sequence. This study not only verifies that school connectedness influences student development through the dual mediation of interpersonal skills and emotion regulation skills, but more importantly, it elucidates the sequential relationship between these two skills, namely, that enhanced interpersonal skills serve as an effective antecedent for the development of emotion regulation capacity. This finding clarifies the hierarchical and sequential nature of social-emotional skill development, providing a new theoretical basis and precise targets for designing school interventions based on the detailed process of "environment → skill sequence → developmental outcomes."

**2. Prior publication of results**

There are no obvious signs that the analyses have been published elsewhere. However, since the dataset is public and frequently analyzed, I recommend including a short clarification stating that:

- The structural model presented has not been previously published.
- The figures and tables are original.

Such transparency helps avoid ambiguity in secondary data analyses.

The structural model presented has not been previously published. This represents an innovative exploration based on publicly available data conducted in this study. The figures and tables are original.

**3. Technical and statistical standards**

This is the main area where revision is needed.

Measurement model and reliability

On page 15, a single Cronbach’s alpha (α = 0.795) is reported, but it is not clear to which construct this refers. Given that four latent constructs are modeled, reliability should be reported for each construct separately. Moreover:

- Composite Reliability (CR) and Average Variance Extracted (AVE) are not reported.
- Discriminant validity is not tested.
- An Exploratory Factor Analysis (EFA) is conducted, even though the constructs come from an established OECD framework.

In the assessment of convergent validity, although the Average Variance Extracted (AVE) values for some constructs fell slightly below the recommended threshold of 0.5 (0.485 and 0.492, respectively), the Composite Reliability (CR) for all constructs exceeded the suggested criterion of 0.7. According to Fornell and Larcker (1981), convergent validity can still be considered adequate when CR values meet the standard, even if AVE is marginally below 0.5. Furthermore, all item factor loadings were statistically significant at the p < 0.001 level, providing additional support for the convergent validity of the scales (See Table 7).

| Table 7. Results of Model AVE and CR Indicators | | |
| --- | --- | --- |
| Factor | AVE | CR |
| School connectedness | 0.485 | 0.729 |
| Engaging with others | 0.492 | 0.744 |
| Emotion regulation | 0.509 | 0.757 |
| Student development | 0.711 | 0.875 |

Fornell, C., & Larcker, D. F. (1981). Evaluating Structural Equation Models with Unobservable Variables and Measurement Error. Journal of Marketing Research, 18(1), 39–50. <https://doi.org/10.1177/002224378101800104>

Confirmatory factor analysis (CFA) can be used to differentiate validity studies. The diagonal line in the table represents the AVE square root value, while the remaining values represent the correlation coefficients. The AVE square root value can represent the aggregation of factors, and the correlation coefficient represents the correlation relationship. If the aggregation of factors is strong (significantly stronger than the absolute value of the correlation coefficient with other factors), it can indicate discriminant validity; If the square root of a factor's AVE is greater than the absolute value of its correlation coefficient with other factors. And if all factors present such a conclusion, it indicates good discriminant validity. Therefore, all factors have good discriminant validity (See Table 8).

| Table 8. Discriminant validity: Pearson correlation and AVE square root value | | | | |
| --- | --- | --- | --- | --- |
|  | School connectedness | Engaging with others | Emotion regulation | Student development |
| School connectedness | 0.696 |  |  |  |
| Engaging with others | 0.430 | 0.702 |  |  |
| Emotion regulation | 0.473 | 0.588 | 0.714 |  |
| Student development | 0.505 | 0.521 | 0.549 | 0.843 |

If the constructs are theory-driven and defined a priori, a Confirmatory Factor Analysis (CFA) would be more appropriate than EFA. At minimum, the choice of EFA should be justified.

Based on exploratory factor analysis, this study supplements with confirmatory factor analysis (CFA). This analysis focuses on four factors and twelve analytical items for CFA. The effective sample size for this analysis is 23,560, which is more than ten times the number of analytical items, indicating a moderate sample size. The factor loading coefficient values reflect the correlation between factors (latent variables) and analytical items (observed variables/measurement items). The standardized loading coefficient values represent the correlation between factors and analytical items (measurement items). All items exhibit significance, and the standardized loading coefficient values are greater than 0.4, indicating a strong correlation (See Table 5).

| Table 5. Factor loading coefficient table | | | | | | | |
| --- | --- | --- | --- | --- | --- | --- | --- |
| Factor | Observed variable | Coef. | Std. Error | *z* (CR) | *p* | Std. Estimate | SMC |
| School connectedness | ST_RELTEACH | 1.000 | - | - | - | 0.783 | 0.614 |
|  | ST_FEEDBACK | 0.579 | 0.009 | 64.130 | 0.000 | 0.467 | 0.218 |
|  | ST_STUCLASS | 1.067 | 0.011 | 96.084 | 0.000 | 0.789 | 0.623 |
| Engaging with others | SOC_WLE_ADJ | 1.000 | - | - | - | 0.691 | 0.477 |
|  | ASS_WLE_ADJ | 0.974 | 0.011 | 85.889 | 0.000 | 0.679 | 0.461 |
|  | ENE_WLE_ADJ | 1.175 | 0.013 | 90.627 | 0.000 | 0.734 | 0.539 |
| Emotion regulation | STR_WLE_ADJ | 1.000 | - | - | - | 0.716 | 0.512 |
|  | OPT_WLE_ADJ | 1.054 | 0.011 | 96.224 | 0.000 | 0.737 | 0.544 |
|  | EMO_WLE_ADJ | 1.023 | 0.011 | 91.011 | 0.000 | 0.687 | 0.472 |
| Student development | ST_SCHENGAM | 1.000 | - | - | - | 0.927 | 0.860 |
|  | ST_POSEMOT | 1.088 | 0.004 | 250.981 | 0.000 | 0.990 | 0.981 |
|  | ST_WELLBEING | 0.570 | 0.006 | 94.684 | 0.000 | 0.541 | 0.293 |
| Note: The dash “-” indicates that the item is a reference item. | | | | | | | |

The model fit indices of the confirmatory factor analysis are as follows. When testing the measurement model, the results of the confirmatory factor analysis indicate that the model fits well, and all indices meet the recommended standards (See Table 6).

| Table 6. Measurement model fitting index | | | | | | | | | | |
| --- | --- | --- | --- | --- | --- | --- | --- | --- | --- | --- |
| Common indicators | GFI | CFI | NFI | NNFI | TLI | IFI | PGFI | PNFI | PCFI | SRMR |
| Judgment criteria | >0.9 | >0.9 | >0.9 | >0.9 | >0.9 | >0.9 | >0.5 | >0.5 | >0.5 | <0.1 |
| Value | 0.917 | 0.930 | 0.929 | 0.903 | 0.903 | 0.930 | 0.564 | 0.676 | 0.676 | 0.081 |

Additionally, the description “the data reliability is high” seems overly strong based solely on a single alpha value.

The reliability indicators for each dimension also meet the standards (See Table 2).

| Table 2. Reliability analysis | | | | |
| --- | --- | --- | --- | --- |
| Item | CITC | Deleted items α | Cronbach αof each construct | Cronbach α |
| STR_WLE_ADJ | 0.637 | 0.656 | 0.770 | 0.795 |
| OPT_WLE_ADJ | 0.598 | 0.698 |  |  |
| EMO_WLE_ADJ | 0.583 | 0.721 |  |  |
| SOC_WLE_ADJ | 0.611 | 0.662 | 0.762 |  |
| ASS_WLE_ADJ | 0.566 | 0.712 |  |  |
| ENE_WLE_ADJ | 0.605 | 0.669 |  |  |
| ST_RELTEACH | 0.639 | 0.470 | 0.707 |  |
| ST_FEEDBACK | 0.389 | 0.771 |  |  |
| ST_STUCLASS | 0.563 | 0.568 |  |  |
| ST_SCHENGAM | 0.812 | 0.694 | 0.848 |  |
| ST_POSEMOT | 0.842 | 0.661 |  |  |
| ST_WELLBEING | 0.524 | 0.958 |  |  |
| Note: The standardized Cronbach's alpha coefficients are 0.773, 0.763, 0.706, and 0.847 | | | | |

Factor structure

From Table 3 (page 16), some loadings show potential cross-loadings (e.g., ENE_WLE_ADJ loads on both Factor 1 and Factor 3 at .525 and .590 respectively). This deserves discussion, particularly given the interpretation of clearly separated constructs.

Under ideal conditions, exploratory factor analysis should yield high loadings for each measured variable on only one latent factor, thereby clearly delineating distinct theoretical constructs. The cross-loading of ENE_WLE_ADJ suggests that the trait of "energy" may not belong exclusively to the domain of skills for interacting with others, as originally hypothesized in the model. Theoretically, a high level of energy (manifested as vitality and active engagement) not only serves as a crucial driver of proactive social interactions (e.g., initiating conversations, participating in group activities) but may also represent an internal resource required for effective emotion regulation (e.g., coping with stress, maintaining positive affective states). Therefore, its significant loadings on two factors may reflect the foundational and cross-cutting role of energy within the system of social-emotional skills, suggesting that it potentially underpins both interpersonal engagement and emotional management processes. This does not invalidate the overall four-factor structure but indicates that the two higher-order constructs, namely "skills for interacting with others" and "emotion regulation skills," may exhibit some degree of conceptual overlap and interrelation in practice, rather than being entirely independent. In the discussion, it can be noted that this finding provides empirical insight into the interconnections among social-emotional skills, implying that certain core attributes (such as energy) may serve as a foundation for the co-development of multiple higher-order competencies.

According to the conventional practices and statistical principles of exploratory factor analysis (EFA), the classification of a variable with significant loadings on multiple factors is not arbitrary but follows clear guidelines. The core principle is to assign the variable to the factor on which it has the highest loading. In this study, the variable "ENE_WLE_ADJ" demonstrated a loading of 0.590 on Factor 3 ("social-emotional skills for engaging with others"), which was higher than its loading of 0.525 on Factor 1 ("social-emotional skills for emotion regulation"). Consequently, when interpreting and naming the factors, it should be classified as part of Factor 3. This indicates that the variable shares the greatest variance with the latent construct of "skills for interacting with others" and holds a stronger theoretical association, suggesting that "energy" is primarily utilized to explain students' activeness and initiative in social interactions.

SEM and model fit

On page 17–18, the manuscript states that “as long as a portion of the indicators meet the standard is sufficient.” This interpretation is methodologically problematic. In addition:

- RMSEA is not reported.
- Chi-square statistics are not reported.
- TLI is not included.
- The reported CFI (0.904) is acceptable but not strong.

The incremental fit indices (CFI, NFI, IFI) met the minimum criteria, indicating that the model demonstrated a significant improvement over the baseline model. The parsimony fit indices (PGFI, PNFI, PCFI) were all greater than 0.50, which is generally considered acceptable. These indices, which penalize model complexity, suggested that the model achieved a reasonable balance between complexity and goodness of fit. The absolute fit index (SRMR) satisfied the commonly used criterion of being less than 0.08, indicating that the residuals of the model were relatively small. Model evaluation followed the principle of holistic judgment; the combination of indices reported in the text constituted a mutually corroborating and logically consistent chain of evidence, collectively demonstrating that the model fit was statistically acceptable. The basic plausibility of the model was confirmed, thereby supporting subsequent path analysis.

Furthermore, with large sample sizes, indices such as the chi-square test of model fit are typically rejected; therefore, this study relied on other fit indices and selectively reported certain indicators. Nevertheless, the existing combination of indices indicated that the model fit was statistically acceptable and could support subsequent structural equation modeling analyses, although it did not achieve perfect fit. This indeed represents a limitation of the current study, and future research should provide more comprehensive evidence of model fit.

Given PLOS ONE’s emphasis on technical rigor, a more cautious and methodologically grounded interpretation of model fit is necessary.

Extremely high coefficients

Some standardized loadings are extremely high, particularly:

- Student development → ST_POSEMOT (β = 0.991)
- Student development → ST_SCHENGAM (β = 0.926)

A loading of 0.991 suggests near-perfect overlap between latent factor and indicator. This raises questions about:

- Construct redundancy,
- Conceptual overlap,
- Potential issues in model specification.

This should at least be acknowledged and discussed.

In the model of this study, some observed variables exhibited excessively high factor loadings on their respective latent variables (e.g., the loadings of positive affect at school and student engagement in school on positive development approached 0.99). This may suggest insufficient statistical discrimination between these indicators and the latent variable, indicating potential construct redundancy or conceptual overlap. Although the overall model fit was adequate, these extremely high loadings warrant a cautious interpretation of the measurement validity for the core construct of "positive development." Future research should further validate the structural validity of this higher-order latent variable by improving measurement instruments (e.g., incorporating more diverse indicators), testing discriminant validity, or employing more complex model specifications (e.g., allowing for correlated residuals) to ensure the robustness of the research findings.

invariance testingCross-national structure

The sample includes students from five sites (Delhi, Dubai, Gunma, Jinan, Kudus; see Table 1, page 15). However:

- No control for site is included,
- No multigroup analysis or invariance testing is performed,
- Clustering is not addressed.

Given the international nature of the dataset, this omission should either be addressed analytically or justified conceptually.

The omission of the "region" factor constitutes a methodological limitation of this study. This decision was made because the study focused more on exploring the pathways of influencing factors at an overall macro-regional level, rather than examining differential effects within factors such as region. In similar multi-site international comparative studies, it is typically necessary to consider the potential impact of differences in culture, educational systems, or socioeconomic backgrounds on the relationships among variables. Neglecting this factor may affect the external validity of the findings, meaning that the results may not be equally generalizable to all five regions. Future research could build upon this study by incorporating region as a control variable, conducting multigroup analysis to test the invariance of the path model across different regions, or employing multilevel modeling to correct for clustering effects at the school or regional level, thereby enabling a more in-depth exploration of cross-cultural or cross-contextual universality and specificity.

Common method bias

All variables are student self-reports. The manuscript briefly mentions this in the limitations, but given the strength of the reported paths (e.g., β = 0.782; β = 0.709), a more explicit discussion of common method variance would be appropriate.

Common method bias is a prevalent potential issue in cross-sectional self-report studies, which may arise from common raters, measurement contexts, item contexts, or scale formats. At a theoretical level, this bias could homogenously inflate the correlations among all variables to some extent, thereby potentially amplifying the magnitude of the path coefficients observed in this study. This necessitates a cautious interpretation when evaluating the absolute strength of the path coefficients (particularly high coefficients), implying that these coefficients should be regarded as "theoretical upper bounds" of the effect sizes.

However, common method bias does not undermine the internal logic of the core theoretical model and hypotheses of this study. The primary contribution of this study lies in testing the existence and relative strength of the theoretical mechanism whereby "school connectedness influences positive development through a multiple mediation chain of social-emotional skills." Even in the presence of common method variance, its effect is more likely to be uniformly distributed across different pathways of the model. Under this assumption, comparisons of the relative importance among variables (e.g., comparing the difference in effect sizes between the two mediating pathways of "interpersonal skills" and "emotion regulation") and the overall goodness of fit of the model may be less affected by the bias than the absolute magnitude of individual path coefficients. Our main conclusion, that school connectedness promotes student development by cultivating specific social-emotional competencies, relies precisely on this structured pathway relationship, rather than merely on high correlations.

To enhance the persuasiveness of the findings, this study implemented preventive statistical controls during the data analysis phase, including ensuring adequate discriminant validity of the scales in confirmatory factor analysis and presenting detailed model fit indices when reporting the results.

**4. Interpretation and causal language**

The study uses cross-sectional data, yet terms such as “promotes” and “fosters” are frequently used throughout the abstract, results, discussion, and conclusion.

Revised as requested.

Given the design, the findings demonstrate statistical associations rather than causal pathways. The language should be moderated accordingly.

Revised as requested.

Also, in section 2.3 the manuscript acknowledges a bidirectional relation between interpersonal skills and emotion regulation, but in the SEM model the direction is unidirectional (engaging with others → emotion regulation). This theoretical tension should be explained more clearly.

The discussion of bidirectional relationships in the theoretical section stems from a broad theoretical understanding of how these two skills interact with and mutually reinforce each other in real-life contexts, revealing their potential and dynamic interconnections. However, the empirical model in this study was designed to test a specific, sequential pathway hypothesis, namely, to investigate how "school connectedness" influences "emotion regulation skills" through "interpersonal skills," ultimately promoting positive development among students. This represents a causal chain model proposed for testing based on a particular theoretical perspective (e.g., social cognitive theory). It does not deny the existence of bidirectional relationships but rather focuses on examining whether the effect in this specific direction is significant and quantifying its magnitude. When constructing models, researchers often need to establish specific directional hypotheses based on theory or research questions, and unidirectional path models are more common and clearer in such mediation analyses. Therefore, the discussion of bidirectional relationships in the theoretical section provides a more comprehensive background, while the unidirectional paths in the SEM model represent an empirical test of one specific influence mechanism. The two are complementary rather than contradictory. Future research could more directly examine the bidirectional causal relationships between these two skills using longitudinal data or cross-lagged panel models.

**5. Ethics statement**

In the submission system, the Ethics Statement is marked as “N/A.” However, the study analyzes human participant data (students), even if secondary.

Since SSES data are public-use and anonymized, the manuscript should clarify:

- That the data are fully de-identified,
- That they are publicly accessible,
- Why additional ethical approval was not required.

Leaving the statement as “N/A” is not appropriate for research involving human participants.

That the data are fully de-identified.

That they are publicly accessible. The use of publicly available SSES data has been ethically approved.

**6. Data availability**

Although “Yes – all data are fully available without restriction” is selected, the Data Availability Statement states that the data can be obtained by emailing the authors (page 5).

This does not comply with PLOS ONE’s Data Policy. Because the SSES dataset is publicly available through OECD, the authors should provide:

- The official repository link or DOI,
- A clear statement that data are publicly available without restriction.

This is essential for compliance.

The data link is 10.6084/m9.figshare.31832035

**7. Clarity and language**

The manuscript is generally understandable but would benefit from careful language revision. There are grammatical inconsistencies (e.g., punctuation issues, repeated structures, phrasing such as “There are may fit indices”), and some sections are repetitive.

Because PLOS ONE does not provide copyediting after acceptance, attention to language clarity is important before resubmission.

Revised as requested.

**Overall evaluation**

The manuscript addresses a relevant research question using a large and valuable dataset. The proposed sequential mediation model is conceptually coherent and has potential to contribute to the literature on school connectedness and social-emotional development.

However, to meet PLOS ONE’s criteria for technically rigorous research, substantial revisions are needed, particularly in:

- Measurement validation and reliability reporting,
- Statistical transparency and model fit interpretation,
- Treatment of the international sample structure,
- Adjustment of causal language,
- Correction of Ethics and Data Availability statements.

With these revisions, the study could meet the journal’s standard for inclusion in the scientific record.

Revised as requested.
