# Supplementary material for: Exploring the impact path of school connectedness on student development through cultivating students’ social-emotional skills
Source: PLoS One. 2026 Jul 22;21(7):e0353972. doi: 10.1371/journal.pone.0353972 (PMC13390845; doi:10.1371/journal.pone.0353972)
Supplement: Attachment — Submitted filename: Review Comments to the Author.docx [file pone.0353972.s001.docx]

**Review Comments to the Author**

Thank you for the opportunity to review this manuscript. The paper addresses an important and timely question: how school connectedness relates to students’ positive development through specific social-emotional skills, using a large sample from the OECD SSES 2023 Asian dataset (N = 23,560). The topic is clearly relevant to contemporary educational research and fits within the scope of PLOS ONE, particularly given its emphasis on methodological soundness rather than perceived novelty.

Below, I provide detailed comments structured around the journal’s publication criteria and focused primarily on technical rigor, clarity, and compliance with editorial standards.

**1. Originality of the research**

The manuscript presents empirical analyses of a large international dataset and proposes a sequential mediation model (“school connectedness → engaging with others → emotion regulation → positive development”). This appears to constitute original research rather than a review or secondary analysis without added value.

That said, since the SSES 2023 dataset is publicly available and has already generated multiple reports and analyses, the manuscript would benefit from more clearly articulating what is analytically new. In particular:

- Has this specific sequential mediation model already been tested elsewhere with SSES 2023 data?
- In what way does this model advance beyond existing associations reported in OECD publications or recent empirical studies?

The originality lies mainly in the proposed chain mediation structure, and making this contribution more explicit would strengthen the positioning of the study.

**2. Prior publication of results**

There are no obvious signs that the analyses have been published elsewhere. However, since the dataset is public and frequently analyzed, I recommend including a short clarification stating that:

- The structural model presented has not been previously published.
- The figures and tables are original.

Such transparency helps avoid ambiguity in secondary data analyses.

**3. Technical and statistical standards**

This is the main area where revision is needed.

Measurement model and reliability

On page 15, a single Cronbach’s alpha (α = 0.795) is reported, but it is not clear to which construct this refers. Given that four latent constructs are modeled, reliability should be reported for each construct separately. Moreover:

- Composite Reliability (CR) and Average Variance Extracted (AVE) are not reported.
- Discriminant validity is not tested.
- An Exploratory Factor Analysis (EFA) is conducted, even though the constructs come from an established OECD framework.

If the constructs are theory-driven and defined a priori, a Confirmatory Factor Analysis (CFA) would be more appropriate than EFA. At minimum, the choice of EFA should be justified.

Additionally, the description “the data reliability is high” seems overly strong based solely on a single alpha value.

Factor structure

From Table 3 (page 16), some loadings show potential cross-loadings (e.g., ENE_WLE_ADJ loads on both Factor 1 and Factor 3 at .525 and .590 respectively). This deserves discussion, particularly given the interpretation of clearly separated constructs.

SEM and model fit

On page 17–18, the manuscript states that “as long as a portion of the indicators meet the standard is sufficient.” This interpretation is methodologically problematic. In addition:

- RMSEA is not reported.
- Chi-square statistics are not reported.
- TLI is not included.
- The reported CFI (0.904) is acceptable but not strong.

Given PLOS ONE’s emphasis on technical rigor, a more cautious and methodologically grounded interpretation of model fit is necessary.

Extremely high coefficients

Some standardized loadings are extremely high, particularly:

- Student development → ST_POSEMOT (β = 0.991)
- Student development → ST_SCHENGAM (β = 0.926)

A loading of 0.991 suggests near-perfect overlap between latent factor and indicator. This raises questions about:

- Construct redundancy,
- Conceptual overlap,
- Potential issues in model specification.

This should at least be acknowledged and discussed.

Cross-national structure

The sample includes students from five sites (Delhi, Dubai, Gunma, Jinan, Kudus; see Table 1, page 15). However:

- No control for site is included,
- No multigroup analysis or invariance testing is performed,
- Clustering is not addressed.

Given the international nature of the dataset, this omission should either be addressed analytically or justified conceptually.

Common method bias

All variables are student self-reports. The manuscript briefly mentions this in the limitations, but given the strength of the reported paths (e.g., β = 0.782; β = 0.709), a more explicit discussion of common method variance would be appropriate.

**4. Interpretation and causal language**

The study uses cross-sectional data, yet terms such as “promotes” and “fosters” are frequently used throughout the abstract, results, discussion, and conclusion.

Given the design, the findings demonstrate statistical associations rather than causal pathways. The language should be moderated accordingly.

Also, in section 2.3 the manuscript acknowledges a bidirectional relation between interpersonal skills and emotion regulation, but in the SEM model the direction is unidirectional (engaging with others → emotion regulation). This theoretical tension should be explained more clearly.

**5. Ethics statement**

In the submission system, the Ethics Statement is marked as “N/A.” However, the study analyzes human participant data (students), even if secondary.

Since SSES data are public-use and anonymized, the manuscript should clarify:

- That the data are fully de-identified,
- That they are publicly accessible,
- Why additional ethical approval was not required.

Leaving the statement as “N/A” is not appropriate for research involving human participants.

**6. Data availability**

Although “Yes – all data are fully available without restriction” is selected, the Data Availability Statement states that the data can be obtained by emailing the authors (page 5).

This does not comply with PLOS ONE’s Data Policy. Because the SSES dataset is publicly available through OECD, the authors should provide:

- The official repository link or DOI,
- A clear statement that data are publicly available without restriction.

This is essential for compliance.

**7. Clarity and language**

The manuscript is generally understandable but would benefit from careful language revision. There are grammatical inconsistencies (e.g., punctuation issues, repeated structures, phrasing such as “There are may fit indices”), and some sections are repetitive.

Because PLOS ONE does not provide copyediting after acceptance, attention to language clarity is important before resubmission.

**Overall evaluation**

The manuscript addresses a relevant research question using a large and valuable dataset. The proposed sequential mediation model is conceptually coherent and has potential to contribute to the literature on school connectedness and social-emotional development.

However, to meet PLOS ONE’s criteria for technically rigorous research, substantial revisions are needed, particularly in:

- Measurement validation and reliability reporting,
- Statistical transparency and model fit interpretation,
- Treatment of the international sample structure,
- Adjustment of causal language,
- Correction of Ethics and Data Availability statements.

With these revisions, the study could meet the journal’s standard for inclusion in the scientific record.
