# Supplementary material for: Exploring the impact path of school connectedness on student development through cultivating students’ social-emotional skills
Source: PLoS One. 2026 Jul 22;21(7):e0353972. doi: 10.1371/journal.pone.0353972 (PMC13390845; doi:10.1371/journal.pone.0353972)
Supplement: Attachment — Submitted filename: Response_to_Reviewers_auresp_2.docx [file pone.0353972.s005.docx]

Dear Dr. Wang,

Thank you for submitting your manuscript to PLOS ONE. After careful consideration, we feel that it has merit but does not fully meet PLOS ONE’s publication criteria as it currently stands. Therefore, we invite you to submit a revised version of the manuscript that addresses the points raised during the review process.

While the reviewers acknowledge the merit of the revisions made, they still suggest further improvements regarding both the introduction and the discussion of the results, as well as the statistical section.

Please submit your revised manuscript by Jun 29 2026 11:59PM. If you will need more time than this to complete your revisions, please reply to this message or contact the journal office at plosone@plos.org. When you're ready to submit your revision, log on to https://www.editorialmanager.com/pone/ and select the 'Submissions Needing Revision' folder to locate your manuscript file.

Please include the following items when submitting your revised manuscript:

A letter that responds to each point raised by the academic editor and reviewer(s). You should upload this letter as a separate file labeled 'Response to Reviewers'.

A marked-up copy of your manuscript that highlights changes made to the original version. You should upload this as a separate file labeled 'Revised Manuscript with Track Changes'.

An unmarked version of your revised paper without tracked changes. You should upload this as a separate file labeled 'Manuscript'.

If you would like to make changes to your financial disclosure, please include your updated statement in your cover letter. Guidelines for resubmitting your figure files are available below the reviewer comments at the end of this letter.

If applicable, we recommend that you deposit your laboratory protocols in protocols.io to enhance the reproducibility of your results. Protocols.io assigns your protocol its own identifier (DOI) so that it can be cited independently in the future. For instructions see: https://journals.plos.org/plosone/s/submission-guidelines#loc-laboratory-protocols. Additionally, PLOS ONE offers an option for publishing peer-reviewed Lab Protocol articles, which describe protocols hosted on protocols.io. Read more information on sharing protocols at https://plos.org/protocols?utm_medium=editorial-email&utm_source=authorletters&utm_campaign=protocols.

As the corresponding author, your ORCID iD is verified in the submission system and will appear in the published article. PLOS supports the use of ORCID, and we encourage all coauthors to register for an ORCID iD and use it as well. Please encourage your coauthors to verify their ORCID iD within the submission system before final acceptance, as unverified ORCID iDs will not appear in the published article. Only the individual author can complete the verification step; PLOS staff cannot verify ORCID iDs on behalf of authors.

1. If the reviewer comments include a recommendation to cite specific previously published works, please review and evaluate these publications to determine whether they are relevant and should be cited. There is no requirement to cite these works unless the editor has indicated otherwise.

2. Please review your reference list to ensure that it is complete and correct. If you have cited papers that have been retracted, please include the rationale for doing so in the manuscript text, or remove these references and replace them with relevant current references. Any changes to the reference list should be mentioned in the rebuttal letter that accompanies your revised manuscript. If you need to cite a retracted article, indicate the article’s retracted status in the References list and also include a citation and full reference for the retraction notice.

1. If the authors have adequately addressed your comments raised in a previous round of review and you feel that this manuscript is now acceptable for publication, you may indicate that here to bypass the “Comments to the Author” section, enter your conflict of interest statement in the “Confidential to Editor” section, and submit your "Accept" recommendation.

Reviewer #1: All comments have been addressed

Reviewer #2: (No Response)

2. Is the manuscript technically sound, and do the data support the conclusions?

The manuscript must describe a technically sound piece of scientific research with data that supports the conclusions. Experiments must have been conducted rigorously, with appropriate controls, replication, and sample sizes. The conclusions must be drawn appropriately based on the data presented.

Reviewer #1: Partly

Reviewer #2: Partly

3. Has the statistical analysis been performed appropriately and rigorously?

Reviewer #1: No

Reviewer #2: No

4. Have the authors made all data underlying the findings in their manuscript fully available?

The PLOS Data policy requires authors to make all data underlying the findings described in their manuscript fully available without restriction, with rare exception (please refer to the Data Availability Statement in the manuscript PDF file). The data should be provided as part of the manuscript or its supporting information, or deposited to a public repository. For example, in addition to summary statistics, the data points behind means, medians and variance measures should be available. If there are restrictions on publicly sharing data—e.g. participant privacy or use of data from a third party—those must be specified.

Reviewer #1: Yes

Reviewer #2: Yes

5. Is the manuscript presented in an intelligible fashion and written in standard English?

PLOS ONE does not copyedit accepted manuscripts, so the language in submitted articles must be clear, correct, and unambiguous. Any typographical or grammatical errors should be corrected at revision, so please note any specific errors here.

Reviewer #1: Yes

Reviewer #2: Yes

6. Review Comments to the Author

Please use the space provided to explain your answers to the questions above. You may also include additional comments for the author, including concerns about dual publication, research ethics, or publication ethics. (Please upload your review as an attachment if it exceeds 20,000 characters)

Reviewer #1: Review of revised manuscript (PONE-D-25-65886R1)

The revised manuscript addresses an important and timely research question using a large international dataset (OECD SSES 2023). The authors have made several improvements in response to the first round of review, particularly regarding data availability, clarification of ethical aspects, and the inclusion of additional measurement indices (e.g., CR, AVE, discriminant validity).

However, despite these revisions, several substantial methodological and reporting issues remain insufficiently addressed, particularly with regard to statistical rigor and the interpretation of SEM results. These issues limit the extent to which the manuscript can currently be considered technically sound.

Below are the main points that still require attention.

Major comments

1.

Model fit reporting and interpretation (pp. 34–36) Although additional fit indices have been included, some key indicators are still missing, in particular:

•

RMSEA (with confidence interval)

•

Chi-square statistic and degrees of freedom

The manuscript continues to justify model adequacy based on a selective set of indices, stating that a “holistic judgment” is sufficient. This interpretation remains problematic. Standard SEM practice requires complete and transparent reporting of model fit indices, and conclusions should be based on the overall pattern of indices rather than partial compliance.

In addition, the reported values (e.g., CFI ≈ 0.90; TLI ≈ 0.90; SRMR = 0.081) indicate an acceptable but not strong fit, which should be acknowledged more explicitly rather than presented as fully satisfactory.

The required indicator data was provided as requested, along with explanatory notes and discussions. For example, results that exactly met the standard threshold were described as acceptable rather than completely ideal. For results that deviated from the standard values or slightly exceeded the threshold, these were also presented, discussed, and explained.

The specific main text is as follows:

The model fitting is acceptable. The model fitting indicators are as follows. In this study, CFI=0.904>0.9, NFI=0.904>0.9, IFI=0.904>0.9, PGFI=0.570>0.5, PNFI=0.685>0.5, PCFI=0.685>0.5. Goodness-of-fit indices should typically exceed 0.9 [38]. Indices exceeding 0.50 serves as the minimum acceptable threshold for models, representing the most fundamental baseline. When indices meet or exceed 0.50, the model demonstrates basic explanatory power. These findings indicate that this study satisfies the standards for acceptable fit [39, 40, 41]. The incremental fit indices (CFI, NFI, IFI) met the minimum criteria, indicating a significant improvement of the proposed model over the baseline model. The parsimony fit indices (PGFI, PNFI, PCFI) were all above 0.50, which is generally considered acceptable. These indices penalize model complexity and suggest that the model achieves a reasonable balance between complexity and goodness-of-fit.

In this study, χ²/df = 358. Under conditions of a very large sample size, the chi-square test is almost always significant, and the chi-square-to-degrees-of-freedom ratio becomes excessively inflated; consequently, the model fit based on the chi-square test is typically rejected. Fit indices such as the RMSEA and TLI are substantially influenced by model complexity, sample size, and the distributional characteristics of the observed variables. Given the extremely large sample size and the relatively large number of observed indicators in the model, the RMSEA in this study was 0.123. Although slightly above conventional thresholds, this value is considered acceptable, and its 90% confidence interval [0.110, 0.129] indicates a relatively stable estimate. The TLI was 0.874, approaching the common benchmark of 0.90, and is deemed acceptable given the complexity of the model. The SRMR was 0.109, slightly above the recommended threshold of 0.10. The SRMR reflects the average discrepancy between the model-implied covariance matrix and the sample covariance matrix. In models involving a large number of observed variables (12 in this study) and complex path relationships, a slight elevation in the SRMR is a common phenomenon. An SRMR value below 0.10 is generally considered acceptable, while values between 0.10 and 0.12 can be regarded as marginally acceptable.

Furthermore, in this study, all hypothesized paths were statistically significant (p<0.001), with standardized path coefficients ranging from 0.187 to 0.782, consistent with theoretical expectations. The R²values for the endogenous latent variables were as follows: Engaging with others (0.611), Emotion regulation (0.503), and School performance (0.379), indicating that the model exhibited moderate to strong explanatory power for the core variables. Model evaluation adhered to the principle of holistic judgment. Although some fit indices in this study did not reach the optimal levels suggested by conventional benchmarks, the overall model demonstrated acceptable fit given the practical constraints of an extremely large sample size and a complex measurement structure.

Model evaluation adhered to the principle of holistic judgment. Although some fit indices in this study did not reach the optimal levels suggested by conventional benchmarks, the overall model demonstrated acceptable fit given the practical constraints of an extremely large sample size and a complex measurement structure.

2.

Treatment of the hierarchical data structure (p. 30; Table 1) The dataset includes students nested within different contexts (five cities); however:

•

No control variables for site are included

•

No multigroup or multilevel analyses have been conducted

•

No correction for clustering has been applied

This issue was already raised in the first round of review and is now mentioned only as a limitation. However, given the size and structure of the dataset, it remains a methodologically relevant omission.

At a minimum, the authors should:

•

include site (and, if available, other covariates such as gender or socioeconomic status) as control variables or

•

provide a stronger methodological justification for not modeling the nested structure or

•

at least indicate, in the future directions section, alternative analytical approaches and the use of statistical techniques that account for the hierarchical nature of the data

In response to the revision suggestions, this study added relevant content that includes city and gender as control variables in the structural equation model analysis.

The specific main text is as follows:

Based on this, this study included students’ city of residence and gender as control variables in the structural equation model. After scaling the observed variables to reduce differences in variable scales, the structural equation model with site and gender as control variables converged normally. The results showed that all core structural paths remained significant. To improve numerical stability, the observed indicators were rescaled before SEM estimation. WLE-based indicators were divided by 100, and student scale indicators were divided by 10. Standardized coefficients were used for interpretation.

The data for this study were collected from five cities: Delhi (India), Dubai (UAE), Gunma (Japan), Jinan (China), and Kudus (Indonesia), with Delhi (India, DEL) serving as the reference category. When the control variable is a five-level categorical variable, including four dummy variables allows for complete group comparisons, with the omitted category automatically serving as the reference group. Its effect is absorbed into the model intercept and therefore does not appear in the path coefficient estimates. Accordingly, the model included four categorical control variables for the cities (DUB, GUN, JIN, KUD) and one demographic control variable for gender (gender_Std). The MLR estimator was used to examine the effects on paths among latent variables. The variance inflation factor (VIF) for all control variables was below 3, indicating no serious multicollinearity concerns.

Regarding the effects of control variables on Engaging with others, the standardized effect for DUB was 0.044, indicating that respondents in this group exhibited relatively higher levels of Engaging with others. The effect for GUN was -0.221 (p<0.001), representing the strongest negative influence among the control variables, suggesting that students from Gunma had significantly lower levels of Engaging with others. The effect for JIN was not statistically significant (β=0.005, p=0.589). The effect for KUD was 0.019 (p=0.033), indicating a weak positive influence. The standardized effect for gender was 0.092 (p<0.001), indicating that male students exhibited higher levels of Engaging with others compared to female students.

In terms of effects on Emotion regulation, the effect for DUB was not significant (β=-0.008, p=0.371). GUN showed a significant positive effect (β=0.148), indicating that students from Gunma had higher levels of Emotion regulation. The effects for JIN (β=0.072) and KUD (β=0.115) both positively predicted Emotion regulation. Gender also demonstrated a positive effect (β=0.093, p<0.001), with male students showing relatively higher levels of Emotion regulation.

Regarding effects on Student development, the effect for DUB was 0.038 (p<0.001), indicating a weak positive influence. The effect for GUN was 0.027 (p=0.006), representing a relatively weak positive influence. The effect for JIN was not statistically significant (β=0.003, p=0.797). The positive effect for KUD was the most pronounced (β=0.174, p<0.001), making it the strongest control variable influencing Student development, suggesting that students from Kudus demonstrated better developmental outcomes. The effect for gender was 0.013 (p=0.026), indicating only a weak predictive role.

In summary, the results of the control variables indicate regional differences. Using Delhi as the reference group, students from Gunma showed significantly lower levels of Engaging with others, whereas students from Kudus demonstrated the most prominent performance in Student development. Students from Jinan exhibited no significant differences from the reference group across any dimension. Students from Dubai showed slightly higher levels only in the Engaging with others dimension compared to the reference group. At the demographic level, male students exhibited a weak but statistically significant advantage across all dimensions. Overall, the differential impact of geographical and cultural backgrounds on student development was more pronounced than that of gender.

3.

Extremely high factor loadings (pp. 35–36) Some loadings remain extremely high (e.g., student development → positive emotions β ≈ 0.99). The manuscript acknowledges this as a limitation, which is appropriate.

However, the implications are not fully developed. Values of this magnitude suggest:

•

possible construct redundancy

•

limited discriminant validity at the higher-order level

A more cautious interpretation of the “student development” construct is needed, with a more explicit discussion of the potential overlap among indicators.

In accordance with the revision suggestions, corresponding explanatory notes have been added.

The specific main text is as follows:

The high factor loading of Student positive emotion (close to 1.0) may indicate potential construct redundancy or substantial conceptual overlap with the latent variable of student positive development. Although school engagement and student wellbeing were both included in the study, student positive emotion almost entirely represents the overall latent construct of student positive development. This phenomenon may be attributed to the strong theoretical interrelatedness of this indicator with the other two dimensions (particularly school engagement), or it may suggest that, given the current measurement tools and sample, the three dimensions are empirically difficult to distinguish, thereby compromising the discriminant validity of the higher-order construct. Therefore, when interpreting the path effects of student positive development in this study, one should avoid equating it simply with a broad, multidimensional developmental state. Instead, it is more appropriate to understand it as a locally composite indicator dominated by student positive emotion, while also incorporating elements of academic engagement behaviors and basic wellbeing.

4.

Use of EFA with theoretically defined constructs (pp. 31–33) The manuscript now includes both EFA and CFA. However, the rationale for conducting EFA remains weak, considering that:

•

the constructs are defined a priori (OECD framework)

•

a confirmatory approach would be expected

The current justification (EFA followed by CFA) does not fully resolve the initial concern. The authors should either:

•

provide a clearer methodological rationale for this choice or

•

emphasize the confirmatory framework more consistently

As requested, the corresponding part of EFA has been removed, and only CFA was conducted.

5.

Conceptual framing and consistency (Introduction and Discussion) The cultural dimension (Asian context), introduced in the Introduction, remains insufficiently developed in the Discussion.

As requested, the research findings were discussed in the context of the Asian scenario.

The specific main text is as follows:

From an Asian perspective, education systems across the region generally emphasize collectivism and relational harmony. Against this backdrop, peer relationships and teacher-student relationships not only serve as core components of school belonging but also function as the “relational soil” for the development of students’ social-emotional skills. “Student-peer relationships” and “perceived teacher-student relationships” contribute most significantly to school belonging, which aligns closely with Asian cultural traditions that value interpersonal bonds and respect for authority (e.g., respect for teachers and moral education) [49] (Stremfel et al., 2024). Asian students’ sense of school belonging relies more on high-quality interpersonal interactions than on mere institutional affiliation [50].

Asian students generally face high levels of academic pressure [51]. Among emotion regulation skills, “stress tolerance” and “optimism” are particularly prominent in predicting positive development. This finding may reflect the “adaptive function” of emotion regulation skills within the Asian cultural context. Students require not only general emotional control abilities but also specific skills to cope with pressure and maintain positive expectations in highly competitive and evaluative educational environments [52].

Because Asian cultures encourage self-regulation and interpersonal restraint within the collective, students first obtain group identification and emotional support through positive interpersonal interactions (e.g., cooperation, empathy), which then become internalized as individual-level emotion management abilities [53]. This contrasts with the Western individualistic approach, where emotion regulation is often cultivated as an independent individual skill. Therefore, the model proposed in this study is not only statistically valid but also culturally interpretable [54].

The facilitating effect of school belonging on social-emotional skills is not a universal linear process; rather, it presents a culturally specific chain-mediated pathway within the Asian educational ecology characterized by collectivism, high academic pressure, and interpersonal orientation.

Urban differences can be cross-interpreted through dimensions such as cultural value variations, educational system orientations, and social support structures. The significantly lower performance of students in Gunma, Japan, on “Engaging with others” may be related to the “high-context restraint” characteristic of East Asian collectivist culture. Social norms in Japanese society emphasize individual integration into the collective rather than proactively expanding interpersonal connections, leading students to prefer maintaining distance and avoiding excessive self-disclosure in interactions. Such cultural traits tend to suppress measured scores on “actively interacting with others and participating in group activities” [55]. In contrast, cities such as Dubai in the United Arab Emirates, serving as multicultural immigration hubs, exhibit greater tolerance for open and extroverted interactions, which naturally enhances students’ willingness to engage interpersonally [56].

The outstanding performance of students in Kudus, Indonesia, on “Student development” can be attributed to the strong buffering effect of its community support network. Indonesia is a typical society characterized by high collectivism and strong religious ties. Non-major cities such as Kudus retain close-knit clan and community mutual aid traditions, where students’ academic pressure and psychological distress are quickly buffered by families and communities. Additionally, as local education is in an ascending phase, policies tend to strongly support the development of disadvantaged groups, enabling students to exhibit “catch-up advantages” in comprehensive development indicators such as academic achievement and mental health [57]. In contrast, Jinan, China, as a typical East Asian educational hub, experiences high competitive pressure among students that offsets some of the resource dividends, resulting in no significant difference from the reference group.

Regarding gender differences, the weak but consistent advantage of male students aligns with the bias characteristics of developmental stages and measurement tools in adolescence. The measurement of “Emotion regulation” in this study is largely based on “problem-solving-oriented emotional coping”. Although adolescent males exhibit higher impulsivity than females, they tend to report higher self-efficacy in the use of instrumental emotion management strategies [58].

49.Johnson RG, Allen KA, Gallo Cordoba B. Where does culture belong at school? Exploring the role of individualism and power distance in school belonging across cultures. Curr Psychol. 2023. doi: 10.1007/s12144-023-05280-y.

50.Stremfel U, Sterman Ivančič K, Peras I. Addressing the sense of school belonging among all students? A systematic literature review. Eur J Investig Health Psychol Educ. 2024;14:2901–2917. doi: 10.3390/ejihpe14110190.

51.Alshareef N, Fletcher I, Giga S. The role of emotions in academic performance of undergraduate medical students: A narrative review. BMC Med Educ. 2024;24:907. doi: 10.1186/s12909-024-05894-1.

52.de Castro CCF, Malazarte NJM. Exploring school-based emotion regulation programs for Asian adolescents: Recommendations based on a scoping review. Soc Sci Lens. 2025;6(1):20–32. doi: 10.62718/vmca.ssl-wjhdsr.6.1.SC-0925-005.

53.Ni W, Chen L. Effects of physical activity on college students’ interpersonal competence: Chain-mediated effects of self-control and prosocial interpersonal emotional management. Front Psychol. 2025;16:1649527. doi: 10.3389/fpsyg.2025.1649527.

54.Jiang W, Yin H. Functioning, but not necessarily adaptive: Unravelling the adaptive and maladaptive teachers’ emotion regulation. Asia-Pac Educ Res. 2026;35(1):5–14. doi: 10.1007/s40299-025-00986-y.

55.Koyanagi Y, Aung MN, Yuasa M, Sekine M, Takao O. The relation between social capital and academic motivation of students: A study of health professional education in Japan. Eur J Investig Health Psychol Educ. 2021;11(1):129–141. doi: 10.3390/ejihpe11010011.

56.Lam SF, Jimerson S, Shin H, Cefai C, Veiga FH, Hatzichristou C, Polychroni F, Kikas E, Wong BPH, Stanculescu E, Basnett J, Duck R, Farrell P, Liu Y, Negovan V, Nelson B, Yang H, Zollneritsch J. Cultural universality and specificity of student engagement in school: The results of an international study from 12 countries. Br J Educ Psychol. 2015;85(1):137–153. doi: 10.1111/bjep.12060.

57.Irasanti SN, Repati T, Januarita R, Yunairti Y, Chen HWJ, Marzo RR. Domain and perception on community resilience: Comparison between two countries. Front Public Health. 2023;11:1157837. doi: 10.3389/fpubh.2023.1157837.

58.Zhao X, Zhang RZ, Zheng K. Gender differences in emotion regulation strategies in adolescents. Chin J Clin Psychol. 2014;22(5):849–854.

6.

Causal language There has been some improvement in reducing causal language (e.g., increased use of “predicts” and “is associated with”). However, some formulations still suggest directional or causal interpretations that are not fully supported by the cross-sectional design.

Further refinement toward strictly associational language is recommended.

The corresponding expressions were revised as requested.

Minor comments

•

The manuscript is clearer than in the previous version, but language and style still require revision, particularly to reduce repetition and improve fluency.

•

Some sections remain overly verbose or redundant, especially in the Results.

Redundant and repetitive expressions have been removed.

Overall evaluation The manuscript has improved in several respects, particularly in terms of transparency (data availability, ethical clarification) and partial strengthening of the measurement model. However, for the reasons outlined above, further revisions are still required

Reviewer #2: Introduction

The theoretical framework is presented primarily in the Method section, rather than being clearly and systematically articulated in the Introduction. In its current form, the Introduction remains comparatively long and would benefit from further condensation.

There is a reference to the framework of self-determination theory, even though the stated theoretical point of reference is social cognitive theory.

The introduction was revised as requested. All theoretical reference points have been uniformly changed to social cognitive theory.

The following passage also seems inappropriate for the Introduction, as it reads more like an interpretation of results or a contribution statement that would be better placed in the Discussion or removed:

“This finding not only confirms that school connectedness predicts student development through the dual mediation of interpersonal and emotion regulation skills, but also elucidates the hierarchical and sequential nature of skill development—specifically, that interpersonal competence serves as an effective precursor to the development of emotion regulation. By clarifying the internal transmission process of “environment → skill sequence → developmental outcome,” this study provides a theoretical foundation and precise targets for school-based interventions designed around the sequential cultivation of social-emotional skills”

It was deleted as requested.

Literature review

Later in the literature review, the manuscript states:

“It is worth noting that the empirical model in this study is designed to test a specific, sequential pathway of influence, namely, to explore how school coNNNectedness influences interpersonal skills, which in turn affects emotional regulation skills, ultimately contributing to positive student development. This represents a causal chain model proposed for examination from a particular theoretical perspective (social cognitive theory). While it does not deny the existence of bidirectional relationships, it focuses on testing whether this specific directional pathway is significant and quantifying its effect size.”

In my opinion, this paragraph is misplaced. It reads more as a justification of the analytic model and would fit better in the introduction after the research question.

This part has been placed after the research questions as requested.

Materials and methods

The amount of missing data is still unclear and should be explicitly reported.

The official dataset (SSES R2 ST PUF INTERNATIONAL) contained 65,613 data entries, of which 23,560 were relevant to the measures and regional subjects of focus in this study. Irrelevant item data were removed using the listwise deletion method. Among the subjects and items involved in this study, there were no missing data.

The statement “the data were assumed to approximately meet the analytical requirements” does not provide sufficient information regarding normality assumptions, and no robustness checks are reported.

As requested, the relevant content of normality test analysis and robustness test analysis has been added.

The commonly used maximum likelihood estimation in SEM typically assumes that the observed data are derived from a multivariate normal distribution. In this study. This study conducted a normality test to examine whether the quantitative data followed a normal distribution. Given the large sample size, the Kolmogorov-Smirnov (K-S) test was used. The results showed significant values for all variables, indicating that the data did not conform to a normal distribution. However, normality assumptions are often stringent and difficult to meet in practice. In this study, the absolute values of kurtosis were less than 10, and the absolute values of skewness were less than 3 for all items, suggesting that although the data were not perfectly normal, they were generally acceptable as approximately normally distributed (See Table 1).

**Table 1. Results of the Normality Test (Kolmogorov–Smirnov Test)**

|  | **Mean** | **SD** | **Skewness** | **Kurtosis** | **K-S D statistic** | ***p*** |
| --- | --- | --- | --- | --- | --- | --- |
| **STR_WLE_ADJ** | 529.806 | 95.540 | 0.735 | 4.943 | 0.072 | 0.000** |
| **OPT_WLE_ADJ** | 567.422 | 96.560 | 0.581 | 1.435 | 0.060 | 0.000** |
| **EMO_WLE_ADJ** | 538.946 | 107.487 | 0.687 | 3.605 | 0.083 | 0.000** |
| **SOC_WLE_ADJ** | 557.435 | 96.428 | 0.697 | 3.333 | 0.065 | 0.000** |
| **ASS_WLE_ADJ** | 539.760 | 98.355 | 1.102 | 11.240 | 0.097 | 0.000** |
| **ENE_WLE_ADJ** | 537.860 | 102.446 | 0.934 | 5.148 | 0.069 | 0.000** |
| **ST_RELTEACH** | 51.774 | 10.551 | 0.626 | 0.308 | 0.186 | 0.000** |
| **ST_FEEDBACK** | 53.739 | 10.239 | -0.003 | -0.163 | 0.137 | 0.000** |
| **ST_STUCLASS** | 52.845 | 11.166 | 0.767 | 1.014 | 0.193 | 0.000** |
| **ST_SCHENGAM** | 51.993 | 10.986 | 0.069 | 0.443 | 0.102 | 0.000** |
| **ST_POSEMOT** | 51.452 | 11.195 | 0.182 | 0.727 | 0.104 | 0.000** |
| **ST_WELLBEING** | 51.885 | 10.744 | 0.137 | 0.810 | 0.106 | 0.000** |

To examine the robustness of the mediating mechanism, this study conducted 5,000 bootstrap resampling tests after controlling for city and gender. The model converged normally, and all 5,000 bootstrap iterations were successfully completed. The results showed that the indirect effect of school connectedness on student development through the ability of engaging with others was significant, Estimate = 0.391, SE = 0.022, 95% BC bootstrap CI [0.350, 0.438]. The serial mediating effect of school connectedness on student development through both the ability of engaging with others and emotion regulation was also significant, Estimate = 0.109, SE = 0.017, 95% BC bootstrap CI [0.074, 0.141]. The total indirect effect was also significant, Estimate = 0.500, SE = 0.011, 95% BC bootstrap CI [0.479, 0.520]. Given that none of the above confidence intervals contained zero, the mediating mechanism remained valid and robust after controlling for site and gender.

Results & discussion

Although a CFA has now been added, the manuscript still does not justify the initial use of EFA.

The cross-loading of the wellbeing factor was acknowledged, but its implications were not adequately examined. In particular, the manuscript does not discuss whether this pattern may indicate conceptual overlap, weak construct specification, or possible model misspecification, nor does it consider alternative modeling solutions.

The corresponding content of EFA has been removed, and only CFA was used.

Comment from first review round: Given the hierarchical structure of the data (students nested within schools, and schools nested within countries), multilevel analyses would be methodologically advisable. At a minimum, statistical controls for site, gender, age/grade, and socioeconomic status (if available) should be included to account for contextual variability.

At present, this issue has been addressed only as a limitation and not at the analytical level.

In response to the revision suggestions, this study added relevant content that includes city and gender as control variables in the structural equation model analysis.

The specific main text is as follows:

Based on this, this study included students’ city of residence and gender as control variables in the structural equation model. After scaling the observed variables to reduce differences in variable scales, the structural equation model with site and gender as control variables converged normally. The results showed that all core structural paths remained significant. To improve numerical stability, the observed indicators were rescaled before SEM estimation. WLE-based indicators were divided by 100, and student scale indicators were divided by 10. Standardized coefficients were used for interpretation.

The data for this study were collected from five cities: Delhi (India), Dubai (UAE), Gunma (Japan), Jinan (China), and Kudus (Indonesia), with Delhi (India, DEL) serving as the reference category. When the control variable is a five-level categorical variable, including four dummy variables allows for complete group comparisons, with the omitted category automatically serving as the reference group. Its effect is absorbed into the model intercept and therefore does not appear in the path coefficient estimates. Accordingly, the model included four categorical control variables for the cities (DUB, GUN, JIN, KUD) and one demographic control variable for gender (gender_Std). The MLR estimator was used to examine the effects on paths among latent variables. The variance inflation factor (VIF) for all control variables was below 3, indicating no serious multicollinearity concerns.

Regarding the effects of control variables on Engaging with others, the standardized effect for DUB was 0.044, indicating that respondents in this group exhibited relatively higher levels of Engaging with others. The effect for GUN was -0.221 (p<0.001), representing the strongest negative influence among the control variables, suggesting that students from Gunma had significantly lower levels of Engaging with others. The effect for JIN was not statistically significant (β=0.005, p=0.589). The effect for KUD was 0.019 (p=0.033), indicating a weak positive influence. The standardized effect for gender was 0.092 (p<0.001), indicating that male students exhibited higher levels of Engaging with others compared to female students.

In terms of effects on Emotion regulation, the effect for DUB was not significant (β=-0.008, p=0.371). GUN showed a significant positive effect (β=0.148), indicating that students from Gunma had higher levels of Emotion regulation. The effects for JIN (β=0.072) and KUD (β=0.115) both positively predicted Emotion regulation. Gender also demonstrated a positive effect (β=0.093, p<0.001), with male students showing relatively higher levels of Emotion regulation.

Regarding effects on Student development, the effect for DUB was 0.038 (p<0.001), indicating a weak positive influence. The effect for GUN was 0.027 (p=0.006), representing a relatively weak positive influence. The effect for JIN was not statistically significant (β=0.003, p=0.797). The positive effect for KUD was the most pronounced (β=0.174, p<0.001), making it the strongest control variable influencing Student development, suggesting that students from Kudus demonstrated better developmental outcomes. The effect for gender was 0.013 (p=0.026), indicating only a weak predictive role.

In summary, the results of the control variables indicate regional differences. Using Delhi as the reference group, students from Gunma showed significantly lower levels of Engaging with others, whereas students from Kudus demonstrated the most prominent performance in Student development. Students from Jinan exhibited no significant differences from the reference group across any dimension. Students from Dubai showed slightly higher levels only in the Engaging with others dimension compared to the reference group. At the demographic level, male students exhibited a weak but statistically significant advantage across all dimensions. Overall, the differential impact of geographical and cultural backgrounds on student development was more pronounced than that of gender.

Because the analysis is based on cross-sectional, observational survey data, the manuscript should avoid causal wording. The reported SEM can identify associations compatible with mediation, but it cannot establish causal mediation or temporal ordering.

The wording has been revised as requested.

The authors state that RMSEA, chi-square statistics, and TLI were omitted because they slightly deviated from recommended thresholds. However, key SEM fit indices should be reported transparently regardless of whether they meet conventional criteria. Any deviations should be acknowledged and discussed as part of the overall model-fit evaluation.

The required indicator data was provided as requested, along with explanatory notes and discussions. For example, results that exactly met the standard threshold were described as acceptable rather than completely ideal. For results that deviated from the standard values or slightly exceeded the threshold, these were also presented, discussed, and explained.

The specific main text is as follows:

The model fitting is acceptable. The model fitting indicators are as follows. In this study, CFI=0.904>0.9, NFI=0.904>0.9, IFI=0.904>0.9, PGFI=0.570>0.5, PNFI=0.685>0.5, PCFI=0.685>0.5. Goodness-of-fit indices should typically exceed 0.9 [38]. Indices exceeding 0.50 serves as the minimum acceptable threshold for models, representing the most fundamental baseline. When indices meet or exceed 0.50, the model demonstrates basic explanatory power. These findings indicate that this study satisfies the standards for acceptable fit [39, 40, 41]. The incremental fit indices (CFI, NFI, IFI) met the minimum criteria, indicating a significant improvement of the proposed model over the baseline model. The parsimony fit indices (PGFI, PNFI, PCFI) were all above 0.50, which is generally considered acceptable. These indices penalize model complexity and suggest that the model achieves a reasonable balance between complexity and goodness-of-fit.

In this study, χ²/df = 358. Under conditions of a very large sample size, the chi-square test is almost always significant, and the chi-square-to-degrees-of-freedom ratio becomes excessively inflated; consequently, the model fit based on the chi-square test is typically rejected. Fit indices such as the RMSEA and TLI are substantially influenced by model complexity, sample size, and the distributional characteristics of the observed variables. Given the extremely large sample size and the relatively large number of observed indicators in the model, the RMSEA in this study was 0.123. Although slightly above conventional thresholds, this value is considered acceptable, and its 90% confidence interval [0.110, 0.129] indicates a relatively stable estimate. The TLI was 0.874, approaching the common benchmark of 0.90, and is deemed acceptable given the complexity of the model. The SRMR was 0.109, slightly above the recommended threshold of 0.10. The SRMR reflects the average discrepancy between the model-implied covariance matrix and the sample covariance matrix. In models involving a large number of observed variables (12 in this study) and complex path relationships, a slight elevation in the SRMR is a common phenomenon. An SRMR value below 0.10 is generally considered acceptable, while values between 0.10 and 0.12 can be regarded as marginally acceptable.

Furthermore, in this study, all hypothesized paths were statistically significant (p<0.001), with standardized path coefficients ranging from 0.187 to 0.782, consistent with theoretical expectations. The R²values for the endogenous latent variables were as follows: Engaging with others (0.611), Emotion regulation (0.503), and School performance (0.379), indicating that the model exhibited moderate to strong explanatory power for the core variables. Model evaluation adhered to the principle of holistic judgment. Although some fit indices in this study did not reach the optimal levels suggested by conventional benchmarks, the overall model demonstrated acceptable fit given the practical constraints of an extremely large sample size and a complex measurement structure.

Model evaluation adhered to the principle of holistic judgment. Although some fit indices in this study did not reach the optimal levels suggested by conventional benchmarks, the overall model demonstrated acceptable fit given the practical constraints of an extremely large sample size and a complex measurement structure.

General comments

More generally, the manuscript would benefit from careful streamlining and stylistic polishing. A thorough round of proofreading and corrections would enhance readability and may also help remove residual errors (e.g. Subsequently, student interpersonal skills are hypothesized not only to directly enhance their positive development but also to further promote their emotion regulationl skills.)

The wording has been revised as requested.

7. PLOS authors have the option to publish the peer review history of their article (what does this mean?). If published, this will include your full peer review and any attached files.

If you choose “no”, your identity will remain anonymous but your review may still be made public.

Do you want your identity to be public for this peer review? For information about this choice, including consent withdrawal, please see our Privacy Policy.

Reviewer #1: No

Reviewer #2: No

[NOTE: If reviewer comments were submitted as an attachment file, they will be attached to this email and accessible via the submission site. Please log into your account, locate the manuscript record, and check for the action link "View Attachments". If this link does not appear, there are no attachment files.]

To ensure your figures meet our technical requirements, please review our figure guidelines: https://journals.plos.org/plosone/s/figures

You may also use PLOS’s free figure tool, NAAS, to help you prepare publication quality figures: https://journals.plos.org/plosone/s/figures#loc-tools-for-figure-preparation.

NAAS will assess whether your figures meet our technical requirements by comparing each figure against our figure specifications.
