# Supplementary material for: Exploring the impact path of school connectedness on student development through cultivating students’ social-emotional skills
Source: PLoS One. 2026 Jul 22;21(7):e0353972. doi: 10.1371/journal.pone.0353972 (PMC13390845; doi:10.1371/journal.pone.0353972)
Supplement: Attachment — Submitted filename: Response_to_Reviewers_auresp_3.docx [file pone.0353972.s006.docx]

PONE-D-25-65886R2

Exploring the impact path of school connectedness on student development through cultivating students' social emotional skills

PLOS One

Dear Dr. Wang,

Thank you for submitting your manuscript to PLOS ONE. After careful consideration, we feel that it has merit but does not fully meet PLOS ONE’s publication criteria as it currently stands. Therefore, we invite you to submit a revised version of the manuscript that addresses the points raised during the review process.

The reviewers and I acknowledge that the authors have carefully addressed the concerns raised during the previous rounds of review and that the manuscript has been substantially strengthened as a result. However, a few minor revisions are still required before the manuscript can be considered for publication. In particular:

- The article would benefit from another round of proofreading (There are some mistakes: e.g., The commonly used maximum likelihood estimation in SEM typically assumes that the observed data are derived from a multivariate normal distribution. IN THIS STUDY. This study conducted a normality test to examine whether the quantitative data followed a normal distribution.).

All revisions in this Revised Manuscript with Track Changes are highlighted in blue.

The entire manuscript has undergone another round of proofreading for grammar, expression, and data accuracy.

The confusing sentence in the preceding text has been deleted. Keep only this sentence: “This study conducted a normality test to examine whether the quantitative data followed a normal distribution”.

Also in the section related to robustness checks, it is written: "In this study, the absolute values of kurtosis were less than 10". However, according to the table ASS_WLE_ADJ = 11.240

Only the kurtosis value for ASS_WLE_ADJ was 11.240, which, nonetheless, remained well below the liberal threshold (|kurtosis| < 20). The kurtosis values for all other variables fell within reasonable ranges. Accordingly, the data can be considered to exhibit acceptable univariate normality, supporting the use of maximum likelihood estimation.

- some methodological concerns remain, particularly with respect to the interpretation of SEM results and the overall adequacy of model fit. The reported values (RMSEA = .123, TLI = .874, SRMR = .109) remain indicative of a model that demonstrates only marginal to moderate fit. While the authors appropriately acknowledge some of these limitations, the interpretation remains somewhat optimistic. The limitations associated with model fit should be explicitly recognized when discussing the strength of the conclusions.

Given that several fit indices of the structural equation model reached only acceptable levels, the model demonstrated marginal to moderate fit, falling short of an optimal level and merely meeting the threshold for acceptability. Therefore, while the analysis of the significance and directional consistency of the path coefficients can be considered highly reliable, the interpretation of effect sizes for each path should be approached with caution and appropriate flexibility, and should be positioned as exploratory findings. Comparisons regarding the magnitude of effects across different paths warrant further refinement through model modification or validation using independent samples.

Please submit your revised manuscript by Jul 31 2026 11:59PM. If you will need more time than this to complete your revisions, please reply to this message or contact the journal office at plosone@plos.org. When you're ready to submit your revision, log on to https://www.editorialmanager.com/pone/ and select the 'Submissions Needing Revision' folder to locate your manuscript file.

Please include the following items when submitting your revised manuscript:

A letter that responds to each point raised by the academic editor and reviewer(s). You should upload this letter as a separate file labeled 'Response to Reviewers'.

A marked-up copy of your manuscript that highlights changes made to the original version. You should upload this as a separate file labeled 'Revised Manuscript with Track Changes'.

An unmarked version of your revised paper without tracked changes. You should upload this as a separate file labeled 'Manuscript'.

If you would like to make changes to your financial disclosure, please include your updated statement in your cover letter. Guidelines for resubmitting your figure files are available below the reviewer comments at the end of this letter.

If applicable, we recommend that you deposit your laboratory protocols in protocols.io to enhance the reproducibility of your results. Protocols.io assigns your protocol its own identifier (DOI) so that it can be cited independently in the future. For instructions see: https://journals.plos.org/plosone/s/submission-guidelines#loc-laboratory-protocols. Additionally, PLOS ONE offers an option for publishing peer-reviewed Lab Protocol articles, which describe protocols hosted on protocols.io. Read more information on sharing protocols at <https://plos.org/protocols?utm_medium=editorial-email&utm_source=authorletters&utm_campaign=protocols.>

As the corresponding author, your ORCID iD is verified in the submission system and will appear in the published article. PLOS supports the use of ORCID, and we encourage all coauthors to register for an ORCID iD and use it as well. Please encourage your coauthors to verify their ORCID iD within the submission system before final acceptance, as unverified ORCID iDs will not appear in the published article. Only the individual author can complete the verification step; PLOS staff cannot verify ORCID iDs on behalf of authors.

We look forward to receiving your revised manuscript.

Kind regards,

Carlotta Rivella

Guest Editor

PLOS One

Journal Requirements:

If the reviewer comments include a recommendation to cite specific previously published works, please review and evaluate these publications to determine whether they are relevant and should be cited. There is no requirement to cite these works unless the editor has indicated otherwise.

Please review your reference list to ensure that it is complete and correct. If you have cited papers that have been retracted, please include the rationale for doing so in the manuscript text, or remove these references and replace them with relevant current references. Any changes to the reference list should be mentioned in the rebuttal letter that accompanies your revised manuscript. If you need to cite a retracted article, indicate the article’s retracted status in the References list and also include a citation and full reference for the retraction notice.

[Note: HTML markup is below. Please do not edit.]

Reviewers' comments:

Reviewer's Responses to Questions

Comments to the Author

1. If the authors have adequately addressed your comments raised in a previous round of review and you feel that this manuscript is now acceptable for publication, you may indicate that here to bypass the “Comments to the Author” section, enter your conflict of interest statement in the “Confidential to Editor” section, and submit your "Accept" recommendation.

Reviewer #1: All comments have been addressed

Reviewer #2: All comments have been addressed

2. Is the manuscript technically sound, and do the data support the conclusions?

The manuscript must describe a technically sound piece of scientific research with data that supports the conclusions. Experiments must have been conducted rigorously, with appropriate controls, replication, and sample sizes. The conclusions must be drawn appropriately based on the data presented.

Reviewer #1: Yes

Reviewer #2: Yes

3. Has the statistical analysis been performed appropriately and rigorously?

Reviewer #1: Yes

Reviewer #2: (No Response)

4. Have the authors made all data underlying the findings in their manuscript fully available?

The PLOS Data policy requires authors to make all data underlying the findings described in their manuscript fully available without restriction, with rare exception (please refer to the Data Availability Statement in the manuscript PDF file). The data should be provided as part of the manuscript or its supporting information, or deposited to a public repository. For example, in addition to summary statistics, the data points behind means, medians and variance measures should be available. If there are restrictions on publicly sharing data—e.g. participant privacy or use of data from a third party—those must be specified.

Reviewer #1: Yes

Reviewer #2: Yes

5. Is the manuscript presented in an intelligible fashion and written in standard English?

PLOS ONE does not copyedit accepted manuscripts, so the language in submitted articles must be clear, correct, and unambiguous. Any typographical or grammatical errors should be corrected at revision, so please note any specific errors here.

Reviewer #1: Yes

Reviewer #2: Yes

6. Review Comments to the Author

Please use the space provided to explain your answers to the questions above. You may also include additional comments for the author, including concerns about dual publication, research ethics, or publication ethics. (Please upload your review as an attachment if it exceeds 20,000 characters)

Reviewer #1: The authors have made substantial efforts to address the concerns raised in the previous review round. In particular, they improved the transparency of model reporting, included additional measurement information, removed the exploratory factor analysis, incorporated control variables, expanded the discussion of cultural aspects, and reduced the use of causal language.

The manuscript has been strengthened considerably compared to the previous version. While some concerns remain regarding the overall quality of model fit and the limitations associated with the SEM results, these issues are now reported and discussed more explicitly. Although the reported fit indices do not indicate an optimal model fit, the authors have acknowledged these limitations and provided additional justification for their analytical choices.

Overall, I believe that the major concerns raised in the previous review have been adequately addressed and that the manuscript is now suitable for publication.

Reviewer #2: The article would benefit from another round of proofreading (There are some mistakes: e.g., The commonly used maximum likelihood estimation in SEM typically assumes that the observed data are derived from a multivariate normal distribution. IN THIS STUDY. This study conducted a normality test to examine whether the quantitative data followed a normal distribution.)

The confusing sentence in the preceding text has been deleted. Keep only this sentence: “This study conducted a normality test to examine whether the quantitative data followed a normal distribution”.

Also in the section related to robustness checks, it is written: "In this study, the absolute values of kurtosis were less than 10". However, according to the table ASS_WLE_ADJ = 11.240

Only the kurtosis value for ASS_WLE_ADJ was 11.240, which, nonetheless, remained well below the liberal threshold (|kurtosis| < 20). The kurtosis values for all other variables fell within reasonable ranges. Accordingly, the data can be considered to exhibit acceptable univariate normality, supporting the use of maximum likelihood estimation.

7. PLOS authors have the option to publish the peer review history of their article (what does this mean?). If published, this will include your full peer review and any attached files.

If you choose “no”, your identity will remain anonymous but your review may still be made public.

Do you want your identity to be public for this peer review? For information about this choice, including consent withdrawal, please see our Privacy Policy.

Reviewer #1: No

Reviewer #2: No

[NOTE: If reviewer comments were submitted as an attachment file, they will be attached to this email and accessible via the submission site. Please log into your account, locate the manuscript record, and check for the action link "View Attachments". If this link does not appear, there are no attachment files.]

To ensure your figures meet our technical requirements, please review our figure guidelines: https://journals.plos.org/plosone/s/figures

You may also use PLOS’s free figure tool, NAAS, to help you prepare publication quality figures: https://journals.plos.org/plosone/s/figures#loc-tools-for-figure-preparation.

NAAS will assess whether your figures meet our technical requirements by comparing each figure against our figure specifications.
