# Supplementary material for: Exploring the impact path of school connectedness on student development through cultivating students’ social-emotional skills
Source: PLoS One. 2026 Jul 22;21(7):e0353972. doi: 10.1371/journal.pone.0353972 (PMC13390845; doi:10.1371/journal.pone.0353972)
Supplement: Peer review history

# Peer review: Decision Letter 0

**DOI:** 10.1371/journal.pone.0353972.r001

**Authors:** Carlotta Rivella

25 Feb 2026

PONE-D-25-65886

Exploring the impact path of school connectedness on student development through cultivating students' social-emotional skills

PLOS One

Dear Dr. Wang,

Thank you for submitting your manuscript to PLOS ONE. After careful consideration, we feel that it has merit but does not fully meet PLOS ONE’s publication criteria as it currently stands. Therefore, we invite you to submit a revised version of the manuscript that addresses the points raised during the review process.

Please submit your revised manuscript by Apr 11 2026 11:59PM. If you will need more time than this to complete your revisions, please reply to this message or contact the journal office at plosone@plos.org. When you're ready to submit your revision, log on to https://www.editorialmanager.com/pone/ and select the 'Submissions Needing Revision' folder to locate your manuscript file.

Please include the following items when submitting your revised manuscript:

- A letter that responds to each point raised by the academic editor and reviewer(s). You should upload this letter as a separate file labeled 'Response to Reviewers'.
- A marked-up copy of your manuscript that highlights changes made to the original version. You should upload this as a separate file labeled 'Revised Manuscript with Track Changes'.
- An unmarked version of your revised paper without tracked changes. You should upload this as a separate file labeled 'Manuscript'.

If you would like to make changes to your financial disclosure, please include your updated statement in your cover letter. Guidelines for resubmitting your figure files are available below the reviewer comments at the end of this letter.

If applicable, we recommend that you deposit your laboratory protocols in protocols.io to enhance the reproducibility of your results. Protocols.io assigns your protocol its own identifier (DOI) so that it can be cited independently in the future. For instructions see: https://journals.plos.org/plosone/s/submission-guidelines#loc-laboratory-protocols. Additionally, PLOS ONE offers an option for publishing peer-reviewed Lab Protocol articles, which describe protocols hosted on protocols.io. Read more information on sharing protocols at https://plos.org/protocols?utm_medium=editorial-email&utm_source=authorletters&utm_campaign=protocols.

We look forward to receiving your revised manuscript.

Kind regards,

Carlotta Rivella

Guest Editor

PLOS One

Journal Requirements:

When submitting your revision, we need you to address these additional requirements.

1.Please ensure that your manuscript meets PLOS ONE's style requirements, including those for file naming. The PLOS ONE style templates can be found at

https://journals.plos.org/plosone/s/file?id=wjVg/PLOSOne_formatting_sample_main_body.pdf and

https://journals.plos.org/plosone/s/file?id=ba62/PLOSOne_formatting_sample_title_authors_affiliations.pdf

2. In the online submission form you indicate that your data is not available for proprietary reasons and have provided a contact point for accessing this data. Please note that your current contact point is a co-author on this manuscript. According to our Data Policy, the contact point must not be an author on the manuscript and must be an institutional contact, ideally not an individual. Please revise your data statement to a non-author institutional point of contact, such as a data access or ethics committee, and send this to us via return email. Please also include contact information for the third party organization, and please include the full citation of where the data can be found.

3. Please amend either the title on the online submission form (via Edit Submission) or the title in the manuscript so that they are identical.

4. Please ensure that you refer to Figure 1 in your text as, if accepted, production will need this reference to link the reader to the figure.

5. We note you have included a table to which you do not refer in the text of your manuscript. Please ensure that you refer to Table 1-5 in your text; if accepted, production will need this reference to link the reader to the Table.

6. If the reviewer comments include a recommendation to cite specific previously published works, please review and evaluate these publications to determine whether they are relevant and should be cited. There is no requirement to cite these works unless the editor has indicated otherwise.

Additional Editor Comments:

Dear Authors,

after carefully reading your manuscript and considering the reviewers’ comments, we recognize the potential value of your work. However, there are substantial methodological and statistical limitations that currently prevent the manuscript from being suitable for publication.

Accordingly, we are requesting a major revision. We strongly encourage you to carefully address the reviewers’ suggestions, as implementing these revisions will significantly improve the quality and rigor of your manuscript.

When submitting your revised manuscript, please include a detailed response letter that outlines how each reviewer and editor comment has been addressed. If you decide not to follow a particular suggestion, provide a clear justification for your choice.

Carlotta Rivella

Reviewers' comments:

Reviewer's Responses to Questions

**Comments to the Author**

1. Is the manuscript technically sound, and do the data support the conclusions?

The manuscript must describe a technically sound piece of scientific research with data that supports the conclusions. Experiments must have been conducted rigorously, with appropriate controls, replication, and sample sizes. The conclusions must be drawn appropriately based on the data presented.

Reviewer #1: Partly

Reviewer #2: Partly

**********

2. Has the statistical analysis been performed appropriately and rigorously?

Reviewer #1: No

Reviewer #2: No

**********

3. Have the authors made all data underlying the findings in their manuscript fully available?

The PLOS Data policy requires authors to make all data underlying the findings described in their manuscript fully available without restriction, with rare exception (please refer to the Data Availability Statement in the manuscript PDF file). The data should be provided as part of the manuscript or its supporting information, or deposited to a public repository. For example, in addition to summary statistics, the data points behind means, medians and variance measures should be available. If there are restrictions on publicly sharing data—e.g. participant privacy or use of data from a third party—those must be specified.

Reviewer #1: No

Reviewer #2: Yes

**********

4. Is the manuscript presented in an intelligible fashion and written in standard English?

PLOS ONE does not copyedit accepted manuscripts, so the language in submitted articles must be clear, correct, and unambiguous. Any typographical or grammatical errors should be corrected at revision, so please note any specific errors here.

Reviewer #1: No

Reviewer #2: Yes

**********

5. Review Comments to the Author

Please use the space provided to explain your answers to the questions above. You may also include additional comments for the author, including concerns about dual publication, research ethics, or publication ethics. (Please upload your review as an attachment if it exceeds 20,000 characters)

Reviewer #1: The manuscript addresses a relevant research question using a large international dataset (OECD SSES 2023, N = 23,560) and applies structural equation modeling to test a sequential mediation model. The topic is timely and potentially suitable for PLOS ONE. However, several issues must be addressed before the study can be considered technically sound and compliant with journal standards.

First, the statistical analysis, while generally appropriate in terms of method selection, is not reported with sufficient rigor. The measurement model requires stronger validation. Reliability is reported only once (Cronbach’s alpha = 0.795), without specifying to which construct it refers. Reliability indices should be provided separately for each latent construct. Additionally, Composite Reliability (CR), Average Variance Extracted (AVE), and evidence of discriminant validity are not reported. Given that the constructs are theoretically predefined based on OECD SSES, the rationale for conducting an exploratory factor analysis (EFA) instead of a confirmatory factor analysis (CFA) should be clarified and justified.

Model fit reporting is incomplete. Important indices such as RMSEA, TLI, and chi-square statistics are not presented. The interpretation of model fit is overly permissive (e.g., stating that it is sufficient for only some indices to meet thresholds). This should be revised in line with standard SEM reporting practices.

Some standardized coefficients are extremely high (e.g., loadings approaching or exceeding 0.99), suggesting potential construct overlap or redundancy. These values require closer examination and discussion.

Second, the Data Availability Statement does not comply with PLOS ONE policy. Although the submission indicates that all data are available without restriction, the manuscript states that data can be obtained by emailing the authors. Since the OECD SSES dataset is publicly available, the authors should provide the official repository link or DOI and clearly state that the data are publicly accessible without restriction.

Third, the Ethics Statement is currently listed as “N/A.” Even though the study uses secondary, anonymized data, the manuscript should clarify that the data are fully de-identified and publicly available, and explain why additional ethical approval was not required.

Fourth, the manuscript contains multiple grammatical and stylistic issues (e.g., phrasing such as “There are may fit indices”) and repetitive structures. Because PLOS ONE does not provide copyediting after acceptance, careful language revision is necessary before resubmission.

Finally, the study is based on cross-sectional data, yet causal language (e.g., “promotes,” “fosters”) is used throughout. Interpretations should be reframed in terms of statistical associations rather than causal effects.

In summary, while the dataset and overall modeling approach have potential, substantial revisions are required to meet the technical, reporting, and editorial standards of the journal.

Reviewer #2: INTRODUCTION

The theoretical framework is presented within the Method section rather than being clearly articulated in the Introduction.

The Introduction is comparatively long, somewhat redundant, and insufficiently supported by relevant citations. A more concise structure and a stronger integration of the existing literature would improve clarity and strengthen the study.

Although the focus on the Asian educational context is explicitly mentioned in the Introduction, this cultural perspective is not revisited in the remainder of the manuscript. The cultural dimension should be explicitly addressed again in the Discussion, Conclusion, Implications and Limitations section.

METHOD AND RESULTS

Preliminary analyses are not reported and should be explicitly included to strengthen the methodological transparency of the study. In particular, the manuscript would benefit from reporting:

• Reliability indices for each dimension (in addition to any global alpha coefficient);

• A transparent description of the handling of missing data;

• A clear justification of the estimator used (e.g., ML, MLR, or WLSMV), including its appropriateness given the measurement scale of the items;

• Information regarding normality assumptions and any robustness checks conducted;

• Effect sizes alongside statistical significance, especially considering the large sample size;

• A sensitivity analysis addressing the potential impact of self-report bias.

Additional methodological clarifications are needed:

It remains unclear whether the analyses were conducted at the item level or at the scale-score level. The coding structure suggests the use of specific indicators, whereas the narrative refers to broader latent dimensions. This discrepancy should be clarified to ensure transparency and replicability.

The use of Exploratory Factor Analysis (EFA) appears inconsistent with the presence of a clearly stated theoretical model. When a priori theoretical assumptions are available, Confirmatory Factor Analysis (CFA) would generally be more appropriate. The rationale for choosing EFA should therefore be justified.

The wellbeing factor shows cross-loadings on two scales with very similar loadings, raising concerns regarding discriminant validity and potential model misspecification. This issue requires further examination and discussion.

Given the hierarchical structure of the data (students nested within schools, and schools nested within countries), multilevel analyses would be methodologically advisable. At a minimum, statistical controls for site, gender, age/grade, and socioeconomic status (if available) should be included to account for contextual variability.

Model fit indices appear relatively weak considering the large sample size. Although chi-square statistics are known to be highly sensitive in large samples, marginal or inadequate fit cannot be justified solely by noting that some indices meet recommended thresholds. .

Finally, the statistical significance of the factors reported in the table is not clearly indicated and should be explicitly provided.

DISCUSSION

The theoretical interpretation lacks consistency. Although the model is introduced as grounded in Social Cognitive Theory (SCT), the Discussion shifts toward an interpretation framed primarily in terms of Self-Determination Theory (SDT). This theoretical drift creates conceptual ambiguity and should be resolved.

Furthermore, the manuscript appears to imply causal relationships that are not warranted by the study design. Given the correlational nature of the data, the interpretations should be carefully reformulated to reflect associative rather than causal conclusions.

FORM AND WRITING STYLE

The manuscript is excessively schematized in several sections, which affects readability and academic tone. Some passages are redundant or insufficiently formal. For example, the statement:

“The model fitting indicators are as follows. There are may fit indices. It is difficult for all indicators to meet the standard, so as long as a portion of the indicators meet the standard is sufficient.”

is problematic both linguistically and conceptually. Beyond language concerns, the underlying methodological claim is questionable and requires a more rigorous justification.

FORM AND WRITING STYLE (minor)

The expression “social emotional skills” sometimes appears repetitive throughout the manuscript. Revising certain sentences to reduce redundancy would enhance overall readability.

DATA AVAILABILITY (minor)

Although the manuscript states that the data are accessible, providing a direct link to the dataset would substantially improve transparency and reproducibility.

**********

6. PLOS authors have the option to publish the peer review history of their article (what does this mean?). If published, this will include your full peer review and any attached files.

If you choose “no”, your identity will remain anonymous but your review may still be made public.

**Do you want your identity to be public for this peer review?**  For information about this choice, including consent withdrawal, please see our Privacy Policy.

Reviewer #1: No

Reviewer #2: **Yes:**  Simone Pinetti

**********

[NOTE: If reviewer comments were submitted as an attachment file, they will be attached to this email and accessible via the submission site. Please log into your account, locate the manuscript record, and check for the action link "View Attachments". If this link does not appear, there are no attachment files.]

To ensure your figures meet our technical requirements, please review our figure guidelines: https://journals.plos.org/plosone/s/figures

You may also use PLOS’s free figure tool, NAAS, to help you prepare publication quality figures: https://journals.plos.org/plosone/s/figures#loc-tools-for-figure-preparation.

NAAS will assess whether your figures meet our technical requirements by comparing each figure against our figure specifications.

Attachment Submitted filename: Review Comments to the Author.docx

# Author comment: Author response to Decision Letter 1

**DOI:** 10.1371/journal.pone.0353972.r002

23 Mar 2026

Responses to Review Comments to the Author

Thank you for the opportunity to review this manuscript. The paper addresses an important and timely question: how school connectedness relates to students’ positive development through specific social-emotional skills, using a large sample from the OECD SSES 2023 Asian dataset (N = 23,560). The topic is clearly relevant to contemporary educational research and fits within the scope of PLOS ONE, particularly given its emphasis on methodological soundness rather than perceived novelty.

Below, I provide detailed comments structured around the journal’s publication criteria and focused primarily on technical rigor, clarity, and compliance with editorial standards.

1. Originality of the research

The manuscript presents empirical analyses of a large international dataset and proposes a sequential mediation model (“school connectedness → engaging with others → emotion regulation → positive development”). This appears to constitute original research rather than a review or secondary analysis without added value.

That said, since the SSES 2023 dataset is publicly available and has already generated multiple reports and analyses, the manuscript would benefit from more clearly articulating what is analytically new. In particular:

•Has this specific sequential mediation model already been tested elsewhere with SSES 2023 data?

•In what way does this model advance beyond existing associations reported in OECD publications or recent empirical studies?

This specific sequential mediation model has not been tested elsewhere and represents an innovative model first proposed by this study.

The official OECD publications only provide analyses of the results from specific questionnaire items and the analysis of weighted relevant items. In contrast, this study conducted a more in-depth analysis utilizing the MS variables (Student Weight and Scores) provided in the official OECD publications. Specifically, these variables were treated as observed indicators to further summarize and synthesize new influencing factors. Therefore, this study represents a deeper exploration and dissection of existing resources. Compared with other empirical studies, this research employed structural equation modeling, which offers advantages over simple linear regression. The path coefficients in SEM are directional, allowing for a clear decomposition of the pathways through which influencing factors operate. Concurrently, the overall pathways in structural equation modeling are more complex than those in simple mediation models, which facilitates the inclusion of all factors in a comprehensive analysis and enables a全景展示 of the research findings.

The originality lies mainly in the proposed chain mediation structure, and making this contribution more explicit would strengthen the positioning of the study.

The primary original contribution of this study lies in empirically testing and revealing, through structural equation modeling, a clear sequential mediation pathway: "school connectedness → interpersonal skills → emotion regulation skills → positive development." Existing literature has largely focused on the direct association between school connectedness and development, or on single-dimensional mediating effects, lacking an integrated analysis of the synergistic effects of multidimensional social-emotional skills and their developmental sequence. This study not only verifies that school connectedness influences student development through the dual mediation of interpersonal skills and emotion regulation skills, but more importantly, it elucidates the sequential relationship between these two skills, namely, that enhanced interpersonal skills serve as an effective antecedent for the development of emotion regulation capacity. This finding clarifies the hierarchical and sequential nature of social-emotional skill development, providing a new theoretical basis and precise targets for designing school interventions based on the detailed process of "environment → skill sequence → developmental outcomes."

2. Prior publication of results

There are no obvious signs that the analyses have been published elsewhere. However, since the dataset is public and frequently analyzed, I recommend including a short clarification stating that:

•The structural model presented has not been previously published.

•The figures and tables are original.

Such transparency helps avoid ambiguity in secondary data analyses.

The structural model presented has not been previously published. This represents an innovative exploration based on publicly available data conducted in this study. The figures and tables are original.

3. Technical and statistical standards

This is the main area where revision is needed.

Measurement model and reliability

On page 15, a single Cronbach’s alpha (α = 0.795) is reported, but it is not clear to which construct this refers. Given that four latent constructs are modeled, reliability should be reported for each construct separately. Moreover:

•Composite Reliability (CR) and Average Variance Extracted (AVE) are not reported.

•Discriminant validity is not tested.

•An Exploratory Factor Analysis (EFA) is conducted, even though the constructs come from an established OECD framework.

In the assessment of convergent validity, although the Average Variance Extracted (AVE) values for some constructs fell slightly below the recommended threshold of 0.5 (0.485 and 0.492, respectively), the Composite Reliability (CR) for all constructs exceeded the suggested criterion of 0.7. According to Fornell and Larcker (1981), convergent validity can still be considered adequate when CR values meet the standard, even if AVE is marginally below 0.5. Furthermore, all item factor loadings were statistically significant at the p < 0.001 level, providing additional support for the convergent validity of the scales (See Table 7).

Table 7. Results of Model AVE and CR Indicators

Factor AVE CR

School connectedness 0.485 0.729

Engaging with others 0.492 0.744

Emotion regulation 0.509 0.757

Student development 0.711 0.875

Fornell, C., & Larcker, D. F. (1981). Evaluating Structural Equation Models with Unobservable Variables and Measurement Error. Journal of Marketing Research, 18(1), 39–50. https://doi.org/10.1177/002224378101800104

Confirmatory factor analysis (CFA) can be used to differentiate validity studies. The diagonal line in the table represents the AVE square root value, while the remaining values represent the correlation coefficients. The AVE square root value can represent the aggregation of factors, and the correlation coefficient represents the correlation relationship. If the aggregation of factors is strong (significantly stronger than the absolute value of the correlation coefficient with other factors), it can indicate discriminant validity; If the square root of a factor's AVE is greater than the absolute value of its correlation coefficient with other factors. And if all factors present such a conclusion, it indicates good discriminant validity. Therefore, all factors have good discriminant validity (See Table 8).

Table 8. Discriminant validity: Pearson correlation and AVE square root value

School connectedness Engaging with others Emotion regulation Student development

School connectedness 0.696

Engaging with others 0.430 0.702

Emotion regulation 0.473 0.588 0.714

Student development 0.505 0.521 0.549 0.843

If the constructs are theory-driven and defined a priori, a Confirmatory Factor Analysis (CFA) would be more appropriate than EFA. At minimum, the choice of EFA should be justified.

Based on exploratory factor analysis, this study supplements with confirmatory factor analysis (CFA). This analysis focuses on four factors and twelve analytical items for CFA. The effective sample size for this analysis is 23,560, which is more than ten times the number of analytical items, indicating a moderate sample size. The factor loading coefficient values reflect the correlation between factors (latent variables) and analytical items (observed variables/measurement items). The standardized loading coefficient values represent the correlation between factors and analytical items (measurement items). All items exhibit significance, and the standardized loading coefficient values are greater than 0.4, indicating a strong correlation (See Table 5).

Table 5. Factor loading coefficient table

Factor Observed variable Coef. Std. Error z (CR) p Std. Estimate SMC

School connectedness ST_RELTEACH 1.000 - - - 0.783 0.614

ST_FEEDBACK 0.579 0.009 64.130 0.000 0.467 0.218

ST_STUCLASS 1.067 0.011 96.084 0.000 0.789 0.623

Engaging with others SOC_WLE_ADJ 1.000 - - - 0.691 0.477

ASS_WLE_ADJ 0.974 0.011 85.889 0.000 0.679 0.461

ENE_WLE_ADJ 1.175 0.013 90.627 0.000 0.734 0.539

Emotion regulation STR_WLE_ADJ 1.000 - - - 0.716 0.512

OPT_WLE_ADJ 1.054 0.011 96.224 0.000 0.737 0.544

EMO_WLE_ADJ 1.023 0.011 91.011 0.000 0.687 0.472

Student development ST_SCHENGAM 1.000 - - - 0.927 0.860

ST_POSEMOT 1.088 0.004 250.981 0.000 0.990 0.981

ST_WELLBEING 0.570 0.006 94.684 0.000 0.541 0.293

Note: The dash “-” indicates that the item is a reference item.

The model fit indices of the confirmatory factor analysis are as follows. When testing the measurement model, the results of the confirmatory factor analysis indicate that the model fits well, and all indices meet the recommended standards (See Table 6).

Table 6. Measurement model fitting index

Common indicators GFI CFI NFI NNFI TLI IFI PGFI PNFI PCFI SRMR

Judgment criteria >0.9 >0.9 >0.9 >0.9 >0.9 >0.9 >0.5 >0.5 >0.5 <0.1

Value 0.917 0.930 0.929 0.903 0.903 0.930 0.564 0.676 0.676 0.081

Additionally, the description “the data reliability is high” seems overly strong based solely on a single alpha value.

The reliability indicators for each dimension also meet the standards (See Table 2).

Table 2. Reliability analysis

Item CITC Deleted items α Cronbach αof each construct Cronbach α

STR_WLE_ADJ 0.637 0.656 0.770 0.795

OPT_WLE_ADJ 0.598 0.698

EMO_WLE_ADJ 0.583 0.721

SOC_WLE_ADJ 0.611 0.662 0.762

ASS_WLE_ADJ 0.566 0.712

ENE_WLE_ADJ 0.605 0.669

ST_RELTEACH 0.639 0.470 0.707

ST_FEEDBACK 0.389 0.771

ST_STUCLASS 0.563 0.568

ST_SCHENGAM 0.812 0.694 0.848

ST_POSEMOT 0.842 0.661

ST_WELLBEING 0.524 0.958

Note: The standardized Cronbach's alpha coefficients are 0.773, 0.763, 0.706, and 0.847

Factor structure

From Table 3 (page 16), some loadings show potential cross-loadings (e.g., ENE_WLE_ADJ loads on both Factor 1 and Factor 3 at .525 and .590 respectively). This deserves discussion, particularly given the interpretation of clearly separated constructs.

Under ideal conditions, exploratory factor analysis should yield high loadings for each measured variable on only one latent factor, thereby clearly delineating distinct theoretical constructs. The cross-loading of ENE_WLE_ADJ suggests that the trait of "energy" may not belong exclusively to the domain of skills for interacting with others, as originally hypothesized in the model. Theoretically, a high level of energy (manifested as vitality and active engagement) not only serves as a crucial driver of proactive social interactions (e.g., initiating conversations, participating in group activities) but may also represent an internal resource required for effective emotion regulation (e.g., coping with stress, maintaining positive affective states). Therefore, its significant loadings on two factors may reflect the foundational and cross-cutting role of energy within the system of social-emotional skills, suggesting that it potentially underpins both interpersonal engagement and emotional management processes. This does not invalidate the overall four-factor structure but indicates that the two higher-order constructs, namely "skills for interacting with others" and "emotion regulation skills," may exhibit some degree of conceptual overlap and interrelation in practice, rather than being entirely independent. In the discussion, it can be noted that this finding provides empirical insight into the interconnections among social-emotional skills, implying that certain core attributes (such as energy) may serve as a foundation for the co-development of multiple higher-order competencies.

According to the conventional practices and statistical principles of exploratory factor analysis (EFA), the classification of a variable with significant loadings on multiple factors is not arbitrary but follows clear guidelines. The core principle is to assign the variable to the factor on which it has the highest loading. In this study, the variable "ENE_WLE_ADJ" demonstrated a loading of 0.590 on Factor 3 ("social-emotional skills for engaging with others"), which was higher than its loading of 0.525 on Factor 1 ("social-emotional skills for emotion regulation"). Consequently, when interpreting and naming the factors, it should be classified as part of Factor 3. This indicates that the variable shares the greatest variance with the latent construct of "skills for interacting with others" and holds a stronger theoretical association, suggesting that "energy" is primarily utilized to explain students' activeness and initiative in social interactions.

SEM and model fit

On page 17–18, the manuscript states that “as long as a portion of the indicators meet the standard is sufficient.” This interpretation is methodologically problematic. In addition:

•RMSEA is not reported.

•Chi-square statistics are not reported.

•TLI is not included.

•The reported CFI (0.904) is acceptable but not strong.

The incremental fit indices (CFI, NFI, IFI) met the minimum criteria, indicating that the model demonstrated a significant improvement over the baseline model. The parsimony fit indices (PGFI, PNFI, PCFI) were all greater than 0.50, which is generally considered acceptable. These indices, which penalize model complexity, suggested that the model achieved a reasonable balance between complexity and goodness of fit. The absolute fit index (SRMR) satisfied the commonly used criterion of being less than 0.08, indicating that the residuals of the model were relatively small. Model evaluation followed the principle of holistic judgment; the combination of indices reported in the text constituted a mutually corroborating and logically consistent chain of evidence, collectively demonstrating that the model fit was statistically acceptable. The basic plausibility of the model was confirmed, thereby supporting subsequent path analysis.

Furthermore, with large sample sizes, indices such as the chi-square test of model fit are typically rejected; therefore, this study relied on other fit indices and selectively reported certain indicators. Nevertheless, the existing combination of indices indicated that the model fit was statistically acceptable and could support subsequent structural equation modeling analyses, although it did not achieve perfect fit. This indeed represents a limitation of the current study, and future research should provide more comprehensive evidence of model fit.

Given PLOS ONE’s emphasis on technical rigor, a more cautious and methodologically grounded interpretation of model fit is necessary.

Extremely high coefficients

Some standardized loadings are extremely high, particularly:

•Student development → ST_POSEMOT (β = 0.991)

•Student development → ST_SCHENGAM (β = 0.926)

A loading of 0.991 suggests near-perfect overlap between latent factor and indicator. This raises questions about:

•Construct redundancy,

•Conceptual overlap,

•Potential issues in model specification.

This should at least be acknowledged and discussed.

In the model of this study, some observed variables exhibited excessively high factor loadings on their respective latent variables (e.g., the loadings of positive affect at school and student engagement in school on positive development approached 0.99). This may suggest insufficient statistical discrimination between these indicators and the latent variable, indicating potential construct redundancy or conceptual overlap. Although the overall model fit was adequate, these extremely high loadings warrant a cautious interpretation of the measurement validity fo

Attachment Submitted filename: Response to Reviewers.docx

# Peer review: Decision Letter 1

**DOI:** 10.1371/journal.pone.0353972.r003

**Authors:** Carlotta Rivella

15 May 2026

PONE-D-25-65886R1

Exploring the impact path of school connectedness on student development through cultivating students' social emotional skills

PLOS One

Dear Dr. Wang,

Thank you for submitting your manuscript to PLOS ONE. After careful consideration, we feel that it has merit but does not fully meet PLOS ONE’s publication criteria as it currently stands. Therefore, we invite you to submit a revised version of the manuscript that addresses the points raised during the review process.

While the reviewers acknowledge the merit of the revisions made, they still suggest further improvements regarding both the introduction and the discussion of the results, as well as the statistical section.

Please submit your revised manuscript by Jun 29 2026 11:59PM. If you will need more time than this to complete your revisions, please reply to this message or contact the journal office at plosone@plos.org. When you're ready to submit your revision, log on to https://www.editorialmanager.com/pone/ and select the 'Submissions Needing Revision' folder to locate your manuscript file.

Please include the following items when submitting your revised manuscript:

- A letter that responds to each point raised by the academic editor and reviewer(s). You should upload this letter as a separate file labeled 'Response to Reviewers'.
- A marked-up copy of your manuscript that highlights changes made to the original version. You should upload this as a separate file labeled 'Revised Manuscript with Track Changes'.
- An unmarked version of your revised paper without tracked changes. You should upload this as a separate file labeled 'Manuscript'.

If you would like to make changes to your financial disclosure, please include your updated statement in your cover letter. Guidelines for resubmitting your figure files are available below the reviewer comments at the end of this letter.

If applicable, we recommend that you deposit your laboratory protocols in protocols.io to enhance the reproducibility of your results. Protocols.io assigns your protocol its own identifier (DOI) so that it can be cited independently in the future. For instructions see: https://journals.plos.org/plosone/s/submission-guidelines#loc-laboratory-protocols. Additionally, PLOS ONE offers an option for publishing peer-reviewed Lab Protocol articles, which describe protocols hosted on protocols.io. Read more information on sharing protocols at https://plos.org/protocols?utm_medium=editorial-email&utm_source=authorletters&utm_campaign=protocols.

As the corresponding author, your ORCID iD is verified in the submission system and will appear in the published article. PLOS supports the use of ORCID, and we encourage all coauthors to register for an ORCID iD and use it as well. Please encourage your coauthors to verify their ORCID iD within the submission system before final acceptance, as unverified ORCID iDs will not appear in the published article. *Only*  the individual author can complete the verification step; PLOS staff *cannot*  verify ORCID iDs on behalf of authors.

We look forward to receiving your revised manuscript.

Kind regards,

Carlotta Rivella

Guest Editor

PLOS One

Journal Requirements:

1. If the reviewer comments include a recommendation to cite specific previously published works, please review and evaluate these publications to determine whether they are relevant and should be cited. There is no requirement to cite these works unless the editor has indicated otherwise.

2. Please review your reference list to ensure that it is complete and correct. If you have cited papers that have been retracted, please include the rationale for doing so in the manuscript text, or remove these references and replace them with relevant current references. Any changes to the reference list should be mentioned in the rebuttal letter that accompanies your revised manuscript. If you need to cite a retracted article, indicate the article’s retracted status in the References list and also include a citation and full reference for the retraction notice.

[Note: HTML markup is below. Please do not edit.]

Reviewers' comments:

Reviewer's Responses to Questions

**Comments to the Author**

1. If the authors have adequately addressed your comments raised in a previous round of review and you feel that this manuscript is now acceptable for publication, you may indicate that here to bypass the “Comments to the Author” section, enter your conflict of interest statement in the “Confidential to Editor” section, and submit your "Accept" recommendation.

Reviewer #1: All comments have been addressed

Reviewer #2: (No Response)

**********

2. Is the manuscript technically sound, and do the data support the conclusions?

The manuscript must describe a technically sound piece of scientific research with data that supports the conclusions. Experiments must have been conducted rigorously, with appropriate controls, replication, and sample sizes. The conclusions must be drawn appropriately based on the data presented.

Reviewer #1: Partly

Reviewer #2: Partly

**********

3. Has the statistical analysis been performed appropriately and rigorously?

Reviewer #1: No

Reviewer #2: No

**********

4. Have the authors made all data underlying the findings in their manuscript fully available?

The PLOS Data policy requires authors to make all data underlying the findings described in their manuscript fully available without restriction, with rare exception (please refer to the Data Availability Statement in the manuscript PDF file). The data should be provided as part of the manuscript or its supporting information, or deposited to a public repository. For example, in addition to summary statistics, the data points behind means, medians and variance measures should be available. If there are restrictions on publicly sharing data—e.g. participant privacy or use of data from a third party—those must be specified.

Reviewer #1: Yes

Reviewer #2: Yes

**********

5. Is the manuscript presented in an intelligible fashion and written in standard English?

PLOS ONE does not copyedit accepted manuscripts, so the language in submitted articles must be clear, correct, and unambiguous. Any typographical or grammatical errors should be corrected at revision, so please note any specific errors here.

Reviewer #1: Yes

Reviewer #2: Yes

**********

6. Review Comments to the Author

Please use the space provided to explain your answers to the questions above. You may also include additional comments for the author, including concerns about dual publication, research ethics, or publication ethics. (Please upload your review as an attachment if it exceeds 20,000 characters)

Reviewer #1: Review of revised manuscript (PONE-D-25-65886R1)

The revised manuscript addresses an important and timely research question using a large international dataset (OECD SSES 2023). The authors have made several improvements in response to the first round of review, particularly regarding data availability, clarification of ethical aspects, and the inclusion of additional measurement indices (e.g., CR, AVE, discriminant validity).

However, despite these revisions, several substantial methodological and reporting issues remain insufficiently addressed, particularly with regard to statistical rigor and the interpretation of SEM results. These issues limit the extent to which the manuscript can currently be considered technically sound.

Below are the main points that still require attention.

Major comments

1.

Model fit reporting and interpretation (pp. 34–36) Although additional fit indices have been included, some key indicators are still missing, in particular:

• RMSEA (with confidence interval)

• Chi-square statistic and degrees of freedom

The manuscript continues to justify model adequacy based on a selective set of indices, stating that a “holistic judgment” is sufficient. This interpretation remains problematic. Standard SEM practice requires complete and transparent reporting of model fit indices, and conclusions should be based on the overall pattern of indices rather than partial compliance.

In addition, the reported values (e.g., CFI ≈ 0.90; TLI ≈ 0.90; SRMR = 0.081) indicate an acceptable but not strong fit, which should be acknowledged more explicitly rather than presented as fully satisfactory.

2.

Treatment of the hierarchical data structure (p. 30; Table 1) The dataset includes students nested within different contexts (five cities); however:

• No control variables for site are included

• No multigroup or multilevel analyses have been conducted

• No correction for clustering has been applied

This issue was already raised in the first round of review and is now mentioned only as a limitation. However, given the size and structure of the dataset, it remains a methodologically relevant omission.

At a minimum, the authors should:

• include site (and, if available, other covariates such as gender or socioeconomic status) as control variables or

• provide a stronger methodological justification for not modeling the nested structure or

• at least indicate, in the future directions section, alternative analytical approaches and the use of statistical techniques that account for the hierarchical nature of the data

3.

Extremely high factor loadings (pp. 35–36) Some loadings remain extremely high (e.g., student development → positive emotions β ≈ 0.99). The manuscript acknowledges this as a limitation, which is appropriate.

However, the implications are not fully developed. Values of this magnitude suggest:

• possible construct redundancy

• limited discriminant validity at the higher-order level

A more cautious interpretation of the “student development” construct is needed, with a more explicit discussion of the potential overlap among indicators.

4.

Use of EFA with theoretically defined constructs (pp. 31–33) The manuscript now includes both EFA and CFA. However, the rationale for conducting EFA remains weak, considering that:

• the constructs are defined a priori (OECD framework)

• a confirmatory approach would be expected

The current justification (EFA followed by CFA) does not fully resolve the initial concern. The authors should either:

• provide a clearer methodological rationale for this choice or

• emphasize the confirmatory framework more consistently

5.

Conceptual framing and consistency (Introduction and Discussion) The cultural dimension (Asian context), introduced in the Introduction, remains insufficiently developed in the Discussion.

6.

Causal language There has been some improvement in reducing causal language (e.g., increased use of “predicts” and “is associated with”). However, some formulations still suggest directional or causal interpretations that are not fully supported by the cross-sectional design.

Further refinement toward strictly associational language is recommended.

Minor comments

• The manuscript is clearer than in the previous version, but language and style still require revision, particularly to reduce repetition and improve fluency.

• Some sections remain overly verbose or redundant, especially in the Results.

Overall evaluation The manuscript has improved in several respects, particularly in terms of transparency (data availability, ethical clarification) and partial strengthening of the measurement model. However, for the reasons outlined above, further revisions are still required

Reviewer #2: Introduction

The theoretical framework is presented primarily in the Method section, rather than being clearly and systematically articulated in the Introduction. In its current form, the Introduction remains comparatively long and would benefit from further condensation.

There is a reference to the framework of self-determination theory, even though the stated theoretical point of reference is social cognitive theory.

The following passage also seems inappropriate for the Introduction, as it reads more like an interpretation of results or a contribution statement that would be better placed in the Discussion or removed:

“This finding not only confirms that school connectedness predicts student development through the dual mediation of interpersonal and emotion regulation skills, but also elucidates the hierarchical and sequential nature of skill development—specifically, that interpersonal competence serves as an effective precursor to the development of emotion regulation. By clarifying the internal transmission process of “environment → skill sequence → developmental outcome,” this study provides a theoretical foundation and precise targets for school-based interventions designed around the sequential cultivation of social-emotional skills”

Literature review

Later in the literature review, the manuscript states:

“It is worth noting that the empirical model in this study is designed to test a specific, sequential pathway of influence, namely, to explore how school coNNNectedness influences interpersonal skills, which in turn affects emotional regulation skills, ultimately contributing to positive student development. This represents a causal chain model proposed for examination from a particular theoretical perspective (social cognitive theory). While it does not deny the existence of bidirectional relationships, it focuses on testing whether this specific directional pathway is significant and quantifying its effect size.”

In my opinion, this paragraph is misplaced. It reads more as a justification of the analytic model and would fit better in the introduction after the research question.

Materials and methods

The amount of missing data is still unclear and should be explicitly reported.

The statement “the data were assumed to approximately meet the analytical requirements” does not provide sufficient information regarding normality assumptions, and no robustness checks are reported.

Results & discussion

Although a CFA has now been added, the manuscript still does not justify the initial use of EFA.

The cross-loading of the wellbeing factor was acknowledged, but its implications were not adequately examined. In particular, the manuscript does not discuss whether this pattern may indicate conceptual overlap, weak construct specification, or possible model misspecification, nor does it consider alternative modeling solutions.

Comment from first review round: Given the hierarchical structure of the data (students nested within schools, and schools nested within countries), multilevel analyses would be methodologically advisable. At a minimum, statistical controls for site, gender, age/grade, and socioeconomic status (if available) should be included to account for contextual variability.

At present, this issue has been addressed only as a limitation and not at the analytical level.

Because the analysis is based on cross-sectional, observational survey data, the manuscript should avoid causal wording. The reported SEM can identify associations compatible with mediation, but it cannot establish causal mediation or temporal ordering.

The authors state that RMSEA, chi-square statistics, and TLI were omitted because they slightly deviated from recommended thresholds. However, key SEM fit indices should be reported transparently regardless of whether they meet conventional criteria. Any deviations should be acknowledged and discussed as part of the overall model-fit evaluation.

General comments

More generally, the manuscript would benefit from careful streamlining and stylistic polishing. A thorough round of proofreading and corrections would enhance readability and may also help remove residual errors (e.g. Subsequently, student interpersonal skills are hypothesized not only to directly enhance their positive development but also to further promote their emotion regulationl skills.)

**********

7. PLOS authors have the option to publish the peer review history of their article (what does this mean?). If published, this will include your full peer review and any attached files.

If you choose “no”, your identity will remain anonymous but your review may still be made public.

**Do you want your identity to be public for this peer review?**  For information about this choice, including consent withdrawal, please see our Privacy Policy.

Reviewer #1: No

Reviewer #2: No

**********

[NOTE: If reviewer comments were submitted as an attachment file, they will be attached to this email and accessible via the submission site. Please log into your account, locate the manuscript record, and check for the action link "View Attachments". If this link does not appear, there are no attachment files.]

To ensure your figures meet our technical requirements, please review our figure guidelines: https://journals.plos.org/plosone/s/figures

You may also use PLOS’s free figure tool, NAAS, to help you prepare publication quality figures: https://journals.plos.org/plosone/s/figures#loc-tools-for-figure-preparation.

NAAS will assess whether your figures meet our technical requirements by comparing each figure against our figure specifications.

Attachment Submitted filename: Review of revised manuscript.pdf

# Author comment: Author response to Decision Letter 2

**DOI:** 10.1371/journal.pone.0353972.r004

27 May 2026

Reviewer #1: Review of revised manuscript (PONE-D-25-65886R1)

The revised manuscript addresses an important and timely research question using a large international dataset (OECD SSES 2023). The authors have made several improvements in response to the first round of review, particularly regarding data availability, clarification of ethical aspects, and the inclusion of additional measurement indices (e.g., CR, AVE, discriminant validity).

However, despite these revisions, several substantial methodological and reporting issues remain insufficiently addressed, particularly with regard to statistical rigor and the interpretation of SEM results. These issues limit the extent to which the manuscript can currently be considered technically sound.

Below are the main points that still require attention.

Major comments

1.

Model fit reporting and interpretation (pp. 34–36) Although additional fit indices have been included, some key indicators are still missing, in particular:

• RMSEA (with confidence interval)

• Chi-square statistic and degrees of freedom

The manuscript continues to justify model adequacy based on a selective set of indices, stating that a “holistic judgment” is sufficient. This interpretation remains problematic. Standard SEM practice requires complete and transparent reporting of model fit indices, and conclusions should be based on the overall pattern of indices rather than partial compliance.

In addition, the reported values (e.g., CFI ≈ 0.90; TLI ≈ 0.90; SRMR = 0.081) indicate an acceptable but not strong fit, which should be acknowledged more explicitly rather than presented as fully satisfactory.

The required indicator data was provided as requested, along with explanatory notes and discussions. For example, results that exactly met the standard threshold were described as acceptable rather than completely ideal. For results that deviated from the standard values or slightly exceeded the threshold, these were also presented, discussed, and explained.

The specific main text is as follows:

The model fitting is acceptable. The model fitting indicators are as follows. In this study, CFI=0.904>0.9, NFI=0.904>0.9, IFI=0.904>0.9, PGFI=0.570>0.5, PNFI=0.685>0.5, PCFI=0.685>0.5. Goodness-of-fit indices should typically exceed 0.9 [38]. Indices exceeding 0.50 serves as the minimum acceptable threshold for models, representing the most fundamental baseline. When indices meet or exceed 0.50, the model demonstrates basic explanatory power. These findings indicate that this study satisfies the standards for acceptable fit [39, 40, 41]. The incremental fit indices (CFI, NFI, IFI) met the minimum criteria, indicating a significant improvement of the proposed model over the baseline model. The parsimony fit indices (PGFI, PNFI, PCFI) were all above 0.50, which is generally considered acceptable. These indices penalize model complexity and suggest that the model achieves a reasonable balance between complexity and goodness-of-fit.

In this study, χ²/df = 358. Under conditions of a very large sample size, the chi-square test is almost always significant, and the chi-square-to-degrees-of-freedom ratio becomes excessively inflated; consequently, the model fit based on the chi-square test is typically rejected. Fit indices such as the RMSEA and TLI are substantially influenced by model complexity, sample size, and the distributional characteristics of the observed variables. Given the extremely large sample size and the relatively large number of observed indicators in the model, the RMSEA in this study was 0.123. Although slightly above conventional thresholds, this value is considered acceptable, and its 90% confidence interval [0.110, 0.129] indicates a relatively stable estimate. The TLI was 0.874, approaching the common benchmark of 0.90, and is deemed acceptable given the complexity of the model. The SRMR was 0.109, slightly above the recommended threshold of 0.10. The SRMR reflects the average discrepancy between the model-implied covariance matrix and the sample covariance matrix. In models involving a large number of observed variables (12 in this study) and complex path relationships, a slight elevation in the SRMR is a common phenomenon. An SRMR value below 0.10 is generally considered acceptable, while values between 0.10 and 0.12 can be regarded as marginally acceptable.

Furthermore, in this study, all hypothesized paths were statistically significant (p<0.001), with standardized path coefficients ranging from 0.187 to 0.782, consistent with theoretical expectations. The R²values for the endogenous latent variables were as follows: Engaging with others (0.611), Emotion regulation (0.503), and School performance (0.379), indicating that the model exhibited moderate to strong explanatory power for the core variables. Model evaluation adhered to the principle of holistic judgment. Although some fit indices in this study did not reach the optimal levels suggested by conventional benchmarks, the overall model demonstrated acceptable fit given the practical constraints of an extremely large sample size and a complex measurement structure.

Model evaluation adhered to the principle of holistic judgment. Although some fit indices in this study did not reach the optimal levels suggested by conventional benchmarks, the overall model demonstrated acceptable fit given the practical constraints of an extremely large sample size and a complex measurement structure.

2.

Treatment of the hierarchical data structure (p. 30; Table 1) The dataset includes students nested within different contexts (five cities); however:

• No control variables for site are included

• No multigroup or multilevel analyses have been conducted

• No correction for clustering has been applied

This issue was already raised in the first round of review and is now mentioned only as a limitation. However, given the size and structure of the dataset, it remains a methodologically relevant omission.

At a minimum, the authors should:

• include site (and, if available, other covariates such as gender or socioeconomic status) as control variables or

• provide a stronger methodological justification for not modeling the nested structure or

• at least indicate, in the future directions section, alternative analytical approaches and the use of statistical techniques that account for the hierarchical nature of the data

In response to the revision suggestions, this study added relevant content that includes city and gender as control variables in the structural equation model analysis.

The specific main text is as follows:

Based on this, this study included students’ city of residence and gender as control variables in the structural equation model. After scaling the observed variables to reduce differences in variable scales, the structural equation model with site and gender as control variables converged normally. The results showed that all core structural paths remained significant. To improve numerical stability, the observed indicators were rescaled before SEM estimation. WLE-based indicators were divided by 100, and student scale indicators were divided by 10. Standardized coefficients were used for interpretation.

The data for this study were collected from five cities: Delhi (India), Dubai (UAE), Gunma (Japan), Jinan (China), and Kudus (Indonesia), with Delhi (India, DEL) serving as the reference category. When the control variable is a five-level categorical variable, including four dummy variables allows for complete group comparisons, with the omitted category automatically serving as the reference group. Its effect is absorbed into the model intercept and therefore does not appear in the path coefficient estimates. Accordingly, the model included four categorical control variables for the cities (DUB, GUN, JIN, KUD) and one demographic control variable for gender (gender_Std). The MLR estimator was used to examine the effects on paths among latent variables. The variance inflation factor (VIF) for all control variables was below 3, indicating no serious multicollinearity concerns.

Regarding the effects of control variables on Engaging with others, the standardized effect for DUB was 0.044, indicating that respondents in this group exhibited relatively higher levels of Engaging with others. The effect for GUN was -0.221 (p<0.001), representing the strongest negative influence among the control variables, suggesting that students from Gunma had significantly lower levels of Engaging with others. The effect for JIN was not statistically significant (β=0.005, p=0.589). The effect for KUD was 0.019 (p=0.033), indicating a weak positive influence. The standardized effect for gender was 0.092 (p<0.001), indicating that male students exhibited higher levels of Engaging with others compared to female students.

In terms of effects on Emotion regulation, the effect for DUB was not significant (β=-0.008, p=0.371). GUN showed a significant positive effect (β=0.148), indicating that students from Gunma had higher levels of Emotion regulation. The effects for JIN (β=0.072) and KUD (β=0.115) both positively predicted Emotion regulation. Gender also demonstrated a positive effect (β=0.093, p<0.001), with male students showing relatively higher levels of Emotion regulation.

Regarding effects on Student development, the effect for DUB was 0.038 (p<0.001), indicating a weak positive influence. The effect for GUN was 0.027 (p=0.006), representing a relatively weak positive influence. The effect for JIN was not statistically significant (β=0.003, p=0.797). The positive effect for KUD was the most pronounced (β=0.174, p<0.001), making it the strongest control variable influencing Student development, suggesting that students from Kudus demonstrated better developmental outcomes. The effect for gender was 0.013 (p=0.026), indicating only a weak predictive role.

In summary, the results of the control variables indicate regional differences. Using Delhi as the reference group, students from Gunma showed significantly lower levels of Engaging with others, whereas students from Kudus demonstrated the most prominent performance in Student development. Students from Jinan exhibited no significant differences from the reference group across any dimension. Students from Dubai showed slightly higher levels only in the Engaging with others dimension compared to the reference group. At the demographic level, male students exhibited a weak but statistically significant advantage across all dimensions. Overall, the differential impact of geographical and cultural backgrounds on student development was more pronounced than that of gender.

3.

Extremely high factor loadings (pp. 35–36) Some loadings remain extremely high (e.g., student development → positive emotions β ≈ 0.99). The manuscript acknowledges this as a limitation, which is appropriate.

However, the implications are not fully developed. Values of this magnitude suggest:

• possible construct redundancy

• limited discriminant validity at the higher-order level

A more cautious interpretation of the “student development” construct is needed, with a more explicit discussion of the potential overlap among indicators.

In accordance with the revision suggestions, corresponding explanatory notes have been added.

The specific main text is as follows:

The high factor loading of Student positive emotion (close to 1.0) may indicate potential construct redundancy or substantial conceptual overlap with the latent variable of student positive development. Although school engagement and student wellbeing were both included in the study, student positive emotion almost entirely represents the overall latent construct of student positive development. This phenomenon may be attributed to the strong theoretical interrelatedness of this indicator with the other two dimensions (particularly school engagement), or it may suggest that, given the current measurement tools and sample, the three dimensions are empirically difficult to distinguish, thereby compromising the discriminant validity of the higher-order construct. Therefore, when interpreting the path effects of student positive development in this study, one should avoid equating it simply with a broad, multidimensional developmental state. Instead, it is more appropriate to understand it as a locally composite indicator dominated by student positive emotion, while also incorporating elements of academic engagement behaviors and basic wellbeing.

4.

Use of EFA with theoretically defined constructs (pp. 31–33) The manuscript now includes both EFA and CFA. However, the rationale for conducting EFA remains weak, considering that:

• the constructs are defined a priori (OECD framework)

• a confirmatory approach would be expected

The current justification (EFA followed by CFA) does not fully resolve the initial concern. The authors should either:

• provide a clearer methodological rationale for this choice or

• emphasize the confirmatory framework more consistently

As requested, the corresponding part of EFA has been removed, and only CFA was conducted.

5.

Conceptual framing and consistency (Introduction and Discussion) The cultural dimension (Asian context), introduced in the Introduction, remains insufficiently developed in the Discussion.

As requested, the research findings were discussed in the context of the Asian scenario.

The specific main text is as follows:

From an Asian perspective, education systems across the region generally emphasize collectivism and relational harmony. Against this backdrop, peer relationships and teacher-student relationships not only serve as core components of school belonging but also function as the “relational soil” for the development of students’ social-emotional skills. “Student-peer relationships” and “perceived teacher-student relationships” contribute most significantly to school belonging, which aligns closely with Asian cultural traditions that value interpersonal bonds and respect for authority (e.g., respect for teachers and moral education) [49] (Stremfel et al., 2024). Asian students’ sense of school belonging relies more on high-quality interpersonal interactions than on mere institutional affiliation [50].

Asian students generally face high levels of academic pressure [51]. Among emotion regulation skills, “stress tolerance” and “optimism” are particularly prominent in predicting positive development. This finding may reflect the “adaptive function” of emotion regulation skills within the Asian cultural context. Students require not only general emotional control abilities but also specific skills to cope with pressure and maintain positive expectations in highly competitive and evaluative educational environments [52].

Because Asian cultures encourage self-regulation and interpersonal restraint within the collective, students first obtain group identification and emotional support through positive interpersonal interactions (e.g., cooperation, empathy), which then become internalized as individual-level emotion management abilities [53]. This contrasts with the Western individualistic approach, where emotion regulation is often cultivated as an independent individual skill. Therefore, the model proposed in this study is not only statistically valid but also culturally interpretable [54].

The facilitating effect of school belonging on social-emotional skills is not a universal linear process; rather, it presents a culturally specific chain-mediated pathway within the Asian educational ecology characterized by collectivism, high academic pressure, and interpersonal orientation.

Urban differences can be cross-interpreted through dimensions such as cultural value variations, educational system orientations, and social support structures. The significantly lower performance of students in Gunma, Japan, on “Engaging with others” may be related to the “high-context restraint” characteristic of East Asian collectivist culture. Social norms in Japanese society emphasize individual integration into the collective rather than proactively expanding interpersonal connections, leading students to prefer maintaining distance and avoiding excessive self-disclosure in interactions. Such cultural traits tend to suppr

Attachment Submitted filename: Response_to_Reviewers_auresp_2.docx

# Peer review: Decision Letter 2

**DOI:** 10.1371/journal.pone.0353972.r005

**Authors:** Carlotta Rivella

16 Jun 2026

PONE-D-25-65886R2

Exploring the impact path of school connectedness on student development through cultivating students' social emotional skills

PLOS One

Dear Dr. Wang,

Thank you for submitting your manuscript to PLOS ONE. After careful consideration, we feel that it has merit but does not fully meet PLOS ONE’s publication criteria as it currently stands. Therefore, we invite you to submit a revised version of the manuscript that addresses the points raised during the review process.

==============================

The reviewers and I acknowledge that the authors have carefully addressed the concerns raised during the previous rounds of review and that the manuscript has been substantially strengthened as a result. However, a few minor revisions are still required before the manuscript can be considered for publication. In particular:

-  The article would benefit from another round of proofreading (There are some mistakes: e.g., The commonly used maximum likelihood estimation in SEM typically assumes that the observed data are derived from a multivariate normal distribution. IN THIS STUDY. This study conducted a normality test to examine whether the quantitative data followed a normal distribution.).

Also in the section related to robustness checks, it is written: "In this study, the absolute values of kurtosis were less than 10". However, according to the table ASS_WLE_ADJ = 11.240

- some methodological concerns remain, particularly with respect to the interpretation of SEM results and the overall adequacy of model fit. The reported values (RMSEA = .123, TLI = .874, SRMR = .109) remain indicative of a model that demonstrates only marginal to moderate fit. While the authors appropriately acknowledge some of these limitations, the interpretation remains somewhat optimistic. The limitations associated with model fit should be explicitly recognized when discussing the strength of the conclusions

==============================

Please submit your revised manuscript by **Jul 31 2026 11:59PM** . If you will need more time than this to complete your revisions, please reply to this message or contact the journal office at plosone@plos.org. When you're ready to submit your revision, log on to https://www.editorialmanager.com/pone/ and select the 'Submissions Needing Revision' folder to locate your manuscript file.

Please include the following items when submitting your revised manuscript:

- A letter that responds to each point raised by the academic editor and reviewer(s). You should upload this letter as a separate file labeled 'Response to Reviewers'.
- A marked-up copy of your manuscript that highlights changes made to the original version. You should upload this as a separate file labeled 'Revised Manuscript with Track Changes'.
- An unmarked version of your revised paper without tracked changes. You should upload this as a separate file labeled 'Manuscript'.

If you would like to make changes to your financial disclosure, please include your updated statement in your cover letter. Guidelines for resubmitting your figure files are available below the reviewer comments at the end of this letter.

If applicable, we recommend that you deposit your laboratory protocols in protocols.io to enhance the reproducibility of your results. Protocols.io assigns your protocol its own identifier (DOI) so that it can be cited independently in the future. For instructions see: https://journals.plos.org/plosone/s/submission-guidelines#loc-laboratory-protocols. Additionally, PLOS ONE offers an option for publishing peer-reviewed Lab Protocol articles, which describe protocols hosted on protocols.io. Read more information on sharing protocols at https://plos.org/protocols?utm_medium=editorial-email&utm_source=authorletters&utm_campaign=protocols.

As the corresponding author, your ORCID iD is verified in the submission system and will appear in the published article. PLOS supports the use of ORCID, and we encourage all coauthors to register for an ORCID iD and use it as well. Please encourage your coauthors to verify their ORCID iD within the submission system before final acceptance, as unverified ORCID iDs will not appear in the published article. *Only*  the individual author can complete the verification step; PLOS staff *cannot*  verify ORCID iDs on behalf of authors.

We look forward to receiving your revised manuscript.

Kind regards,

Carlotta Rivella

Guest Editor

PLOS One

Journal Requirements:

If the reviewer comments include a recommendation to cite specific previously published works, please review and evaluate these publications to determine whether they are relevant and should be cited. There is no requirement to cite these works unless the editor has indicated otherwise.

Please review your reference list to ensure that it is complete and correct. If you have cited papers that have been retracted, please include the rationale for doing so in the manuscript text, or remove these references and replace them with relevant current references. Any changes to the reference list should be mentioned in the rebuttal letter that accompanies your revised manuscript. If you need to cite a retracted article, indicate the article’s retracted status in the References list and also include a citation and full reference for the retraction notice.

[Note: HTML markup is below. Please do not edit.]

Reviewers' comments:

Reviewer's Responses to Questions

**Comments to the Author**

1. If the authors have adequately addressed your comments raised in a previous round of review and you feel that this manuscript is now acceptable for publication, you may indicate that here to bypass the “Comments to the Author” section, enter your conflict of interest statement in the “Confidential to Editor” section, and submit your "Accept" recommendation.

Reviewer #1: All comments have been addressed

Reviewer #2: All comments have been addressed

**********

2. Is the manuscript technically sound, and do the data support the conclusions?

The manuscript must describe a technically sound piece of scientific research with data that supports the conclusions. Experiments must have been conducted rigorously, with appropriate controls, replication, and sample sizes. The conclusions must be drawn appropriately based on the data presented.

Reviewer #1: Yes

Reviewer #2: Yes

**********

3. Has the statistical analysis been performed appropriately and rigorously?

Reviewer #1: Yes

Reviewer #2: (No Response)

**********

4. Have the authors made all data underlying the findings in their manuscript fully available?

The PLOS Data policy requires authors to make all data underlying the findings described in their manuscript fully available without restriction, with rare exception (please refer to the Data Availability Statement in the manuscript PDF file). The data should be provided as part of the manuscript or its supporting information, or deposited to a public repository. For example, in addition to summary statistics, the data points behind means, medians and variance measures should be available. If there are restrictions on publicly sharing data—e.g. participant privacy or use of data from a third party—those must be specified.

Reviewer #1: Yes

Reviewer #2: Yes

**********

5. Is the manuscript presented in an intelligible fashion and written in standard English?

PLOS ONE does not copyedit accepted manuscripts, so the language in submitted articles must be clear, correct, and unambiguous. Any typographical or grammatical errors should be corrected at revision, so please note any specific errors here.

Reviewer #1: Yes

Reviewer #2: Yes

**********

6. Review Comments to the Author

Please use the space provided to explain your answers to the questions above. You may also include additional comments for the author, including concerns about dual publication, research ethics, or publication ethics. (Please upload your review as an attachment if it exceeds 20,000 characters)

Reviewer #1: The authors have made substantial efforts to address the concerns raised in the previous review round. In particular, they improved the transparency of model reporting, included additional measurement information, removed the exploratory factor analysis, incorporated control variables, expanded the discussion of cultural aspects, and reduced the use of causal language.

The manuscript has been strengthened considerably compared to the previous version. While some concerns remain regarding the overall quality of model fit and the limitations associated with the SEM results, these issues are now reported and discussed more explicitly. Although the reported fit indices do not indicate an optimal model fit, the authors have acknowledged these limitations and provided additional justification for their analytical choices.

Overall, I believe that the major concerns raised in the previous review have been adequately addressed and that the manuscript is now suitable for publication.

Reviewer #2: The article would benefit from another round of proofreading (There are some mistakes: e.g., The commonly used maximum likelihood estimation in SEM typically assumes that the observed data are derived from a multivariate normal distribution. IN THIS STUDY. This study conducted a normality test to examine whether the quantitative data followed a normal distribution.)

Also in the section related to robustness checks, it is written: "In this study, the absolute values of kurtosis were less than 10". However, according to the table ASS_WLE_ADJ = 11.240

**********

7. PLOS authors have the option to publish the peer review history of their article (what does this mean?). If published, this will include your full peer review and any attached files.

If you choose “no”, your identity will remain anonymous but your review may still be made public.

**Do you want your identity to be public for this peer review?**  For information about this choice, including consent withdrawal, please see our Privacy Policy.

Reviewer #1: No

Reviewer #2: No

**********

[NOTE: If reviewer comments were submitted as an attachment file, they will be attached to this email and accessible via the submission site. Please log into your account, locate the manuscript record, and check for the action link "View Attachments". If this link does not appear, there are no attachment files.]

To ensure your figures meet our technical requirements, please review our figure guidelines: https://journals.plos.org/plosone/s/figures

You may also use PLOS’s free figure tool, NAAS, to help you prepare publication quality figures: https://journals.plos.org/plosone/s/figures#loc-tools-for-figure-preparation.

NAAS will assess whether your figures meet our technical requirements by comparing each figure against our figure specifications.

# Author comment: Author response to Decision Letter 3

**DOI:** 10.1371/journal.pone.0353972.r006

30 Jun 2026

- The article would benefit from another round of proofreading (There are some mistakes: e.g., The commonly used maximum likelihood estimation in SEM typically assumes that the observed data are derived from a multivariate normal distribution. IN THIS STUDY. This study conducted a normality test to examine whether the quantitative data followed a normal distribution.).

All revisions in this Revised Manuscript with Track Changes are highlighted in blue.

The entire manuscript has undergone another round of proofreading for grammar, expression, and data accuracy.

The confusing sentence in the preceding text has been deleted. Keep only this sentence: “This study conducted a normality test to examine whether the quantitative data followed a normal distribution”.

Also in the section related to robustness checks, it is written: "In this study, the absolute values of kurtosis were less than 10". However, according to the table ASS_WLE_ADJ = 11.240

Only the kurtosis value for ASS_WLE_ADJ was 11.240, which, nonetheless, remained well below the liberal threshold (|kurtosis| < 20). The kurtosis values for all other variables fell within reasonable ranges. Accordingly, the data can be considered to exhibit acceptable univariate normality, supporting the use of maximum likelihood estimation.

- some methodological concerns remain, particularly with respect to the interpretation of SEM results and the overall adequacy of model fit. The reported values (RMSEA = .123, TLI = .874, SRMR = .109) remain indicative of a model that demonstrates only marginal to moderate fit. While the authors appropriately acknowledge some of these limitations, the interpretation remains somewhat optimistic. The limitations associated with model fit should be explicitly recognized when discussing the strength of the conclusions.

Given that several fit indices of the structural equation model reached only acceptable levels, the model demonstrated marginal to moderate fit, falling short of an optimal level and merely meeting the threshold for acceptability. Therefore, while the analysis of the significance and directional consistency of the path coefficients can be considered highly reliable, the interpretation of effect sizes for each path should be approached with caution and appropriate flexibility, and should be positioned as exploratory findings. Comparisons regarding the magnitude of effects across different paths warrant further refinement through model modification or validation using independent samples.

Attachment Submitted filename: Response_to_Reviewers_auresp_3.docx

# Editor report: Decision Letter 3

**DOI:** 10.1371/journal.pone.0353972.r007

**Authors:** Carlotta Rivella

1 Jul 2026

<p>Exploring the impact path of school connectedness on student development through cultivating students’ social-emotional skills

PONE-D-25-65886R3

Dear Dr. Wang

We’re pleased to inform you that your manuscript has been judged scientifically suitable for publication and will be formally accepted for publication once it meets all outstanding technical requirements.

Within one week, you’ll receive an e-mail detailing the required amendments. When these have been addressed, you’ll receive a formal acceptance letter and your manuscript will be scheduled for publication.

An invoice will be generated when your article is formally accepted. Please note, if your institution has a publishing partnership with PLOS and your article meets the relevant criteria, all or part of your publication costs will be covered. Please make sure your user information is up-to-date by logging into Editorial Manager at Editorial Manager® and clicking the ‘Update My Information' link at the top of the page. For questions related to billing, please contact billing support.

If your institution or institutions have a press office, please notify them about your upcoming paper to help maximize its impact. If they’ll be preparing press materials, please inform our press team as soon as possible -- no later than 48 hours after receiving the formal acceptance. Your manuscript will remain under strict press embargo until 2 pm Eastern Time on the date of publication. For more information, please contact onepress@plos.org.

Kind regards,

Carlotta Rivella

Guest Editor

PLOS One

# Editor report: Acceptance letter

**DOI:** 10.1371/journal.pone.0353972.r008

**Authors:** Carlotta Rivella

PONE-D-25-65886R3

PLOS One

Dear Dr. Wang,

I'm pleased to inform you that your manuscript has been deemed suitable for publication in PLOS One. Congratulations! Your manuscript is now being handed over to our production team.

At this stage, our production department will prepare your paper for publication. This includes ensuring the following:

* All references, tables, and figures are properly cited

* All relevant supporting information is included in the manuscript submission,

* There are no issues that prevent the paper from being properly typeset

You will receive further instructions from the production team, including instructions on how to review your proof when it is ready. Please keep in mind that we are working through a large volume of accepted articles, so please give us a few days to review your paper and let you know the next and final steps.

Lastly, if your institution or institutions have a press office, please let them know about your upcoming paper now to help maximize its impact. If they'll be preparing press materials, please inform our press team within the next 48 hours. Your manuscript will remain under strict press embargo until 2 pm Eastern Time on the date of publication. For more information, please contact onepress@plos.org.

You will receive an invoice from PLOS for your publication fee after your manuscript has reached the completed accept phase. If you receive an email requesting payment before acceptance or for any other service, this may be a phishing scheme. Learn how to identify phishing emails and protect your accounts at https://explore.plos.org/phishing.

If we can help with anything else, please email us at customercare@plos.org.

Thank you for submitting your work to PLOS ONE and supporting open access.

Kind regards,

PLOS ONE Editorial Office Staff

on behalf of

Dr. Carlotta Rivella

Guest Editor

PLOS One
